# Multi‐dataset identification of innovative feature genes and molecular mechanisms in keratoconus

**DOI:** 10.1111/jcmm.70079

**Published:** 2024-09-19

**Authors:** Ning Lyu, Yiqin Dai, Jiawen Wu, Yidan Fan, Zhaoyuan Lyu, Jiayu Gu, Jingyi Cheng, Jianjiang Xu

**Affiliations:** ^1^ Eye Institute and Department of Ophthalmology Eye & ENT Hospital, Fudan University Shanghai China; ^2^ NHC Key laboratory of Myopia and Related Eye Diseases, Key Laboratory of Myopia and Related Eye Diseases, Chinese Academy of Medical Sciences Eye & ENT Hospital, Fudan University Shanghai China; ^3^ Shanghai Key Laboratory of Visual Impairment and Restoration Eye & ENT Hospital, Fudan University Shanghai People's Republic of China; ^4^ Graduate School of Transdisciplinary Arts Akita University Akita Japan

**Keywords:** *ATOH7*, bioinformatics, immune infiltration, immunotherapeutic target, keratoconus, *MYRF*

## Abstract

This study aimed to identify feature genes and explore the molecular mechanisms of keratoconus (KC). We downloaded data files from NCBI GEO public database. The Limma package was used for differential expression analysis of gene profiles. Lasso regression was used to identify the feature genes. The CIBERSORT algorithm was used to infer the proportion of immune‐infiltrating cells and analyse the correlation between gene expression levels and immune cells. Related transcription factors and miRNAs of key genes were predicted using the Cistrome DB and Mircode databases. Analysis of expression differences in disease genes was based on the GeneCards database. The CMap was used to analyse targeted therapeutic drugs. IHC was performed to verify the expression levels of ATOH7 and MYRF in corneas. Exactly 593 upregulated and 473 downregulated genes were identified. Lasso regression analysis identified *ATOH7*, *DBNDD1*, *RNF217‐AS1*, *ARL11*, *MYRF* and *SNORA74B* as feature genes for KC. All key genes were correlated with immune infiltration and the levels of activated memory CD4+ T cells and plasma cells were significantly increased. miRNA, IRF and STAT families were correlated to feature genes. The expression levels of key genes were significantly correlated to KC‐related genes. Entinostat, ochratoxin‐a, diphencyprone and GSK‐3‐inhibitor‐II were predicted as potential KC medications. The expression of MYRF was significantly higher in the KC samples, contrary to the expression of ATOH7. KC is related to both immune infiltration and genetic factors. *MYRF* and *ATOH7* were newly identified and verified feature genes of KC.

## 1 INTRODUCTION

Keratoconus (KC) is a bilateral asymmetric eye disease characterized by progressive thinning and steepening of the cornea, leading to myopia, irregular astigmatism and significant visual function impairment.^1^, ^2^ Clinically, the central corneal stroma of patients with KC undergoes gradual thinning and loss of structural integrity, which leads to corneal bulging, making the cornea a characteristic cone‐shaped appearance.^3^ Once KC achieves to the severe stage and certifies with excessive ectasia, thinning and scarring, thereby obviously impairing visual acuity, keratoplasty is considered the last resort.^4^ Santodomingo‐Rubido et al., illustrates the prevalence and incidence rates of KC have been approximated to be between 0.2 and 4790 per 100,000 persons and 1.5 and 25 per 100,000 persons/year.^2^ The highest prevalence and incidence rates classically find in individuals between the ages of 20 to 30 years.^5^, ^6^ Thus, KC is one of the leading indications for keratoplasty worldwide and causes low vision disability.^7^, ^8^

Although the pathogenesis of KC remains uncertain, it is mainly considered to be related to genetic and environmental factors.^9^ It has been estimated that individuals with a family history of KC have a 15–67 times higher risk of developing KC than those with no family history.^10^ Further studies on the candidate genes of KC (*SPATA7* ^11^ and *KC6* ^12^) and others^13^ illustrate that mutations in the presence of gene variants are required to elicit keratoconic traits.^14^ Traditionally, KC has been considered as a non‐inflammatory corneal disease.^4^ However, increasing studies have found that immune response and inflammation have important roles in the pathogenesis of KC.^15^ Different kinds of immune cells and inflammatory factors have been found on the ocular surface and circulatory system of patients with KC.^16^ The levels of multiple inflammatory factors in the cornea and tears of patients with KC are significantly up‐regulated, and some of them (e.g. IL‐10, MMP‐9, TGFB1) are unique to KC.^17^ Moreover, the IL‐17 signalling pathway may have an important role in the pathogenesis and development of KC.^18^

Recently, the rapid progress in the development of next‐generation sequencing (NGS) technologies, including bulk RNA sequencing (bulk RNA‐seq) and single‐cell RNA‐sequencing (scRNA‐seq), has shed light on complicated biological systems including cancer genomics and diverse microbial communities.^19^ Bulk RNA‐seq is a method for the transcriptomic analysis of pooled cell populations, tissue sections or biopsies,^20^ which enables comprehensive and rapid access to almost all transcript sequence information in a given tissue or organ of a species in a given state.^21^ scRNA‐seq is used to analyse the expression profile of the cellular transcriptome at the level of a single cell, identify cell‐specific markers, find rare cell types and reveal differential expression between cells. In addition, it has become an effective method to study the cellular heterogeneity of complex organisms.^22^

**FIGURE 1:**
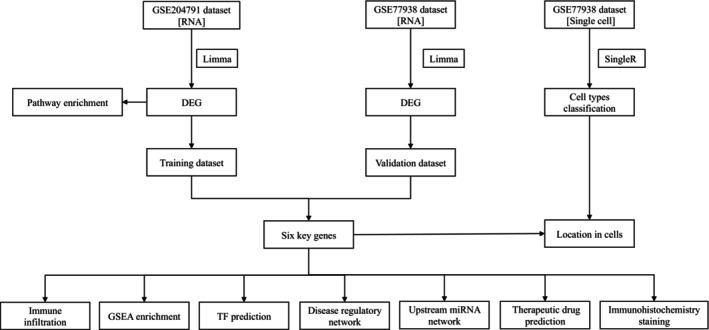
Workflow of identification of key genes analysis in KC. DEG, different expressed genes; TF, transcription factors.

In this research, we discovered innovative feature genes of KC and explored the function of feature genes in immune infiltration and the underlying molecular mechanisms of KC by combining bulk RNA‐seq and scRNA‐seq. The expression levels of ATOH7 and MYRF were verified by immunohistochemical staining of corneas. The flowchart of analysis was shown in Figure 1.

## 2 MATERIALS AND METHODS

### 2.1 Data download

The Series Matrix File data file of GSE204791 was downloaded from the National Center of Biotechnology Information (NCBI) Gene Expression Omnibus (GEO) public database with annotation file GPL21185, which included the expression profile data of 16 patients, including 8 normal and 8 disease groups. The Series Matrix File data file of GSE77938 was downloaded from the NCBI GEO public database with the annotation file GPL18460, which included the expression profile data of 50 patients, including 25 normal and 25 disease groups. Moreover, four sample datasets of GSE146276 were downloaded from the NCBI GEO public database for single‐cell analysis.

### 2.2 Differential expression analysis

The Limma package is an R software package used for the differential expression analysis of gene profiles, which identifies genes with significant differential expression between different comparison groups. We used the R package “Limma” to analyse the different molecular mechanisms of the KC data, identify differentially expressed genes between control and disease samples, select differentially expressed genes with screening criteria of *p* <0.05 & |log2FC| >1, and draw differential gene volcano plots and heat maps.

### 2.3 Functional enrichment analysis

The metascape database (www.metascape.org) was used to perform functional annotation of the differentially expressed genes to explore the relevance among these genes. Gene Ontology (GO) pathway and Kyoto Encyclopedia of Genes and Genomes (KEGG) pathway analysis were performed on differentially expressed genes. A minimum overlap of ≥3 and *p* ≤ 0.01 was considered statistically significant.

### 2.4 Construction of the prediction model

The candidate gene set was selected, and lasso regression was applied to construct a predictive model for the diagnosis of KC. After incorporating the expression values of each specific gene, a risk score formula was constructed for each patient, and the estimated regression coefficients were weighted in the lasso regression analysis. The score for each patient was calculated according to the risk score formula, and the accuracy of the model prediction was verified by receiver operator characteristic (ROC) curve and the value of the area under ROC curve (AUC).

### 2.5 Immune cell infiltration analysis

The CIBERSORT method is widely used to evaluate immune cell types and subtypes in the microenvironment. This method was according to the support vector regression principle, and deconvolution analysis was performed on the expression matrix of immune cell subtypes. It included 547 biomarkers that distinguished 22 human immune cell phenotypes, including T, B, plasma and myeloid cell subgroups. In this study, the CIBERSORT algorithm was used to analyse patient data to evaluate the relative proportions of 22 immune‐infiltrating cells and perform Spearman's correlation analysis between gene expression levels and immune cell content.

### 2.6 Gene set enrichment analysis (GSEA) pathway enrichment analysis

GSEA uses predefined gene sets to rank genes according to their differential expression levels in two types of samples. It then tests whether the pre‐set gene set is enriched at the top or bottom of this ranking table. In this study, GSEA was used to compare the differences in the KEGG pathway between the high‐and low‐expressed samples of feature genes and explore the molecular mechanisms of key genes in the two groups of patients. The permutation number was set to 1000 and the permutation type was set to the phenotype.

### 2.7 Connectivity map (CMap) drug prediction

The CMap is a gene expression profile database developed by the Broad Institute based on interventions in gene expression. It was mainly used to reveal the functional connections between small‐molecule compounds, genes and disease states. It contained gene chip data for 1309 small molecule drugs used to treat five human cell lines. The treatment conditions were diverse and included different drugs, concentrations and treatment durations. In this study, the differentially expressed genes of the disease were used to predict targeted therapeutic drugs for KC.

### 2.8 Single‐cell sequencing analysis

First, the data were processed using the R package named Seurat package and the tSNE algorithm was used to analyse the positional relationships between each cluster. The clusters were annotated to cells that were important for disease development using the Celldex package. Finally, we used the logfc threshold parameter of FindAllMarkers to extract marker genes for each cell subtype from the single‐cell expression profile. Genes with |avg log2FC| >1 and *p* < 0.05 were selected as unique marker genes for each cell subtype.

### 2.9 Immunohistochemistry staining

Experiments on human tissues adhered to the tenets of the Declaration of Helsinki. The experimental protocols were evaluated and approved by the Ethical Committee of the Eye & ENT Hospital, Fudan University (ky2012‐037). Seven KC samples collected from post‐penetrating keratoplasty patients in the operation room were at stage IV (ARC (3‐mm zone) <6.15, PRC (3‐mm zone) <4.95, thinnest pachy <400um, best corrected distance visual acuities (BDVA) < 20/400) according to Belin ABCD Staging/Classification Parameters.^23^ Seven control samples were obtained from healthy corneoscleral rings from residual corneas after use at the Eye Bank of the Eye & ENT Hospital of Fudan University.

For immunohistochemistry (IHC), the dissected tissues were placed in a 59°C constant temperature incubator for 60 min before dewaxing and then immersed in xylene (G20641B, GENERAL‐REAGENT, NJ, US) and anhydrous ethanol (G73537C, GENERAL‐REAGENT, NJ, US) for 10 min. The process was repeated thrice. After washing, the tissues were soaked in 200 mL of 3% H_2_O_2_ and 1 mL of NaN_3_ for 10 min. The mixture was then rinsed with running water. Next, the tissues were immersed in a preheated retrieval solution (WH1034; Weiao, Shanghai, China) and warmed twice for antigen retrieval. After washing, the tissue sections were blocked with 5% bovine serum albumin (BSA, WH2051; Weiao, Shanghai, China) at 25°C for 35 min. Primary antibodies (ATOH7, 1:100, ab229245, Abcam, Cambridge, UK; MYRF, 1:50, PA5‐113555, Thermo Fisher, CA, US) were added to the tissues at 4°C overnight. After washing, the secondary antibody (K5007, DAKO, Glostrup, Denmark) was added to the slides and incubated at 37°C for 35 min. After using 3, 3′‐diaminobenzidine (DAB) enhancing solution (WB0167, Weiao, Shanghai, China) according to the manufacturer's instruction, the tissues were rinsed and counterstained with haematoxylin staining solution (WH1145, Weiao, Shanghai, China). Finally, the tissue sections were rinsed with anhydrous ethanol and xylene, mounted using a mounting medium (WH1161; Weiao, Shanghai, China) and covered with plastic coverslips. Images were captured using a biological microscope (CX43; Olympus, Tokyo, Japan). The positive cell area of each slide representing the protein expression levels of MYRF and ATOH7 was analysed using Image J software (https://imagej.net/ij/, Fiji) under a 20x view according to the existing protocol.^24^

### 2.10 Statistical analysis

All statistical analyses were performed using R software (version 4.2.2). The level of statistical significance was set at *p* < 0.05.

## 3 RESULTS

### 3.1 Identification of differential genes and pathway enrichment

**FIGURE 2:**
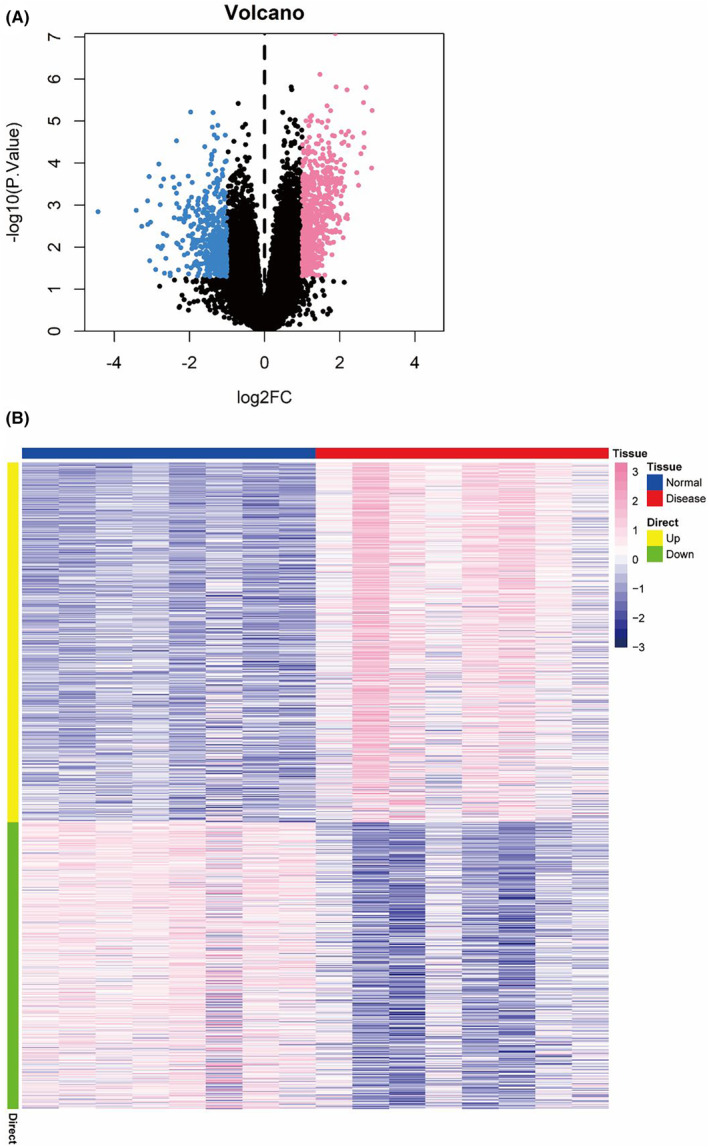
Identification of differential gene expressions in the normal and disease group by Limma package. (A) Screening genes with criteria of *p* < 0.05 & |log2FC| >1. (B) Identification of differentially expressed genes.

**FIGURE 3:**
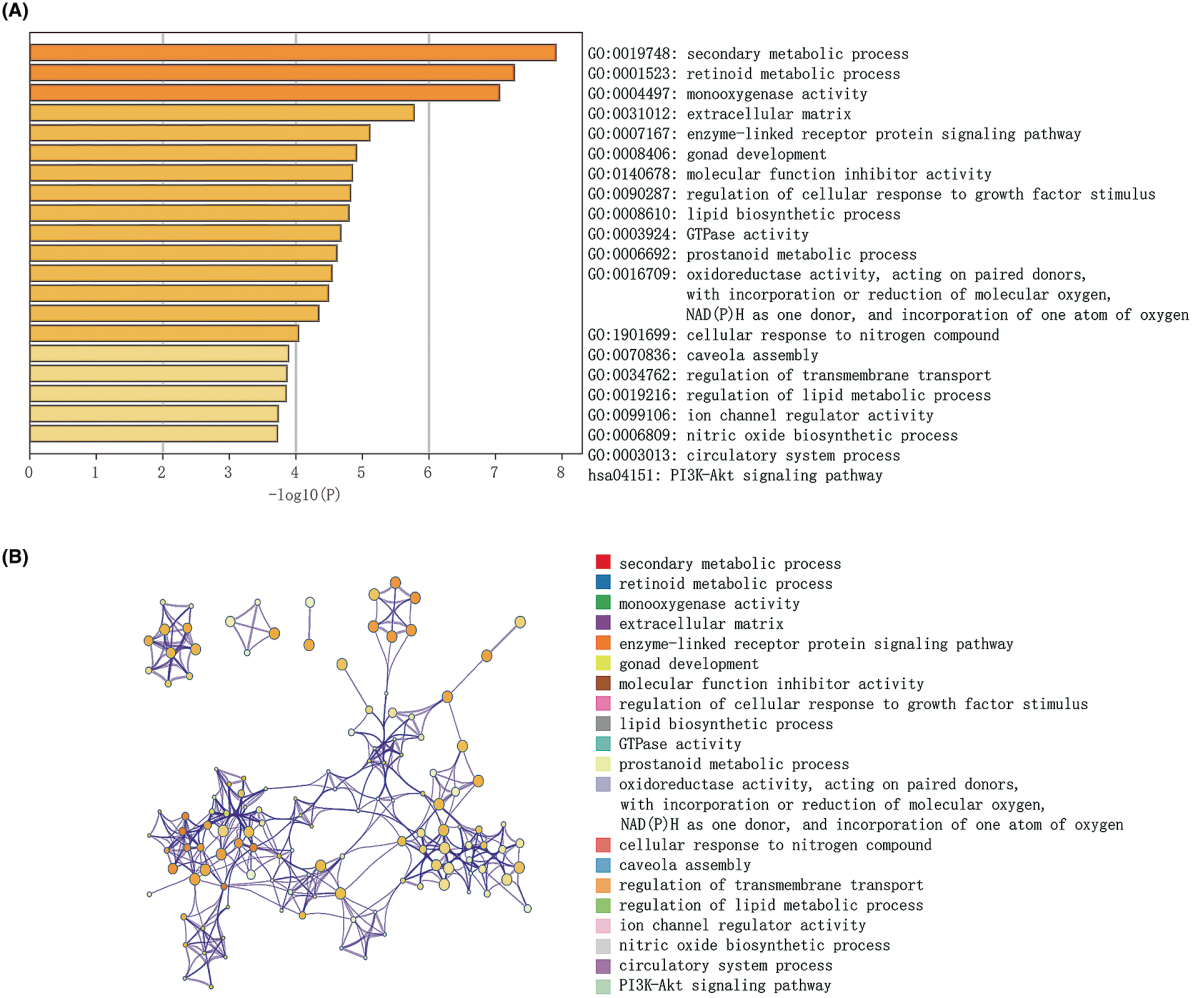
Pathway enrichment of differentially expressed genes based on Metascape database. (A) Primarily enriched pathways and (B) their network.

We downloaded the GSE204791 dataset from the NCBI GEO public database, which included 16 cases: eight in the normal group and eight in the disease group. The Limma package was used to calculate the differentially expressed genes in the two groups with screening criteria of *p* < 0.05 & |log2FC| >1. In total, 1066 differentially expressed genes were identified, including 593 upregulated and 473 downregulated genes (Figure 2A,B), with more details provided in Material S1. Subsequently, we analysed differentially expressed genes using pathway analysis. With the Metascape database, the differentially expressed genes were primarily enriched in pathways such as secondary metabolic processes, retinoid metabolic processes and monooxygenase activity (Figure 3A). The network of these pathways is displayed in Figure 3B.

### 3.2 Identification of key genes and construction of the diagnostic model

**FIGURE 4:**
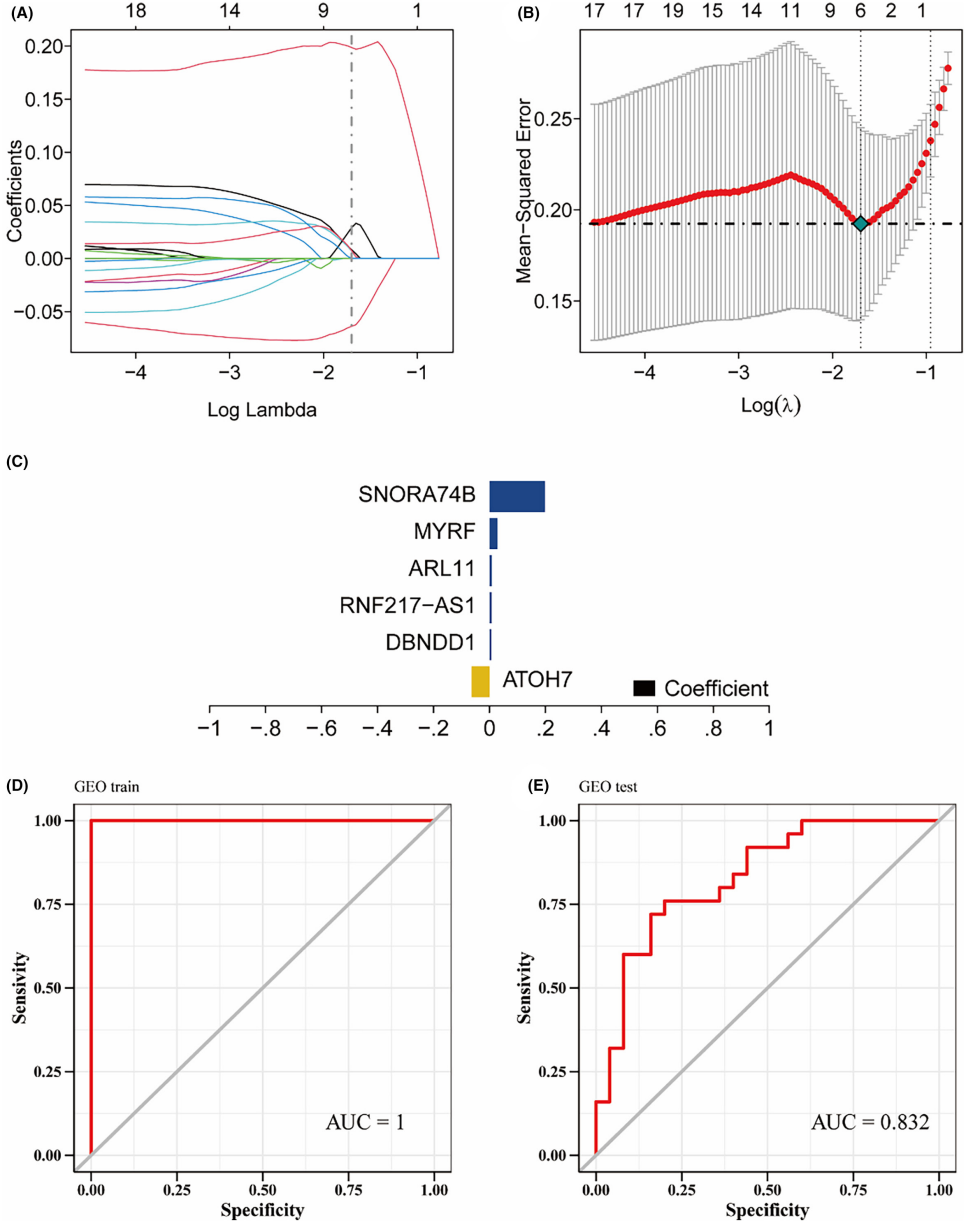
Identification of key genes and construction of predictive model. (A) Ten‐fold cross validation for tuning parameter selection in LASSO model. (B) Distribution of LASSO coefficients for differentially expressed genes. (C) Bar chart of LASSO coefficients for 6 key genes. (D) ROC of predictive model for training set GSE204791 with an AUC of 1 and (E) for validation set GSE77938 with an AUC of 0.832.

We used the GSE204791 and GSE77938 datasets as the training and validation sets, respectively. The differentially expressed genes identified in the previous step were selected for feature selection using Lasso regression. The results showed that six genes were identified by Lasso regression as feature genes for KC, namely *ATOH7*, *DBNDD1*, *RNF217‐AS1*, *ARL11*, *MYRF* and *SNORA74B* (Figure 4A–C). Except for *ATOH7*, the expression levels of the other key genes in the KC group were significantly upregulated (Material S1). These six genes were used as core genes for subsequent experiments and a predictive model for the diagnosis of KC was constructed. The model formula was: RiskScore = ATOH7 × (−0.0638709422255986) + DBNDD1 × 0.00690986717583838 + RNF217‐AS1 × 0.007515606011362 + ARL11 × 0.00814156838823462 + MYRF × 0.0290731999084615 + SNORA74B × 0.198609773078782. The results showed that the predictive model constructed from the six genes had satisfactory diagnostic performance, with an AUC of 1 (Figure 4D). The GSE77938 dataset was used as the validation set to verify the predictive model externally. The results showed that the model had strong stability with an AUC of 0.832 verified by the validation sets GSE77938 (Figure 4E).

### 3.3 Single‐cell analysis of key genes

**FIGURE 5:**
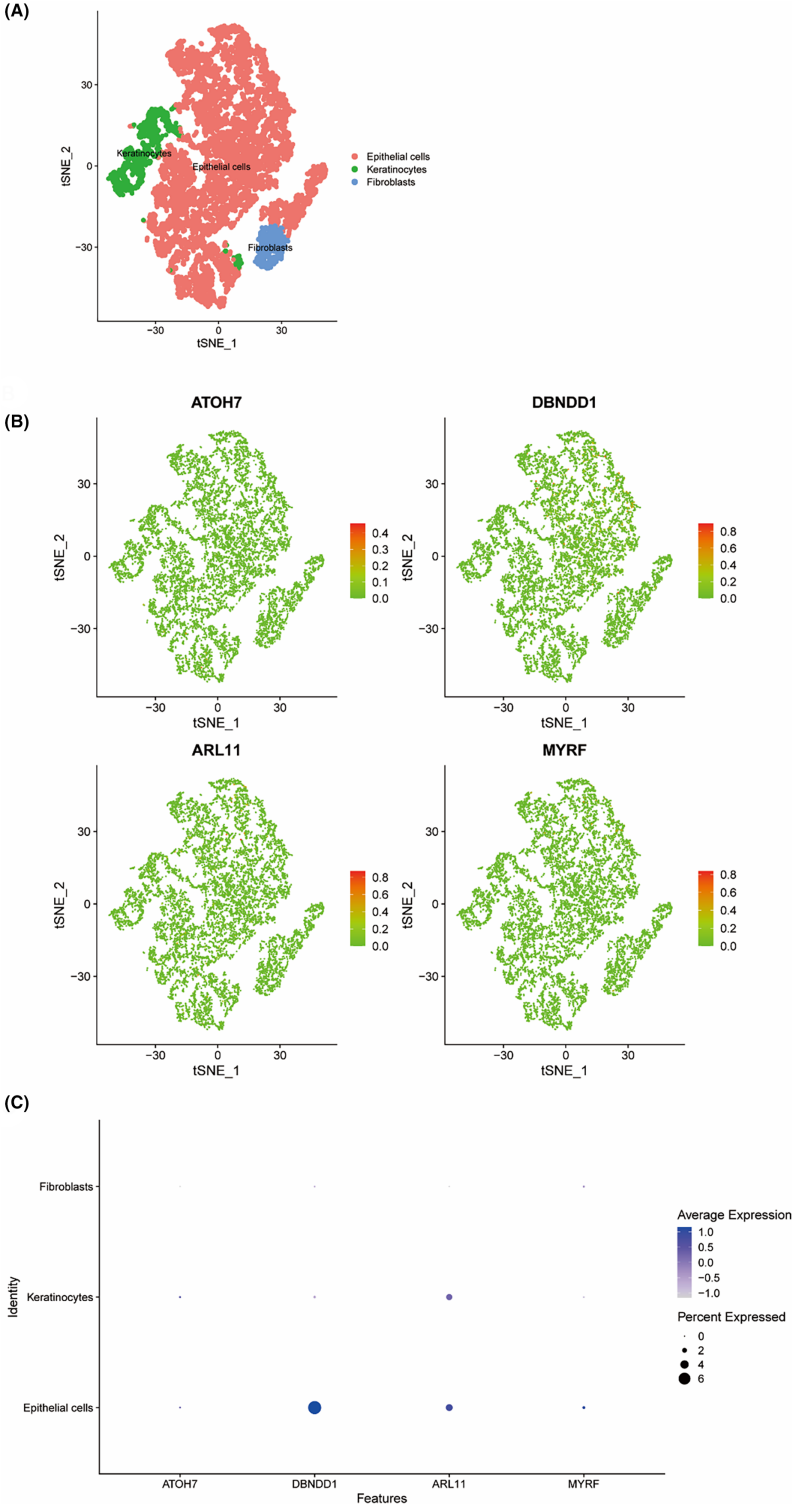
Single‐cell analysis of key genes. (A) Annotation of clusters into three cell categories. (B, C) Expression of key genes in cluster of epithelial cells, keratinocytes and fibroblasts.

We downloaded single‐cell data from GSE146276 and performed single‐cell analysis using the Seurat package. The cells were clustered using the tSNE algorithm, and SingleR in the R package was used to annotate each cluster. All clusters were annotated into three cell categories: epithelial cells, keratinocytes, and fibroblasts. Among these, epithelial cells accounted for the highest proportion (Figure 5A). The expression of key genes in these three cell types was shown in Figure 5B,C. We found that *ATOH7*, *DBNDD1*, *ARL11*, and *MYRF* were all expressed in epithelial cells and the ranking in terms of expression level was *DBNDD1*, *ARL11*, *MYRF* and *ATOH7* (Figure 5B). *ARL11* was the most expressed gene in keratinocytes and lower expression of these feature genes was observed in fibroblasts than in other cell types, except *MYRF* (Figure 5C).

### 3.4 Immune infiltration analysis

**FIGURE 6:**
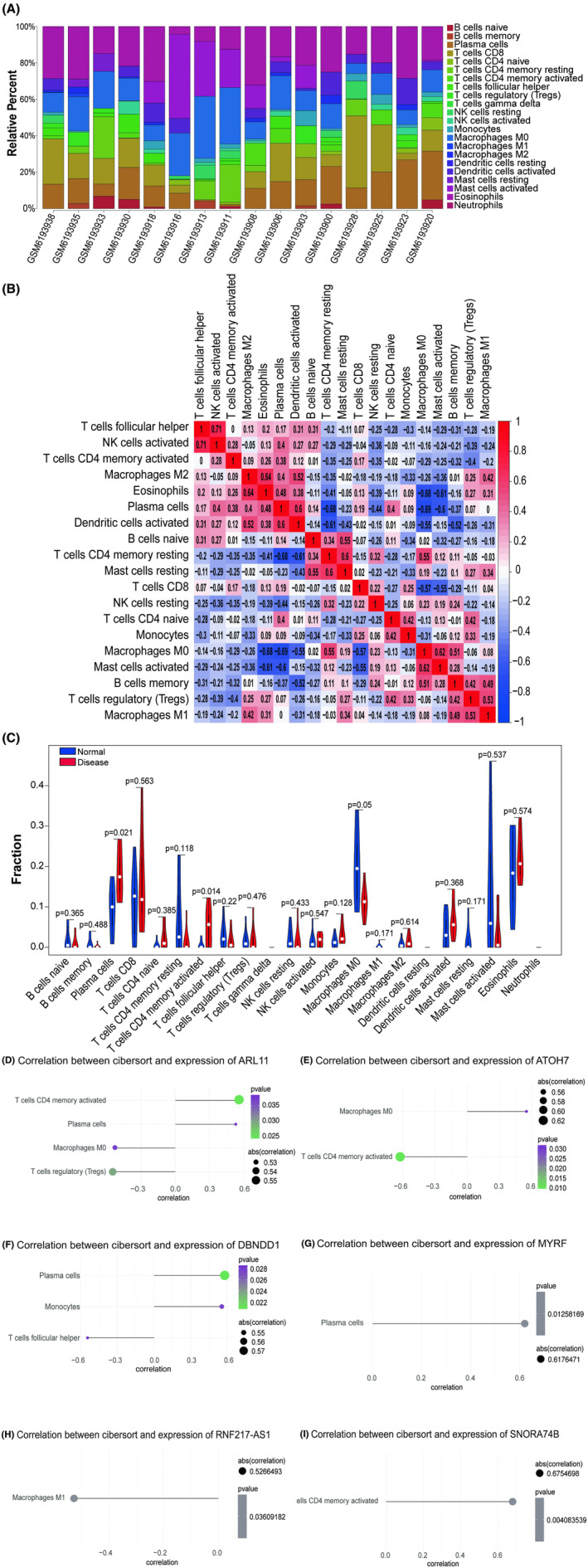
Immune infiltration analysis. (A) Proportion of immune cell content in each sample. (B) Correlations between immune cells. (C) Comparison of immune cells in normal and disease group. (D–I) Correlation between key genes and immune cells.

The immunological microenvironment of the cornea, including the immune cells, cytokines, chemokines, and immune mediators, is usually altered in patients with KC.^17^ By analysing the relationship between key genes and immune infiltration in the KC dataset, we further explored the potential mechanisms by which key genes affect KC progression. The proportion of immune cells in each patient and the correlation between immune cells were shown in Figure 6A,B. Native B cells accounted for the highest proportion in all samples (Figure 6A). There was a positive correlation between the activated memory CD4^+^ T cells and plasma cells (Pearson *r* = 0.38; Figure 6B). In addition, the results showed that the levels of activated memory CD4^+^ T cells and plasma cells were significantly higher in the KC group than in the normal group (*p* < 0.05; Figure 6C). The correlations between the six genes and immune cells are shown in Figure 6D–I. The plasma cells had significantly positive correlations with *ARL11*, *DBNDD1* and *MYRF* (all *p* < 0.05, Figure 6D,F, & G). The activated memory CD4^+^ T cells had significantly positive correlations with *ARL11* and *SNORA74B* (both *p* < 0.05; Figure 6D,I) but negative correlations with *ATOH7* (*p* < 0.01; Figure 6E).

**FIGURE 7:**
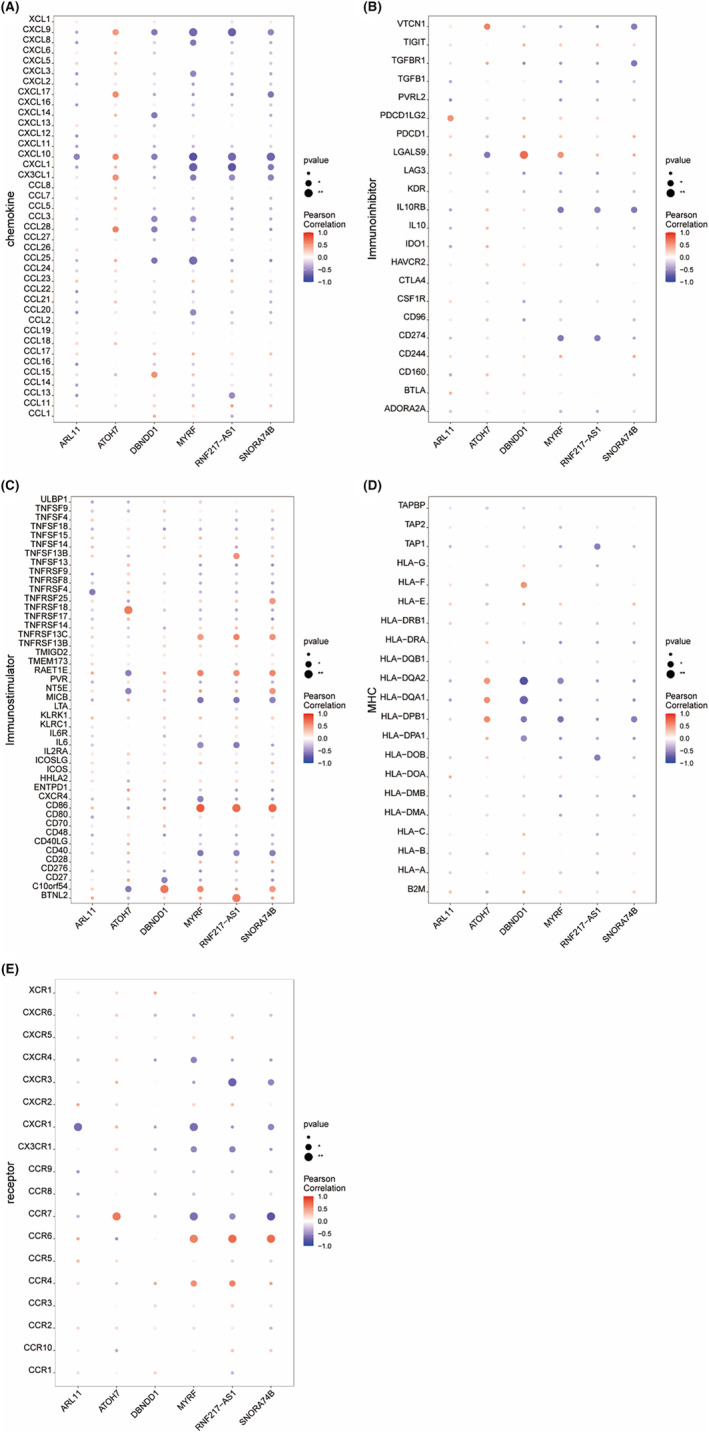
Correlation between key genes and immune factors. (A) Chemokines, (B) Immunoinhibitor, (C) Immunostimulatory, (D)MHC, (E) Cell receptors.

Based on the TISIDB database, we found significant correlations between these six key genes and different immune factors, including chemokines, immunoinhibitors, immunostimulators, MHC and cell receptors (Figure 7A–E). A similar tendency was observed for *MYRF*, *RNF217‐AS1* and *SNORA74B*, with more negative correlations with chemokines, immunoinhibitors, receptors and MHC (Figure 7A,B,D, & E) but stronger positive correlations with immunostimulators compared to other genes, especially CD86 (Pearson *r* = 1, *p* < 0.01; Figure 7C). Furthermore, the correlations between *ATOH7* and immune factors were almost contrary to *MYRF*, with an obvious positive correlation with immunoinhibitors (VTCN1, Pearson *r* = 1, *p* < 0.01; Figure 7B), chemokines and MHC (Figure 7A,B,D). These findings suggested that these key genes were closely relevant to immune cell infiltration and had important roles in the immune microenvironment.

### 3.5 Gene set enrichment analysis

**FIGURE 8:**
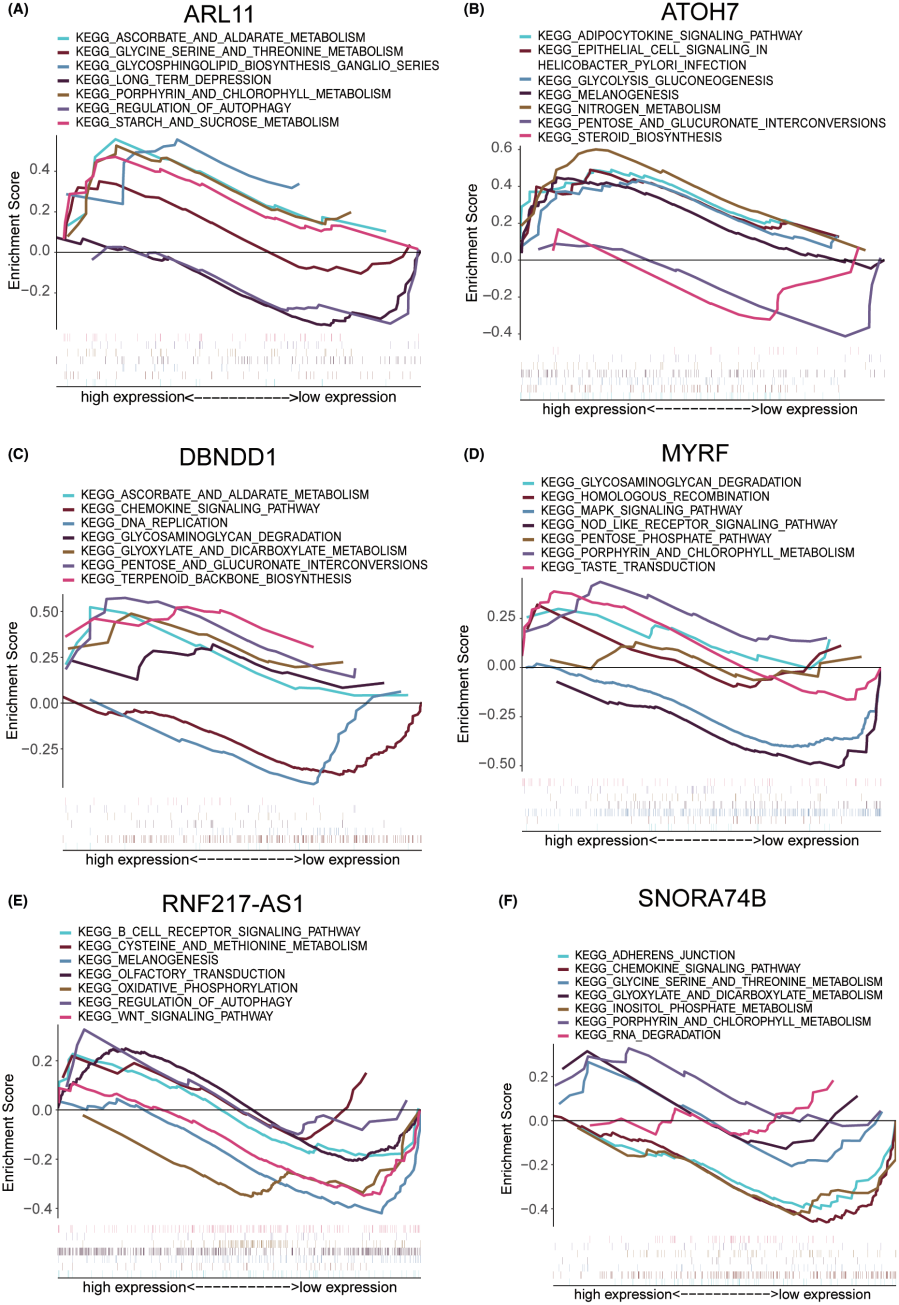
GSAEA of core genes. The pathways enriched in (A) *ARL11*, (B) *ATOH7*, (C) *DBNDD1*, (D) *MYRF*, (E) *RNF217‐AS1* and (F) *SNORA74B*.

Next, we studied the specific signalling pathways enriched in key genes to investigate the potential molecular mechanisms. The complete GSEA results were presented in Material S2. Highly significant pathways were selected for a centralized display. The pathways enriched in *ARL11* included ascorbate and aldarate metabolism, glycine serine and threonine metabolism and glycosphingolipid biosynthesis ganglio series (Figure 8A); the pathways enriched in *ATOH7* included adipocytokine signalling pathway, melanogenesis and nitrogen metabolism (Figure 8B); the pathways enriched in *DBNDD1* included DNA replication, pentose and glucuronate interconversions, and chemokine signalling pathway (Figure 8C); the pathways enriched in *MYRF* included MAPK signalling pathway, pentose phosphate pathway and taste transduction (Figure 8D); the pathways enriched in *RNF217‐AS1* included B cell receptor signalling pathway, melanogenesis and olfactory transduction (Figure 8E); and the pathways enriched in *SNORA74B* included cadherens junction, RNA degradation and inositol phosphate metabolism (Figure 8F).

### 3.6 Prediction of transcription factors

**FIGURE 9:**
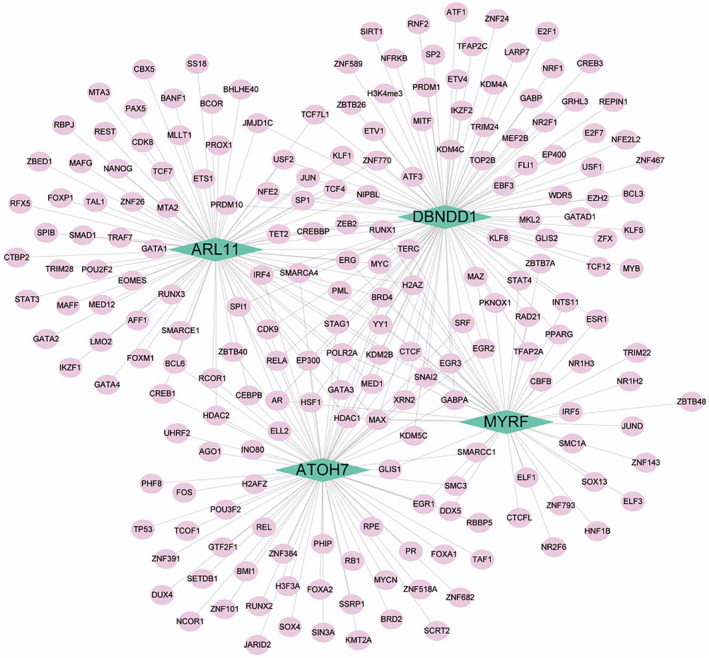
Transcriptional regulatory network of key genes in keratoconus.

We used key genes as the gene set to further explore the transcriptional factor regulatory networks involved in KC. The relevant transcription factors were predicted using the Cistrome DB online database. Among them, 99 transcription factors were predicted for *DBNDD1*, 81 were inferred for *ARL11*, 48 were predicted for *MYRF* and 76 were predicted for *ATOH7* (Figure 9). Several immune response‐related transcription factors were identified in this study. For instance, regarding the IRF family, IRF4 was found in *ATOH7*, *DBNDD1*, *MYRF* and *ARL11* and IRF5 was predicted for *MYRF* (Figure 9). In the STAT family, STAT4 was found in *MYRF* and *DBNDD1*, and STAT3 was found in *ARL11* (Figure 9). We constructed a comprehensive transcriptional regulatory network of key genes in KC using Cytoscape for visualization (Figure 9). Further details were provided in Material S3.

### 3.7 Disease regulatory network of key genes

**FIGURE 10:**
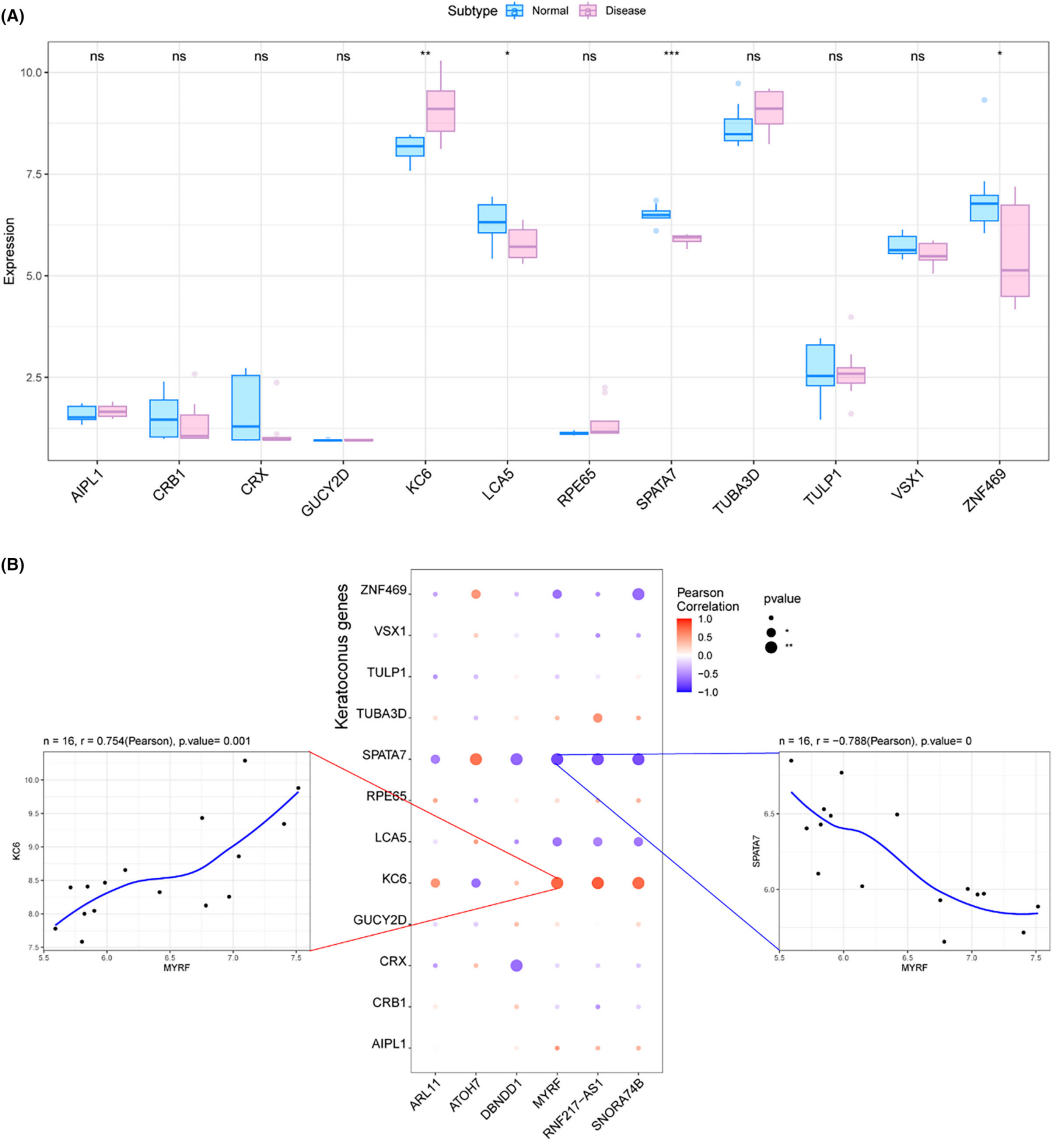
Disease Regulatory Network of Key Genes. (A) Expression differences of disease genes between normal and disease group. (B) Correlation of expression levels between key genes and keratoconus‐related genes.

We obtained 1802 genes related to KC from the GeneCards database (https://www.genecards.org/). Analysis of the expression differences of disease genes revealed that the expression of genes, such as *KC6*, *LCA5*, *SPATA7* and *ZNF469*, differed between the normal and disease groups (Figure 10A). Next, we conducted a correlation analysis between key and KC‐related genes. We found that the expression levels of key genes were significantly correlated with those of KC‐related genes (Figure 10B), with *MYRF* and *SPATA7* showing a significant negative correlation (Pearson *r* = −0.788, *p* < 0.01) and *MYRF* and *KC6* showing a significant positive correlation (Pearson *r* = 0.754, *p* < 0.01). Conversely, *ATOH7* had a significant positive correlation with *SPATA7* (*p* < 0.01; Figure 10B) and a significant negative correlation (*p* < 0.05; Figure 10B) with *KC6*.

### 3.8 Upstream miRNA network analysis and potential therapeutic drug prediction

**FIGURE 11:**
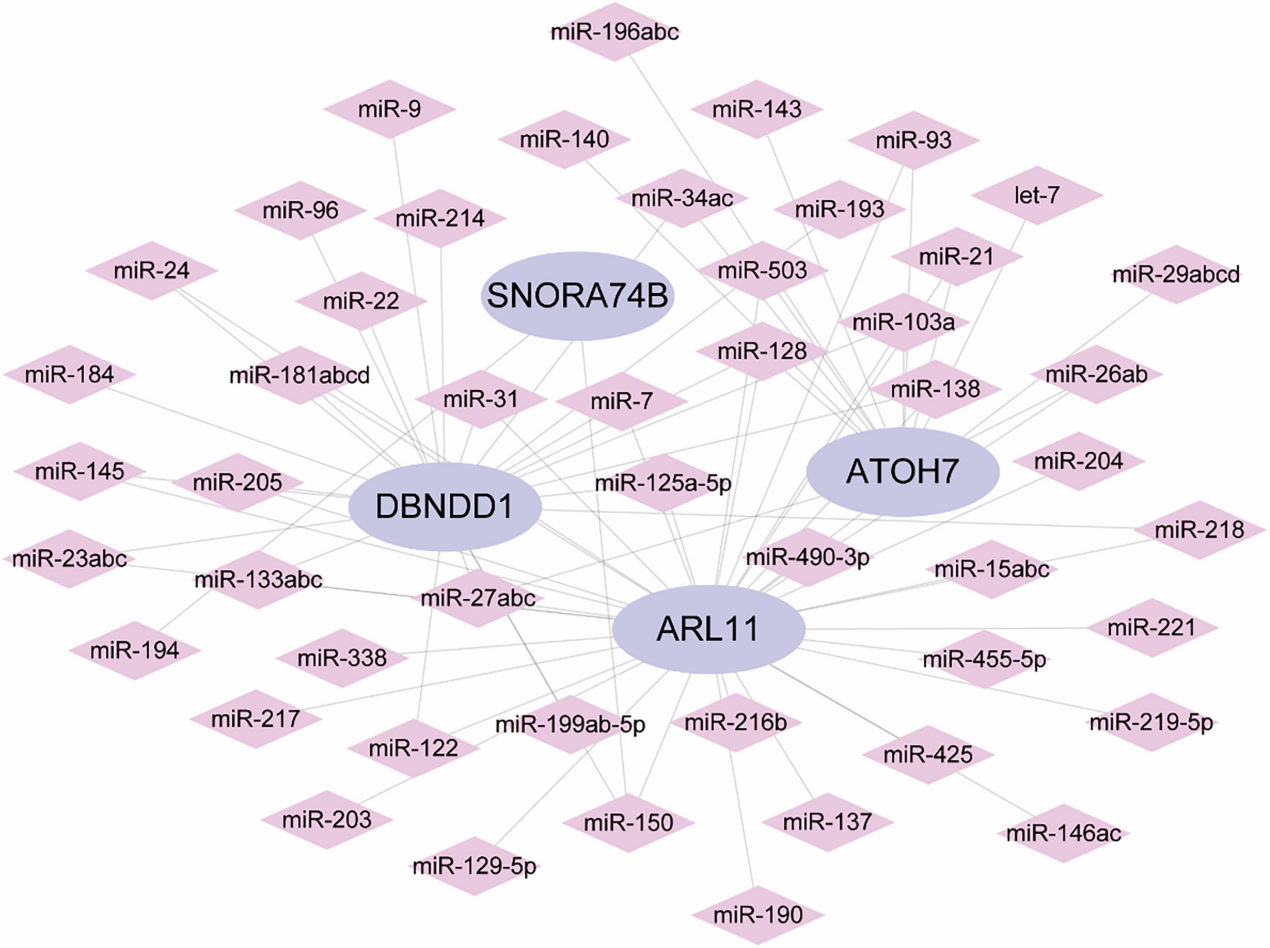
Network of predicted target miRNA related to *ATOH7, DBNDD1*, *SNORA74B* and *ARL11*.

**FIGURE 12:**
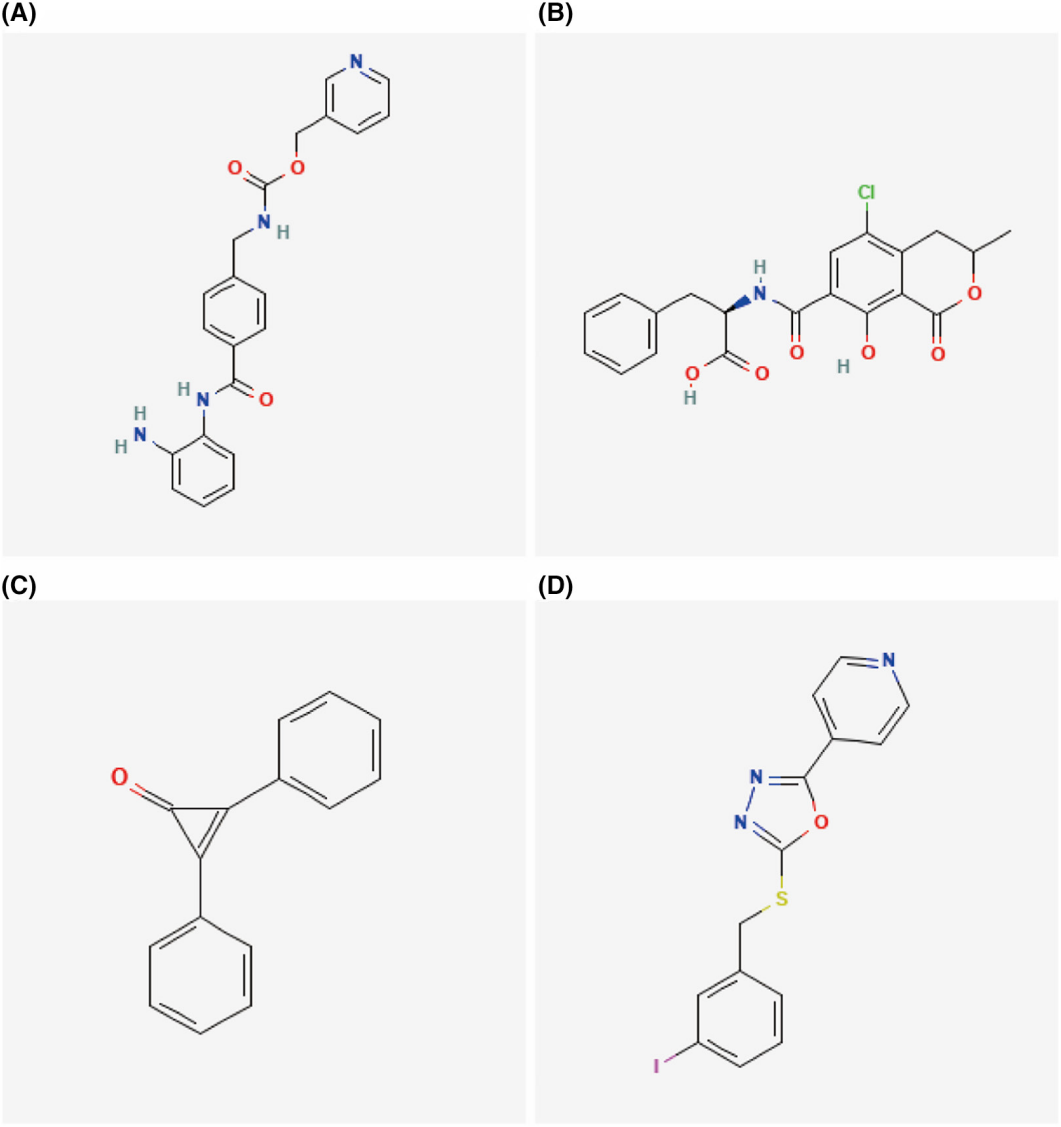
Potential Therapeutic Drug Prediction. (A) Entinostat, (B) Ochratoxin‐a, (C) Diphencyprone and (D) GSK‐3‐inhibitor‐II.

We conducted a reverse prediction of key genes using the Mircode database and obtained 49 miRNAs and 74 mRNA‐miRNA relationship pairs. The results were visualized using Cytoscape (Figure 11). The data showed that miR‐150 had a strong interaction with *DBNDD1*, *SNORA74B* and *ARL11* (Figure 11). We divided the differentially expressed mRNAs into upregulated and downregulated groups and used the CMap database to predict drug targets for the differentially expressed genes. The results showed that the expression profiles perturbed by drugs, such as entinostat (Figure 12A), ochratoxin‐a (Figure 12B), diphencyprone (Figure 12C) and GSK‐3‐inhibitor‐II (Figure 12D), were significantly negatively correlated with disease‐perturbed expression profiles, and the drugs might alleviate or even reverse the disease state. More details on miRNA and drug prediction were provided in Materials S4 & S5.

### 3.9 Immunohistochemistry staining

**FIGURE 13:**
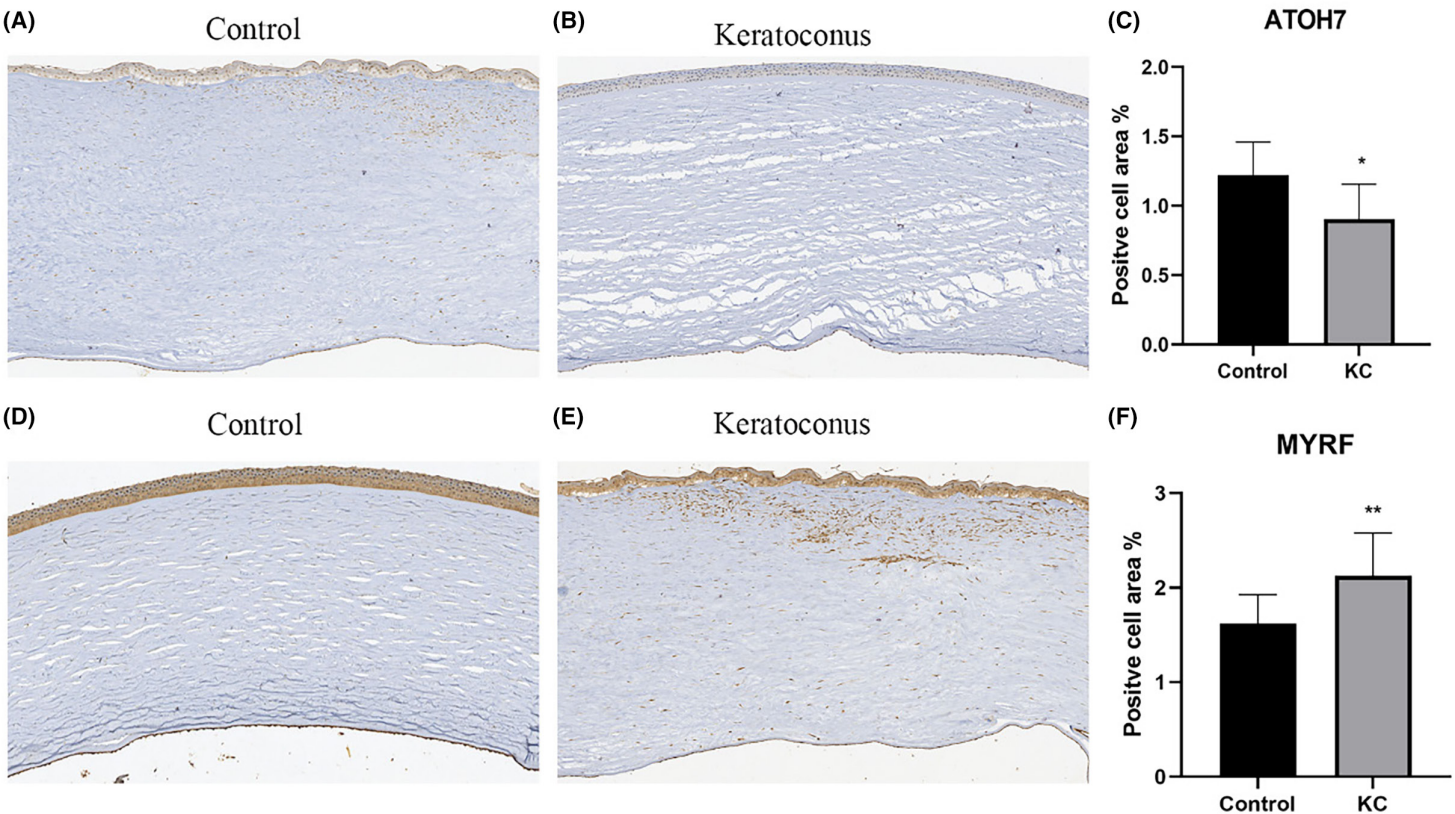
IHC staining of normal and KC corneas. (A, B) Representative images of ATOH7 staining. (C) Expression level of ATOH7. (D, E) Representative images of MYRF staining. (F) Expression level of MYRF. *p < 0.05, **p < 0.01. Scale bar = 200 um.

We verified the expression levels of MYRF and ATOH7 in normal and KC corneal samples through IHC staining. The positive cell area, which represented the gene expression level, was calculated as described in the Method section. Consistent with the results mentioned above, the expression of MYRF was significantly higher in KC samples (2.13%) than in control samples (1.62%) (*p* < 0.01; Figure 13 A–C). In contrast, the expression of ATOH7 was significantly lower in KC samples (0.904%) than in control samples (1.22%) (*p* < 0.05; Figure 13D–F). Both ATOH7 and MYRF were mainly expressed in the epithelium, but more MYRF staining was observed in the stroma of the KC group (Figure 13E), in which fibroblasts accounted for a higher proportion, consistent with the results of the scRNA‐seq analysis shown in Figure 5.

## 4 DISCUSSION

Although various therapies for KC have been developed, including wearing contact/scleral lenses, corneal cross‐linking, phototherapeutic keratectomy and keratoplasty, the outcomes remain unsatisfactory.^4^ Thus, it is essential to conduct in‐depth research on the biological mechanisms and pathogenesis of KC to determine a more effective and efficient therapy for KC.

In this study, we enriched KC‐related pathways using bulk RNA‐seq analysis of differentially expressed genes in KC. Secondary metabolic process pathways^25^ are chemical reactions resulting in many of the chemical syntheses of compounds. The retinoid metabolic process^26^ is associated with the synthesis of structurally similar natural derivatives or synthetic compounds. Monooxygenase activity^27^ is associated with incorporation of one hydroxyl group (‐OH) into substrates in many metabolic pathways and utilized by cells to metabolize arachidonic acid (i.e. eicosatetraenoic acid) into cell signalling molecules to reduce or inactivate initiated signalling molecules. It is known that the abnormal structures of epithelial and stromal tissues in KC might be relevant to the unusual synthesis of structures similar to retinol and retina. Lasso regression identified *ATOH7*, *DBNDD1*, *RNF217‐AS1*, *ARL11*, *MYRF* and *SNORA74B* as feature genes for KC (Figure 4). Constructed from the key genes, the predictive model had an AUC of 1.0 and was verified with an AUC of 0.832, indicating that our findings provided new information regarding KC diagnosis.

The annotation results of scRNA‐seq were analysed, and the key genes (*ATOH7*, *DBNDD1*, *ARL11*, *SNORA74B* and *MYRF*) were not differentially expressed among the cell subsets, suggesting that these genes were not specific biomarkers of the cell subsets. In terms of immune cell composition, native B cells accounted for the highest proportion with a role in the secondary adaptive response and immunological memory in human autoimmune diseases.^28^ Plasma cells and activated memory CD4^+^ T cells of the disease group were significantly higher than those of the normal group and had a positive correlation (Figure 6B,C), indicating that there was immune response in KC. CD4^+^ memory T cells developed by antigen exposure or the cytokine milieu communicate with various immune and non‐immune cellular networks within distinct microenvironments in human chronic autoimmune diseases such as multiple sclerosis.^29^ Similarly, plasma cells produce protective antibodies against infectious agents and pathogenic antibodies in autoimmune diseases like systemic lupus erythematosus.^30^ This could be explained by the abnormal expression of core genes due to the significant positive correlations between plasma cells and *ARL11*, *DBNDD1* and *MYRF* (all *p* < 0.05; Figure 6D,F, G) and the significant positive correlations between activated memory CD4^+^ T cells and *ARL11* and *SNORA74B* (both *p* < 0.05; Figure 6D,I) and negative correlations with *ATOH7* (*p* < 0.01; Figure 6E).

In addition, our analysis revealed that *MYRF*, *RNF217‐AS1* and *SNORA74B* exhibited stronger positive correlations with immunostimulatory factors, such as CD86 (Figure 7C), in contrast to *ATOH7*. These findings indicate that *MYRF*, *RNF217‐AS1* and *SNORA74B* may play significant roles in promoting and maintaining immune activation in KC. CD86 is a member of the immunoglobulin superfamily that is crucial for initiating both innate and adaptive immune responses through the activation of naïve and memory T cells.^31^ Conversely, we observed that *MYRF*, *RNF217‐AS1* and *SNORA74B* also showed more substantial positive correlations with immunoinhibitory factors, such as VTCN1 (Figure 7B), compared to *ATOH7*. VTCN1 functions to suppress T‐cell activation by inhibiting IL‐2 production and influencing the cell cycles of CD4^+^ and CD8^+^ T cells.^32^ Interestingly, the predominant presence of plasma cells and activated memory CD4^+^ T cells, without significant involvement of neutrophils or macrophages, could explain the absence of classic inflammatory signs (e.g. heat, redness, swelling and pain) commonly observed in other inflammatory diseases.^33^ Moreover, the chemokine receptors CCR2 and CCR5, as well as F2RL1 and CXCL5, were involved in the immune regulation process of KC.^34^ D'Souza, Sharon et al. also reported the significantly higher levels of inflammatory cytokine in tear fluid and implied a distinct immuno‐inflammatory component in KC pathogenesis.^35^ The associations between KC and immune‐mediated diseases have also been identified (Hashimoto's thyroiditis and inflammatory skin conditions) and confirmed (atopic conditions, including allergic rash, asthma and bronchial hyperresponsiveness and allergic rhinitis) by Claessens, Janneau L J et al.^36^ Combined with the known papers and our findings, the observed changes in immune cell composition and the presence of immune responses in KC patients might originate from disruptions in the expression of these key genes. Understanding the interplay between these genes and immune modulators is crucial for elucidating the underlying mechanisms of KC and could lead to the development of targeted immunotherapeutic strategies.

To explore the biological mechanism, the GSEA results of the key genes showed an obvious trend of gene enrichment (Figure 8). The MAPK pathway, which is enriched in *MYRF*, may promote inflammatory cytokines in the pathogenesis of KC.^37^ The B cell receptor signalling pathway enriched in *RNF217‐AS1* also implied the immune response related to plasma cells existing in patients with KC.^38^ Relationships between inflammation‐relevant transcription factors (IRF and STAT families) and feature genes were identified in this study. IRF4 was correlated with *ATOH7*, *DBNDD1*, *MYRF* and *ARL11* with a key regulatory function in the mature of human immune cells and the essentiality for the differentiation of T lymphocytes and B lymphocytes as well as certain myeloid cells.^39^ IRF5 related to *MYRF* acts as a molecular switch that decides whether macrophages initiate or inflammation suppress^40^ (Figure 8). With regards to STAT family, STAT4, found in *MYRF* and *DBNDD1*, is essential for the development of Th1 cells from naïve CD4^+^ T cells^41^ and STAT3, found in *ARL11*, is essential for the differentiation of TH17 helper T cells^42^ and could be activated via the phosphorylation of serine 727 by the MAPK pathway^43^ (Figure 9). Other kinds of transcription factors also related to the core genes (Figure 9), such as EBF3^44^ involved in B‐cell differentiation and neurogenesis; FOS^45^ promoting cell migration and metastasis, and NR2F6^46^ negatively regulating T cell receptors also participated in immune response. These findings implied that feature genes may alter the immune microenvironment through regulating transcription factors during KC progress.

In our analysis of the GeneCards database for genes associated with KC, we found a significant negative correlation between MYRF and SPATA7, and a significant positive correlation between MYRF and KC6 (Figure 10). This indicates that MYRF is highly expressed in the KC group, suggesting its potential role in promoting the development of KC. MYRF expression is primarily observed in mature myelinating oligodendrocytes within the central nervous system.^47^ Additionally, MYRF has a critical regulatory impact on immune factors and plays an important role in the immune microenvironment.^48^ Variants in *MYRF* are known to cause autosomal dominant and syndromic nanophthalmos in humans and are crucial for the development of the retina and retinal pigment epithelium (RPE).^49^ These findings highlight the significant role of MYRF in ocular tissue development and its potential contribution to KC.

The miRNAs of the target genes were predicted, among which miR‐150 showed the strongest interaction with *DBNDD1*, *SNORA74B* and *ARL11* (Figure 11). miR‐150 is commonly expressed in mature B and T cells^50^ and attracts and activates immune cells in breast cancer.^51^ The knockout of miR‐150 inhibits spontaneous T cell proliferation and the progress of colitis.^52^ In the cornea, hsa‐miR‐150‐5p plays a regulatory role in corneal epithelial stem cell maintenance by inhibiting the Wnt signalling pathway.^53^ The potential therapeutic drug predicted from key genes showed that entinostat and GSK‐3‐inhibitor‐II had similar chemical structures (Figure 12A,C). Both Entinostat^54^ and Diphencyprone^55^ have been widely used in immunotherapies for many other diseases. This further illustrates that the feature genes identified in this study are potential immunotherapeutic targets for the treatment of KC.

The IHC staining of normal and KC corneas further verified our data, with significantly higher expression of MYRF in KC corneas, contrary to the expression of ATOH7 (Figure 13). We have already discussed the important role of *MYRF* in the immune response and the structural development of KC. *ATOH7* is involved in the development of photoreceptors, retinal ganglion cells and optic nerves.^56^, ^57^ Mutations in *ATOH7* are associated with several retinal diseases, including autosomal recessive persistent hyperplastic primary vitreous^58^ and familial exudative vitreoretinopathies.^59^ Our findings regarding *ATOH7* in KC implied an important function of *ATOH7* in the normal development of the cornea. The opposite effects and expression tendencies of *MYRF* and *ATOH7* in KC also indicated the potential role of *ATOH7* in the immune regulation of KC progression. Considering the genetic role of *ATOH7* and *MYRF* in ocular tissues and combining with the enriched pathways in KC, the differential expression of *MYRF* and *ATOH7* in KC may affect the synthesis of compound in KC corneas. Therefore, KC may not only be related to the immune response but also to the abnormal development or synthesis of structures owing to genetic factors.

However, due to the shortage of primary antibody types, only the protein expression levels of MYRF and ATOH7 were verified by IHC. The lack of an accurate animal model for KC also limits our further exploration in molecular mechanism of KC as well as verification of the effect of predicted drugs on KC in vivo.^60^ Thus, the establishment of an effective, precise and stable animal model for KC is essential. More studies investigating the regulation of immune response and specific molecular mechanisms in KC need to be accomplished in the future.

The present research has reported notable findings, but it also has limitations. Although some significant results were obtained in this study, the limited sample size still restricted the breadth and depth of this research. In addition, the identified target genes were not prospectively validated, which might affect the reliability of the results. Future studies with more samples, larger cohorts and prospective validation are essential to be conducted in the future. Moreover, although GSK3B inhibitors have been identified as potential therapeutic targets, our study did not find the involvement of Wnt‐related genes, indicating that GSK3B may operate through alternative pathways, such as those related to cellular stress responses or fibrosis.^61^, ^62^ More researches into these mechanisms are also necessary.

In conclusion, we determined the feature genes of KC, constructed a diagnostic model, predicted therapeutic targets and found that the pathophysiology of KC was not only related to immune infiltration regulated by transcription factors and miRNAs correlated to feature genes but also abnormal development or synthesis of corneal structures caused by genetic factors.

## AUTHOR CONTRIBUTIONS

**Ning Lyu:** Conceptualization (equal); investigation (equal); writing – original draft (lead). **Yiqin Dai:** Formal analysis (equal); investigation (equal); methodology (equal). **Jiawen Wu:** Conceptualization (in revision); formal analysis (in revision); methodology (in revision); formal analysis (in revision); writing ‐ revised draft. **Yidan Fan:** Conceptualization (equal); formal analysis (equal); writing – original draft (equal). **Zhaoyuan Lyu:** Data curation (equal); methodology (equal); project administration (equal); writing – review and editing (equal). **Jiayu Gu:** Data curation (equal); formal analysis (equal); validation (equal); writing – review and editing (equal). **Jingyi Cheng:** Funding acquisition (equal); investigation (equal); writing – review and editing (equal). **Jianjiang Xu:** Conceptualization (equal); funding acquisition (equal); supervision (equal); writing – review and editing (equal).

## FUNDING INFORMATION

The authors were sponsored by the National Natural Science Foundation of China (grant nos. 82201147 and 82171020); The sponsor or funding organization had no role in the design or conduct of this research.

## CONFLICT OF INTEREST STATEMENT

None.

## Supporting information

Material S1.

Material S2.

Material S3.

Material S4.

Material S5.

## Data Availability

Data can be made available from the corresponding author upon request.

## References

[1] Li X , Rabinowitz YS , Rasheed K , Yang H . Longitudinal study of the normal eyes in unilateral keratoconus patients. Ophthalmology. 2004;111(3):440‐446. 15019316 10.1016/j.ophtha.2003.06.020

[2] Santodomingo‐Rubido J , Carracedo G , Suzaki A , Villa‐Collar C , Vincent SJ , Wolffsohn JS . Keratoconus: an updated review. Cont Lens Anterior Eye. 2022;45(3):101559. 34991971 10.1016/j.clae.2021.101559

[3] Soiberman U , Foster JW , Jun AS , Chakravarti S . Pathophysiology of keratoconus: what do we know today. Open Ophthalmol J. 2017;11:252‐261. 28932341 10.2174/1874364101711010252 PMC5585454

[4] Gomes JA , Tan D , Rapuano CJ , et al. Global consensus on keratoconus and ectatic diseases. Cornea. 2015;34(4):359‐369. 25738235 10.1097/ICO.0000000000000408

[5] Flockerzi E , Xanthopoulou K , Goebels SC , et al. Keratoconus staging by decades: a baseline ABCD classification of 1000 patients in the Homburg keratoconus center. Br J Ophthalmol. 2021;105(8):1069‐1075. 32830125 10.1136/bjophthalmol-2020-316789

[6] Hwang S , Lim DH , Chung TY . Prevalence and incidence of keratoconus in South Korea: a Nationwide population‐based study. Am J Ophthalmol. 2018;192:56‐64. 29750946 10.1016/j.ajo.2018.04.027

[7] Gao H , Huang T , Pan Z , et al. Survey report on keratoplasty in China: a 5‐year review from 2014 to 2018. PLoS One. 2020;15(10):e0239939. 33057425 10.1371/journal.pone.0239939 PMC7561196

[8] Park CY , Lee JK , Gore PK , Lim CY , Chuck RS . Keratoplasty in the United States: a 10‐year review from 2005 through 2014. Ophthalmology. 2015;122(12):2432‐2442. 26386848 10.1016/j.ophtha.2015.08.017

[9] Kosker M , Arslan N , Alp MY , et al. Association between keratoconus and familial Mediterranean fever in Turkey. Cornea. 2016;35(1):77‐80. 26509767 10.1097/ICO.0000000000000662

[10] Wang Y , Rabinowitz YS , Rotter JI , Yang H . Genetic epidemiological study of keratoconus: evidence for major gene determination. Am J Med Genet. 2000;93(5):403‐409. 10951465

[11] Zahid S , Branham K , Schlegel D , et al. SPATA7. In: Retinal Dystrophy Gene Atlas. Springer, Cham. 2018;257‐258.

[12] Rabinowitz YS , Dong L , Wistow G . Gene expression profile studies of human keratoconus cornea for NEIBank: a novel cornea‐expressed gene and the absence of transcripts for aquaporin 5. Invest Ophthalmol Vis Sci. 2005;46(4):1239‐1246. 15790884 10.1167/iovs.04-1148

[13] Mas Tur V , MacGregor C , Jayaswal R , O'Brart D , Maycock N . A review of keratoconus: diagnosis, pathophysiology, and genetics. Surv Ophthalmol. 2017;62(6):770‐783. 28688894 10.1016/j.survophthal.2017.06.009

[14] Bykhovskaya Y , Li X , Taylor KD , Haritunians T , Rotter JI , Rabinowitz YS . Linkage analysis of high‐density SNPs confirms keratoconus locus at 5q chromosomal region. Ophthalmic Genet. 2016;37(1):109‐110. 24555746 10.3109/13816810.2014.889172 PMC4139481

[15] Loh IP , Sherwin T . Is keratoconus an inflammatory disease? The implication of inflammatory pathways. Ocul Immunol Inflamm. 2022;30(1):246‐255. 32791016 10.1080/09273948.2020.1780271

[16] Elbeyli A , Kurtul BE . Systemic immune‐inflammation index, neutrophil‐to‐lymphocyte ratio, and platelet‐to‐lymphocyte ratio levels are associated with keratoconus. Indian J Ophthalmol. 2021;69(7):1725‐1729. 34146015 10.4103/ijo.IJO_3011_20 PMC8374788

[17] Wisse RP et al. Cytokine expression in keratoconus and its corneal microenvironment: a systematic review. Ocul Surf. 2015;13(4):272‐283. 26235733 10.1016/j.jtos.2015.04.006

[18] Hu D et al. Identification of key genes and molecular pathways in keratoconus: integrating text mining and bioinformatics analysis. Biomed Res Int. 2022;2022:4740141. 36051483 10.1155/2022/4740141 PMC9427295

[19] Hwang B , Lee JH , Bang D . Single‐cell RNA sequencing technologies and bioinformatics pipelines. Exp Mol Med. 2018;50(8):1‐14. 10.1038/s12276-018-0071-8 PMC6082860 30089861

[20] Li X , Wang CY . From bulk, single‐cell to spatial RNA sequencing. Int J Oral Sci. 2021;13(1):36. 34782601 10.1038/s41368-021-00146-0 PMC8593179

[21] Alpern D , Gardeux V , Russeil J , et al. BRB‐seq: ultra‐affordable high‐throughput transcriptomics enabled by bulk RNA barcoding and sequencing. Genome Biol. 2019;20(1):71. 30999927 10.1186/s13059-019-1671-x PMC6474054

[22] Kolodziejczyk AA , Kim JK , Svensson V , Marioni JC , Teichmann SA . The technology and biology of single‐cell RNA sequencing. Mol Cell. 2015;58(4):610‐620. 26000846 10.1016/j.molcel.2015.04.005

[23] Belin MW , Jang HS , Borgstrom M . Keratoconus: diagnosis and staging. Cornea. 2022;41(1):1‐11. 34116536 10.1097/ICO.0000000000002781

[24] Jensen EC . Quantitative analysis of histological staining and fluorescence using ImageJ. Anat Rec (Hoboken). 2013;296(3):378‐381. 23382140 10.1002/ar.22641

[25] De‐Yong Z . Advances in research of genes involved in anthocyanin biological synthesis in plant and the genetic modification of the pathway. Journal of Tropical Organisms. 2012;1(1):92‐98.

[26] Haggard DE , Das SR , Tanguay RL . Comparative toxicogenomic responses to the flame retardant mITP in developing zebrafish. Chem Res Toxicol. 2017;30(2):508‐515. 27957850 10.1021/acs.chemrestox.6b00423

[27] Volkers G , Palm GJ , Weiss MS , Wright GD , Hinrichs W . Structural basis for a new tetracycline resistance mechanism relying on the TetX monooxygenase. FEBS Lett. 2011;585(7):1061‐1066. 21402075 10.1016/j.febslet.2011.03.012

[28] Qazi Y , Turhan A , Hamrah P . Trafficking of immune cells in the cornea and ocular surface. Adv Ophthalmol. 2012;5:79‐104.

[29] Raphael I , Joern RR , Forsthuber TG . Memory CD4+ T cells in immunity and autoimmune diseases. Cells. 2020;9(3):531. 32106536 10.3390/cells9030531 PMC7140455

[30] Malkiel S , Barlev AN , Atisha‐Fregoso Y , Suurmond J , Diamond B . Plasma cell differentiation pathways in systemic lupus erythematosus. Front Immunol. 2018;9:427. 29556239 10.3389/fimmu.2018.00427 PMC5845388

[31] Dyck L , Mills KHG . Immune checkpoints and their inhibition in cancer and infectious diseases. Eur J Immunol. 2017;47(5):765‐779. 28393361 10.1002/eji.201646875

[32] Sica GL , Choi IH , Zhu G , et al. B7‐H4, a molecule of the B7 family, negatively regulates T cell immunity. Immunity. 2003;18(6):849‐861. 12818165 10.1016/s1074-7613(03)00152-3

[33] McMonnies CW . Inflammation and keratoconus. Optom Vis Sci. 2015;92(2):e35‐e41. 25397925 10.1097/OPX.0000000000000455

[34] Chen X , Liu C , Cui Z , et al. Integrative transcriptomics analysis and experimental validation reveal immunomodulatory patterns in keratoconus. Exp Eye Res. 2023;230:109460. 37001853 10.1016/j.exer.2023.109460

[35] D'Souza S et al. Keratoconus patients exhibit a distinct ocular surface immune cell and inflammatory profile. Sci Rep. 2021;11(1):20891. 34686755 10.1038/s41598-021-99805-9 PMC8536707

[36] Claessens JLJ , Godefrooij DA , Vink G , Frank LE , Wisse RPL . Nationwide epidemiological approach to identify associations between keratoconus and immune‐mediated diseases. Br J Ophthalmol. 2022;106(10):1350‐1354. 33879468 10.1136/bjophthalmol-2021-318804 PMC9510397

[37] Arthur JS , Ley SC . Mitogen‐activated protein kinases in innate immunity. Nat Rev Immunol. 2013;13(9):679‐692. 23954936 10.1038/nri3495

[38] Wen Y , Jing Y , Yang L , et al. The regulators of BCR signaling during B cell activation. Blood Sci. 2019;1(2):119‐129. 35402811 10.1097/BS9.0000000000000026 PMC8975005

[39] Nam S , Lim JS . Essential role of interferon regulatory factor 4 (IRF4) in immune cell development. Arch Pharm Res. 2016;39(11):1548‐1555. 27826752 10.1007/s12272-016-0854-1

[40] Krausgruber T , Blazek K , Smallie T , et al. IRF5 promotes inflammatory macrophage polarization and TH1‐TH17 responses. Nat Immunol. 2011;12(3):231‐238. 21240265 10.1038/ni.1990

[41] Kaplan MH . STAT4: a critical regulator of inflammation in vivo. Immunol Res. 2005;31(3):231‐242. 15888914 10.1385/IR:31:3:231

[42] Yang XO , Panopoulos AD , Nurieva R , et al. STAT3 regulates cytokine‐mediated generation of inflammatory helper T cells. J Biol Chem. 2007;282(13):9358‐9363. 17277312 10.1074/jbc.C600321200

[43] Tkach M , Rosemblit C , Rivas MA , et al. p42/p44 MAPK‐mediated Stat3Ser727 phosphorylation is required for progestin‐induced full activation of Stat3 and breast cancer growth. Endocr Relat Cancer. 2013;20(2):197‐212. 23329648 10.1530/ERC-12-0194

[44] Liping M et al. Expression and significance of transcription factor EBF3 in tissues of human primary hepatocellular cancer. Chinese Journal of Clinical Laboratory Science. 2008;20(1):170‐181.

[45] Milde‐Langosch K . The Fos family of transcription factors and their role in tumourigenesis. Eur J Cancer. 2005;41(16):2449‐2461. 16199154 10.1016/j.ejca.2005.08.008

[46] Hermann‐Kleiter N , Meisel M , Fresser F , et al. Nuclear orphan receptor NR2F6 directly antagonizes NFAT and RORγt binding to the Il17a promoter. J Autoimmun. 2012;39(4):428‐440. 22921335 10.1016/j.jaut.2012.07.007 PMC3516707

[47] Emery B , Agalliu D , Cahoy JD , et al. Myelin gene regulatory factor is a critical transcriptional regulator required for CNS myelination. Cell. 2009;138(1):172‐185. 19596243 10.1016/j.cell.2009.04.031 PMC2757090

[48] Huang H , Zhou F , Zhou S , Qiu M . MYRF: a mysterious membrane‐bound transcription factor involved in myelin development and human diseases. Neurosci Bull. 2021;37(6):881‐884. 33864620 10.1007/s12264-021-00678-9 PMC8192642

[49] Garnai SJ , Brinkmeier ML , Emery B , et al. Variants in myelin regulatory factor (MYRF) cause autosomal dominant and syndromic nanophthalmos in humans and retinal degeneration in mice. PLoS Genet. 2019;15(5):e1008130. 31048900 10.1371/journal.pgen.1008130 PMC6527243

[50] Shi L , Zhang Y , Xia Y , Li C , Song Z , Zhu J . MiR‐150‐5p protects against septic acute kidney injury via repressing the MEKK3/JNK pathway. Cell Signal. 2021;86:110101. 34333083 10.1016/j.cellsig.2021.110101

[51] Oshi M , Maiti A , Wu R , et al. miR‐150 expression in breast cancer attracts and activates immune cells, and is associated with better patient outcome. J Clin Oncol. 2022;40(16_suppl):570.

[52] Ishihara S , Sato M , Miyazaki H , et al. Deletion of miR‐150 prevents spontaneous T cell proliferation and the development of colitis. Gastro Hep Advances. 2023;2(4):487‐496. 39132043 10.1016/j.gastha.2023.01.021 PMC11308117

[53] Kalaimani L et al. Hsa‐miR‐150‐5p inhibits Wnt‐β‐catenin signaling in human corneal epithelial stem cells. Mol Vis. 2022;28:178‐191. 36274818 PMC9491245

[54] Peng‐Kun LI et al. A Histone Deacetylase Inhibitor:Entinostat. Drugs & Clinic; 2023.

[55] Aljuffali IA , Sung CT , Shen FM , Huang CT , Fang JY . Squarticles as a lipid nanocarrier for delivering diphencyprone and minoxidil to hair follicles and human dermal papilla cells. AAPS J. 2014;16(1):140‐150. 24307611 10.1208/s12248-013-9550-y PMC3889522

[56] Brown NL , Dagenais SL , Chen CM , Glaser T . Molecular characterization and mapping of ATOH7, a human atonal homolog with a predicted role in retinal ganglion cell development. Mamm Genome. 2002;13(2):95‐101. 11889557 10.1007/s00335-001-2101-3 PMC2262845

[57] Brown NL , Patel S , Brzezinski J , Glaser T . Math5 is required for retinal ganglion cell and optic nerve formation. Development. 2001;128(13):2497‐2508. 11493566 10.1242/dev.128.13.2497 PMC1480839

[58] Prasov L , Masud T , Khaliq S , et al. ATOH7 mutations cause autosomal recessive persistent hyperplasia of the primary vitreous. Hum Mol Genet. 2012;21(16):3681‐3694. 22645276 10.1093/hmg/dds197 PMC3406761

[59] Naruse S , Kondo H . Ocular features associated with mutations in atoh7 gene overlap those with familial exudative vitreoretinopathy. Retin Cases Brief Rep. 2023;17(6):694‐698. 35389970 10.1097/ICB.0000000000001267

[60] Hadvina R , Estes A , Liu Y . Animal models for the study of keratoconus. Cells. 2023;12(23):2681. 38067109 10.3390/cells12232681 PMC10705680

[61] Kourtis N , Moubarak RS , Aranda‐Orgilles B , et al. FBXW7 modulates cellular stress response and metastatic potential through HSF1 post‐translational modification. Nat Cell Biol. 2015;17(3):322‐332. 25720964 10.1038/ncb3121 PMC4401662

[62] Sun AB , Li FH , Zhu L , et al. TRPC6 knockout alleviates renal fibrosis through PI3K/AKT/GSK3B pathway. Curr Med Sci. 2024;44(3):589‐602. 38748370 10.1007/s11596-024-2869-z

