# Supplementary material for: Multi‐dataset identification of innovative feature genes and molecular mechanisms in keratoconus
Source: J Cell Mol Med. 2024 Sep 19;28(18):e70079. doi: 10.1111/jcmm.70079 (PMC11412914; doi:10.1111/jcmm.70079)
Supplement: Supplementary file 2 — Material S2. [file JCMM-28-e70079-s002.zip › 7.GSEA analysis/1.ARL11/gsea_report_for_h_1678852737554.html]

Report for h 1678852737554 [GSEA]

| GS  follow link to MSigDB | GS DETAILS | SIZE | ES | NES | NOM p-val | FDR q-val | FWER p-val | RANK AT MAX | LEADING EDGE || 1 | KEGG\_PORPHYRIN\_AND\_CHLOROPHYLL\_METABOLISM | Details ... | 32 | 0.53 | 1.73 | 0.006 | 0.315 | 0.303 | 5761 | tags=31%, list=17%, signal=37% |
| 2 | KEGG\_GLYCOSPHINGOLIPID\_BIOSYNTHESIS\_GANGLIO\_SERIES | Details ... | 15 | 0.56 | 1.53 | 0.064 | 1.000 | 0.733 | 11491 | tags=60%, list=33%, signal=90% |
| 3 | KEGG\_ASCORBATE\_AND\_ALDARATE\_METABOLISM | Details ... | 17 | 0.56 | 1.51 | 0.016 | 0.782 | 0.774 | 5683 | tags=41%, list=16%, signal=49% |
| 4 | KEGG\_STARCH\_AND\_SUCROSE\_METABOLISM | Details ... | 39 | 0.47 | 1.44 | 0.083 | 0.969 | 0.848 | 5683 | tags=28%, list=16%, signal=34% |
| 5 | KEGG\_GLYCINE\_SERINE\_AND\_THREONINE\_METABOLISM | Details ... | 31 | 0.35 | 1.18 | 0.190 | 1.000 | 0.993 | 4162 | tags=23%, list=12%, signal=26% |
| 6 | KEGG\_GLYOXYLATE\_AND\_DICARBOXYLATE\_METABOLISM | Details ... | 16 | 0.41 | 1.17 | 0.292 | 1.000 | 0.993 | 7381 | tags=19%, list=21%, signal=24% |
| 7 | KEGG\_BASE\_EXCISION\_REPAIR | Details ... | 33 | 0.36 | 1.15 | 0.355 | 1.000 | 0.995 | 19 | tags=3%, list=0%, signal=3% |
| 8 | KEGG\_PENTOSE\_AND\_GLUCURONATE\_INTERCONVERSIONS | Details ... | 20 | 0.43 | 1.14 | 0.290 | 1.000 | 0.995 | 5683 | tags=35%, list=16%, signal=42% |
| 9 | KEGG\_PRIMARY\_IMMUNODEFICIENCY | Details ... | 35 | 0.40 | 1.11 | 0.321 | 1.000 | 0.996 | 5041 | tags=23%, list=15%, signal=27% |
| 10 | KEGG\_NUCLEOTIDE\_EXCISION\_REPAIR | Details ... | 43 | 0.25 | 1.08 | 0.335 | 1.000 | 0.998 | 26059 | tags=100%, list=75%, signal=400% |
| 11 | KEGG\_PHOSPHATIDYLINOSITOL\_SIGNALING\_SYSTEM | Details ... | 76 | 0.24 | 1.04 | 0.421 | 1.000 | 0.998 | 10781 | tags=41%, list=31%, signal=59% |
| 12 | KEGG\_PRIMARY\_BILE\_ACID\_BIOSYNTHESIS | Details ... | 16 | 0.44 | 1.02 | 0.423 | 1.000 | 0.999 | 4496 | tags=25%, list=13%, signal=29% |
| 13 | KEGG\_RNA\_DEGRADATION | Details ... | 51 | 0.19 | 0.99 | 0.514 | 1.000 | 1.000 | 28116 | tags=100%, list=81%, signal=524% |
| 14 | KEGG\_PHENYLALANINE\_METABOLISM | Details ... | 17 | 0.36 | 0.99 | 0.494 | 1.000 | 1.000 | 4200 | tags=18%, list=12%, signal=20% |
| 15 | KEGG\_HOMOLOGOUS\_RECOMBINATION | Details ... | 26 | 0.30 | 0.98 | 0.447 | 1.000 | 1.000 | 11636 | tags=42%, list=34%, signal=64% |
| 16 | KEGG\_GALACTOSE\_METABOLISM | Details ... | 25 | 0.32 | 0.97 | 0.500 | 1.000 | 1.000 | 2679 | tags=12%, list=8%, signal=13% |
| 17 | KEGG\_TASTE\_TRANSDUCTION | Details ... | 52 | 0.33 | 0.97 | 0.521 | 1.000 | 1.000 | 6815 | tags=35%, list=20%, signal=43% |
| 18 | KEGG\_CARDIAC\_MUSCLE\_CONTRACTION | Details ... | 72 | 0.26 | 0.96 | 0.556 | 1.000 | 1.000 | 2566 | tags=8%, list=7%, signal=9% |
| 19 | KEGG\_NEUROACTIVE\_LIGAND\_RECEPTOR\_INTERACTION | Details ... | 270 | 0.26 | 0.96 | 0.587 | 1.000 | 1.000 | 4939 | tags=21%, list=14%, signal=24% |
| 20 | KEGG\_VIBRIO\_CHOLERAE\_INFECTION | Details ... | 51 | 0.23 | 0.96 | 0.532 | 1.000 | 1.000 | 6821 | tags=18%, list=20%, signal=22% |
| 21 | KEGG\_BETA\_ALANINE\_METABOLISM | Details ... | 22 | 0.34 | 0.96 | 0.506 | 1.000 | 1.000 | 4971 | tags=23%, list=14%, signal=27% |
| 22 | KEGG\_GLYCEROLIPID\_METABOLISM | Details ... | 43 | 0.27 | 0.95 | 0.501 | 1.000 | 1.000 | 8228 | tags=40%, list=24%, signal=52% |
| 23 | KEGG\_PEROXISOME | Details ... | 78 | 0.24 | 0.95 | 0.551 | 1.000 | 1.000 | 9236 | tags=24%, list=27%, signal=33% |
| 24 | KEGG\_BIOSYNTHESIS\_OF\_UNSATURATED\_FATTY\_ACIDS | Details ... | 19 | 0.27 | 0.94 | 0.561 | 1.000 | 1.000 | 9236 | tags=26%, list=27%, signal=36% |
| 25 | KEGG\_ALANINE\_ASPARTATE\_AND\_GLUTAMATE\_METABOLISM | Details ... | 30 | 0.28 | 0.93 | 0.566 | 1.000 | 1.000 | 4200 | tags=13%, list=12%, signal=15% |
| 26 | KEGG\_HISTIDINE\_METABOLISM | Details ... | 28 | 0.34 | 0.92 | 0.563 | 1.000 | 1.000 | 1064 | tags=11%, list=3%, signal=11% |
| 27 | KEGG\_GLYCOSAMINOGLYCAN\_BIOSYNTHESIS\_HEPARAN\_SULFATE | Details ... | 26 | 0.28 | 0.91 | 0.584 | 1.000 | 1.000 | 7829 | tags=23%, list=23%, signal=30% |
| 28 | KEGG\_MISMATCH\_REPAIR | Details ... | 23 | 0.26 | 0.91 | 0.580 | 1.000 | 1.000 | 25828 | tags=100%, list=74%, signal=390% |
| 29 | KEGG\_STEROID\_BIOSYNTHESIS | Details ... | 17 | 0.30 | 0.91 | 0.589 | 1.000 | 1.000 | 5826 | tags=18%, list=17%, signal=21% |
| 30 | KEGG\_ABC\_TRANSPORTERS | Details ... | 44 | 0.30 | 0.91 | 0.557 | 1.000 | 1.000 | 3637 | tags=18%, list=10%, signal=20% |
| 31 | KEGG\_ANTIGEN\_PROCESSING\_AND\_PRESENTATION | Details ... | 79 | 0.27 | 0.89 | 0.606 | 1.000 | 1.000 | 9665 | tags=33%, list=28%, signal=45% |
| 32 | KEGG\_DRUG\_METABOLISM\_CYTOCHROME\_P450 | Details ... | 62 | 0.27 | 0.89 | 0.677 | 1.000 | 1.000 | 6053 | tags=26%, list=17%, signal=31% |
| 33 | KEGG\_NON\_SMALL\_CELL\_LUNG\_CANCER | Details ... | 54 | 0.19 | 0.88 | 0.612 | 1.000 | 1.000 | 10673 | tags=33%, list=31%, signal=48% |
| 34 | KEGG\_RETINOL\_METABOLISM | Details ... | 55 | 0.27 | 0.87 | 0.734 | 1.000 | 1.000 | 6286 | tags=27%, list=18%, signal=33% |
| 35 | KEGG\_HEMATOPOIETIC\_CELL\_LINEAGE | Details ... | 87 | 0.28 | 0.86 | 0.677 | 1.000 | 1.000 | 3809 | tags=18%, list=11%, signal=21% |
| 36 | KEGG\_COLORECTAL\_CANCER | Details ... | 62 | 0.21 | 0.86 | 0.634 | 1.000 | 1.000 | 11167 | tags=39%, list=32%, signal=57% |
| 37 | KEGG\_LEUKOCYTE\_TRANSENDOTHELIAL\_MIGRATION | Details ... | 114 | 0.23 | 0.85 | 0.771 | 1.000 | 1.000 | 7118 | tags=25%, list=20%, signal=31% |
| 38 | KEGG\_GLYCOSAMINOGLYCAN\_DEGRADATION | Details ... | 21 | 0.27 | 0.85 | 0.632 | 1.000 | 1.000 | 211 | tags=5%, list=1%, signal=5% |
| 39 | KEGG\_GNRH\_SIGNALING\_PATHWAY | Details ... | 100 | 0.18 | 0.84 | 0.765 | 1.000 | 1.000 | 6310 | tags=14%, list=18%, signal=17% |
| 40 | KEGG\_SMALL\_CELL\_LUNG\_CANCER | Details ... | 84 | 0.21 | 0.84 | 0.652 | 1.000 | 1.000 | 2870 | tags=8%, list=8%, signal=9% |
| 41 | KEGG\_GAP\_JUNCTION | Details ... | 86 | 0.18 | 0.83 | 0.873 | 1.000 | 1.000 | 7891 | tags=23%, list=23%, signal=30% |
| 42 | KEGG\_UBIQUITIN\_MEDIATED\_PROTEOLYSIS | Details ... | 130 | 0.14 | 0.83 | 0.671 | 1.000 | 1.000 | 26643 | tags=98%, list=77%, signal=421% |
| 43 | KEGG\_PROTEIN\_EXPORT | Details ... | 22 | 0.25 | 0.81 | 0.818 | 1.000 | 1.000 | 2129 | tags=5%, list=6%, signal=5% |
| 44 | KEGG\_HYPERTROPHIC\_CARDIOMYOPATHY\_HCM | Details ... | 83 | 0.24 | 0.81 | 0.787 | 1.000 | 1.000 | 1554 | tags=7%, list=4%, signal=8% |
| 45 | KEGG\_CYSTEINE\_AND\_METHIONINE\_METABOLISM | Details ... | 34 | 0.18 | 0.80 | 0.767 | 1.000 | 1.000 | 6578 | tags=15%, list=19%, signal=18% |
| 46 | KEGG\_INSULIN\_SIGNALING\_PATHWAY | Details ... | 136 | 0.18 | 0.80 | 0.683 | 1.000 | 1.000 | 10691 | tags=32%, list=31%, signal=46% |
| 47 | KEGG\_PANCREATIC\_CANCER | Details ... | 69 | 0.19 | 0.79 | 0.725 | 1.000 | 1.000 | 11167 | tags=32%, list=32%, signal=47% |
| 48 | KEGG\_PANTOTHENATE\_AND\_COA\_BIOSYNTHESIS | Details ... | 16 | 0.30 | 0.79 | 0.771 | 1.000 | 1.000 | 4971 | tags=19%, list=14%, signal=22% |
| 49 | KEGG\_PPAR\_SIGNALING\_PATHWAY | Details ... | 69 | 0.24 | 0.78 | 0.821 | 1.000 | 1.000 | 2836 | tags=12%, list=8%, signal=13% |
| 50 | KEGG\_SPLICEOSOME | Details ... | 125 | 0.18 | 0.78 | 0.763 | 1.000 | 1.000 | 25995 | tags=98%, list=75%, signal=390% |
| 51 | KEGG\_TERPENOID\_BACKBONE\_BIOSYNTHESIS |  | 15 | 0.30 | 0.78 | 0.699 | 1.000 | 1.000 | 24327 | tags=100%, list=70%, signal=334% |
| 52 | KEGG\_DILATED\_CARDIOMYOPATHY |  | 90 | 0.21 | 0.77 | 0.928 | 1.000 | 1.000 | 1554 | tags=7%, list=4%, signal=7% |
| 53 | KEGG\_ARGININE\_AND\_PROLINE\_METABOLISM |  | 52 | 0.22 | 0.76 | 0.786 | 1.000 | 1.000 | 2884 | tags=10%, list=8%, signal=10% |
| 54 | KEGG\_PROGESTERONE\_MEDIATED\_OOCYTE\_MATURATION |  | 85 | 0.18 | 0.75 | 0.800 | 1.000 | 1.000 | 11276 | tags=35%, list=32%, signal=52% |
| 55 | KEGG\_OOCYTE\_MEIOSIS |  | 111 | 0.15 | 0.74 | 0.853 | 1.000 | 1.000 | 11259 | tags=28%, list=32%, signal=41% |
| 56 | KEGG\_APOPTOSIS |  | 87 | 0.18 | 0.73 | 0.845 | 1.000 | 1.000 | 6150 | tags=14%, list=18%, signal=17% |
| 57 | KEGG\_B\_CELL\_RECEPTOR\_SIGNALING\_PATHWAY |  | 75 | 0.18 | 0.73 | 0.901 | 1.000 | 1.000 | 6424 | tags=19%, list=18%, signal=23% |
| 58 | KEGG\_NITROGEN\_METABOLISM |  | 23 | 0.28 | 0.72 | 0.875 | 1.000 | 1.000 | 777 | tags=9%, list=2%, signal=9% |
| 59 | KEGG\_T\_CELL\_RECEPTOR\_SIGNALING\_PATHWAY |  | 107 | 0.19 | 0.70 | 0.910 | 1.000 | 1.000 | 6194 | tags=17%, list=18%, signal=20% |
| 60 | KEGG\_COMPLEMENT\_AND\_COAGULATION\_CASCADES |  | 69 | 0.22 | 0.70 | 0.972 | 1.000 | 1.000 | 3662 | tags=14%, list=11%, signal=16% |
| 61 | KEGG\_P53\_SIGNALING\_PATHWAY |  | 66 | 0.17 | 0.63 | 0.888 | 1.000 | 1.000 | 9607 | tags=24%, list=28%, signal=33% |
| 62 | KEGG\_INOSITOL\_PHOSPHATE\_METABOLISM |  | 54 | 0.16 | 0.63 | 0.866 | 1.000 | 1.000 | 5192 | tags=11%, list=15%, signal=13% |
| 63 | KEGG\_TYPE\_II\_DIABETES\_MELLITUS |  | 47 | 0.20 | 0.62 | 0.963 | 1.000 | 1.000 | 6079 | tags=19%, list=18%, signal=23% |
| 64 | KEGG\_VASOPRESSIN\_REGULATED\_WATER\_REABSORPTION |  | 44 | 0.16 | 0.58 | 0.944 | 1.000 | 1.000 | 5439 | tags=11%, list=16%, signal=13% |
| 65 | KEGG\_TYPE\_I\_DIABETES\_MELLITUS |  | 43 | 0.22 | 0.58 | 0.970 | 1.000 | 1.000 | 8445 | tags=33%, list=24%, signal=43% |
| 66 | KEGG\_GLYCOSPHINGOLIPID\_BIOSYNTHESIS\_LACTO\_AND\_NEOLACTO\_SERIES |  | 26 | 0.19 | 0.56 | 0.975 | 1.000 | 1.000 | 4610 | tags=15%, list=13%, signal=18% |
| 67 | KEGG\_ENDOMETRIAL\_CANCER |  | 52 | 0.14 | 0.54 | 0.965 | 1.000 | 1.000 | 11115 | tags=33%, list=32%, signal=48% |
| 68 | KEGG\_SELENOAMINO\_ACID\_METABOLISM |  | 24 | 0.17 | 0.52 | 0.965 | 1.000 | 1.000 | 28775 | tags=100%, list=83%, signal=583% |
| 69 | KEGG\_VALINE\_LEUCINE\_AND\_ISOLEUCINE\_DEGRADATION |  | 43 | 0.14 | 0.50 | 0.981 | 1.000 | 1.000 | 11858 | tags=33%, list=34%, signal=49% |
| 70 | KEGG\_ONE\_CARBON\_POOL\_BY\_FOLATE |  | 17 | 0.17 | 0.48 | 0.980 | 1.000 | 1.000 | 12150 | tags=35%, list=35%, signal=54% |
| 71 | KEGG\_MTOR\_SIGNALING\_PATHWAY |  | 51 | 0.14 | 0.47 | 0.990 | 0.993 | 1.000 | 11351 | tags=37%, list=33%, signal=55% |
Table: Gene sets enriched in phenotype **h (8 samples)**[plain text format]****

  
