# Supplementary material for: Multi‐dataset identification of innovative feature genes and molecular mechanisms in keratoconus
Source: J Cell Mol Med. 2024 Sep 19;28(18):e70079. doi: 10.1111/jcmm.70079 (PMC11412914; doi:10.1111/jcmm.70079)
Supplement: Supplementary file 2 — Material S2. [file JCMM-28-e70079-s002.zip › 7.GSEA analysis/1.ARL11/gsea_report_for_l_1678852737554.html]

Report for l 1678852737554 [GSEA]

| GS  follow link to MSigDB | GS DETAILS | SIZE | ES | NES | NOM p-val | FDR q-val | FWER p-val | RANK AT MAX | LEADING EDGE || 1 | KEGG\_RENIN\_ANGIOTENSIN\_SYSTEM | Details ... | 17 | -0.64 | -1.50 | 0.058 | 1.000 | 0.774 | 8585 | tags=47%, list=25%, signal=62% |
| 2 | KEGG\_GLYCOSAMINOGLYCAN\_BIOSYNTHESIS\_CHONDROITIN\_SULFATE | Details ... | 22 | -0.54 | -1.43 | 0.089 | 1.000 | 0.856 | 10965 | tags=45%, list=32%, signal=66% |
| 3 | KEGG\_OTHER\_GLYCAN\_DEGRADATION | Details ... | 15 | -0.44 | -1.28 | 0.221 | 1.000 | 0.963 | 537 | tags=7%, list=2%, signal=7% |
| 4 | KEGG\_REGULATION\_OF\_AUTOPHAGY | Details ... | 30 | -0.42 | -1.27 | 0.173 | 1.000 | 0.968 | 3175 | tags=17%, list=9%, signal=18% |
| 5 | KEGG\_LONG\_TERM\_DEPRESSION | Details ... | 67 | -0.37 | -1.25 | 0.168 | 1.000 | 0.973 | 8817 | tags=34%, list=25%, signal=46% |
| 6 | KEGG\_BASAL\_CELL\_CARCINOMA | Details ... | 55 | -0.42 | -1.25 | 0.191 | 1.000 | 0.973 | 8024 | tags=33%, list=23%, signal=42% |
| 7 | KEGG\_AUTOIMMUNE\_THYROID\_DISEASE | Details ... | 48 | -0.45 | -1.23 | 0.171 | 1.000 | 0.980 | 3964 | tags=25%, list=11%, signal=28% |
| 8 | KEGG\_ETHER\_LIPID\_METABOLISM | Details ... | 29 | -0.47 | -1.22 | 0.220 | 1.000 | 0.982 | 6891 | tags=31%, list=20%, signal=39% |
| 9 | KEGG\_ADHERENS\_JUNCTION | Details ... | 68 | -0.30 | -1.21 | 0.303 | 1.000 | 0.982 | 11246 | tags=31%, list=32%, signal=46% |
| 10 | KEGG\_OXIDATIVE\_PHOSPHORYLATION | Details ... | 98 | -0.28 | -1.19 | 0.275 | 1.000 | 0.988 | 15714 | tags=42%, list=45%, signal=76% |
| 11 | KEGG\_ARRHYTHMOGENIC\_RIGHT\_VENTRICULAR\_CARDIOMYOPATHY\_ARVC | Details ... | 74 | -0.38 | -1.18 | 0.251 | 1.000 | 0.988 | 8680 | tags=36%, list=25%, signal=49% |
| 12 | KEGG\_TGF\_BETA\_SIGNALING\_PATHWAY | Details ... | 85 | -0.35 | -1.17 | 0.268 | 1.000 | 0.989 | 11067 | tags=41%, list=32%, signal=60% |
| 13 | KEGG\_HUNTINGTONS\_DISEASE | Details ... | 158 | -0.21 | -1.16 | 0.269 | 1.000 | 0.990 | 7571 | tags=13%, list=22%, signal=16% |
| 14 | KEGG\_SNARE\_INTERACTIONS\_IN\_VESICULAR\_TRANSPORT | Details ... | 38 | -0.33 | -1.15 | 0.302 | 1.000 | 0.991 | 13980 | tags=47%, list=40%, signal=79% |
| 15 | KEGG\_OLFACTORY\_TRANSDUCTION | Details ... | 372 | -0.34 | -1.14 | 0.128 | 1.000 | 0.992 | 6562 | tags=33%, list=19%, signal=41% |
| 16 | KEGG\_BASAL\_TRANSCRIPTION\_FACTORS | Details ... | 33 | -0.27 | -1.14 | 0.313 | 1.000 | 0.993 | 14586 | tags=52%, list=42%, signal=89% |
| 17 | KEGG\_METABOLISM\_OF\_XENOBIOTICS\_BY\_CYTOCHROME\_P450 | Details ... | 60 | -0.35 | -1.12 | 0.281 | 1.000 | 0.994 | 6355 | tags=27%, list=18%, signal=33% |
| 18 | KEGG\_ALLOGRAFT\_REJECTION | Details ... | 37 | -0.46 | -1.11 | 0.353 | 1.000 | 0.996 | 3964 | tags=24%, list=11%, signal=27% |
| 19 | KEGG\_RIG\_I\_LIKE\_RECEPTOR\_SIGNALING\_PATHWAY | Details ... | 64 | -0.30 | -1.11 | 0.327 | 1.000 | 0.997 | 7963 | tags=22%, list=23%, signal=28% |
| 20 | KEGG\_LINOLEIC\_ACID\_METABOLISM | Details ... | 28 | -0.41 | -1.09 | 0.335 | 1.000 | 0.997 | 4107 | tags=21%, list=12%, signal=24% |
| 21 | KEGG\_TIGHT\_JUNCTION | Details ... | 127 | -0.29 | -1.09 | 0.324 | 1.000 | 0.997 | 4990 | tags=14%, list=14%, signal=16% |
| 22 | KEGG\_BLADDER\_CANCER | Details ... | 41 | -0.38 | -1.08 | 0.424 | 1.000 | 0.998 | 10732 | tags=32%, list=31%, signal=46% |
| 23 | KEGG\_ALPHA\_LINOLENIC\_ACID\_METABOLISM | Details ... | 18 | -0.45 | -1.07 | 0.371 | 1.000 | 0.998 | 4107 | tags=22%, list=12%, signal=25% |
| 24 | KEGG\_FC\_EPSILON\_RI\_SIGNALING\_PATHWAY | Details ... | 78 | -0.30 | -1.07 | 0.382 | 1.000 | 0.998 | 4600 | tags=17%, list=13%, signal=19% |
| 25 | KEGG\_MELANOGENESIS | Details ... | 101 | -0.28 | -1.07 | 0.356 | 1.000 | 0.998 | 9010 | tags=27%, list=26%, signal=36% |
| 26 | KEGG\_VASCULAR\_SMOOTH\_MUSCLE\_CONTRACTION | Details ... | 111 | -0.30 | -1.06 | 0.360 | 1.000 | 0.998 | 4107 | tags=15%, list=12%, signal=17% |
| 27 | KEGG\_MAPK\_SIGNALING\_PATHWAY | Details ... | 265 | -0.25 | -1.06 | 0.345 | 1.000 | 0.998 | 9437 | tags=28%, list=27%, signal=38% |
| 28 | KEGG\_PATHWAYS\_IN\_CANCER | Details ... | 322 | -0.24 | -1.06 | 0.375 | 1.000 | 0.998 | 9027 | tags=24%, list=26%, signal=32% |
| 29 | KEGG\_HEDGEHOG\_SIGNALING\_PATHWAY | Details ... | 55 | -0.33 | -1.05 | 0.394 | 1.000 | 0.998 | 8024 | tags=25%, list=23%, signal=33% |
| 30 | KEGG\_TYROSINE\_METABOLISM | Details ... | 40 | -0.32 | -1.04 | 0.425 | 1.000 | 0.999 | 2004 | tags=13%, list=6%, signal=13% |
| 31 | KEGG\_PYRUVATE\_METABOLISM | Details ... | 40 | -0.26 | -1.03 | 0.438 | 1.000 | 0.999 | 4258 | tags=13%, list=12%, signal=14% |
| 32 | KEGG\_GLYCOLYSIS\_GLUCONEOGENESIS | Details ... | 61 | -0.28 | -1.02 | 0.428 | 1.000 | 0.999 | 4461 | tags=13%, list=13%, signal=15% |
| 33 | KEGG\_CYTOKINE\_CYTOKINE\_RECEPTOR\_INTERACTION | Details ... | 256 | -0.34 | -1.01 | 0.458 | 1.000 | 1.000 | 7253 | tags=34%, list=21%, signal=42% |
| 34 | KEGG\_GLUTATHIONE\_METABOLISM | Details ... | 49 | -0.30 | -1.00 | 0.450 | 1.000 | 1.000 | 13160 | tags=51%, list=38%, signal=82% |
| 35 | KEGG\_BUTANOATE\_METABOLISM | Details ... | 34 | -0.34 | -1.00 | 0.474 | 1.000 | 1.000 | 5340 | tags=18%, list=15%, signal=21% |
| 36 | KEGG\_JAK\_STAT\_SIGNALING\_PATHWAY | Details ... | 151 | -0.30 | -1.00 | 0.489 | 1.000 | 1.000 | 7253 | tags=28%, list=21%, signal=36% |
| 37 | KEGG\_NEUROTROPHIN\_SIGNALING\_PATHWAY | Details ... | 125 | -0.20 | -1.00 | 0.437 | 1.000 | 1.000 | 11759 | tags=34%, list=34%, signal=51% |
| 38 | KEGG\_THYROID\_CANCER | Details ... | 29 | -0.29 | -0.99 | 0.478 | 1.000 | 1.000 | 12462 | tags=41%, list=36%, signal=64% |
| 39 | KEGG\_PROSTATE\_CANCER | Details ... | 89 | -0.23 | -0.99 | 0.483 | 1.000 | 1.000 | 10912 | tags=31%, list=31%, signal=46% |
| 40 | KEGG\_LYSINE\_DEGRADATION | Details ... | 39 | -0.29 | -0.96 | 0.514 | 1.000 | 1.000 | 10111 | tags=28%, list=29%, signal=40% |
| 41 | KEGG\_ALDOSTERONE\_REGULATED\_SODIUM\_REABSORPTION | Details ... | 42 | -0.29 | -0.96 | 0.485 | 1.000 | 1.000 | 5356 | tags=17%, list=15%, signal=20% |
| 42 | KEGG\_GLYCOSAMINOGLYCAN\_BIOSYNTHESIS\_KERATAN\_SULFATE | Details ... | 15 | -0.33 | -0.95 | 0.523 | 1.000 | 1.000 | 9293 | tags=47%, list=27%, signal=64% |
| 43 | KEGG\_STEROID\_HORMONE\_BIOSYNTHESIS | Details ... | 46 | -0.31 | -0.94 | 0.566 | 1.000 | 1.000 | 6892 | tags=30%, list=20%, signal=38% |
| 44 | KEGG\_DORSO\_VENTRAL\_AXIS\_FORMATION | Details ... | 24 | -0.29 | -0.94 | 0.542 | 1.000 | 1.000 | 752 | tags=8%, list=2%, signal=9% |
| 45 | KEGG\_VEGF\_SIGNALING\_PATHWAY | Details ... | 75 | -0.25 | -0.94 | 0.562 | 1.000 | 1.000 | 9361 | tags=27%, list=27%, signal=36% |
| 46 | KEGG\_NOTCH\_SIGNALING\_PATHWAY | Details ... | 47 | -0.25 | -0.93 | 0.521 | 1.000 | 1.000 | 638 | tags=4%, list=2%, signal=4% |
| 47 | KEGG\_ARACHIDONIC\_ACID\_METABOLISM | Details ... | 57 | -0.28 | -0.93 | 0.596 | 1.000 | 1.000 | 7378 | tags=23%, list=21%, signal=29% |
| 48 | KEGG\_EPITHELIAL\_CELL\_SIGNALING\_IN\_HELICOBACTER\_PYLORI\_INFECTION | Details ... | 66 | -0.27 | -0.92 | 0.539 | 1.000 | 1.000 | 11081 | tags=32%, list=32%, signal=47% |
| 49 | KEGG\_PROXIMAL\_TUBULE\_BICARBONATE\_RECLAMATION | Details ... | 23 | -0.30 | -0.92 | 0.617 | 1.000 | 1.000 | 8068 | tags=30%, list=23%, signal=40% |
| 50 | KEGG\_INTESTINAL\_IMMUNE\_NETWORK\_FOR\_IGA\_PRODUCTION | Details ... | 48 | -0.34 | -0.92 | 0.569 | 1.000 | 1.000 | 5176 | tags=25%, list=15%, signal=29% |
| 51 | KEGG\_AMYOTROPHIC\_LATERAL\_SCLEROSIS\_ALS |  | 53 | -0.26 | -0.92 | 0.576 | 1.000 | 1.000 | 9329 | tags=28%, list=27%, signal=39% |
| 52 | KEGG\_CELL\_ADHESION\_MOLECULES\_CAMS |  | 130 | -0.28 | -0.92 | 0.616 | 1.000 | 1.000 | 3781 | tags=16%, list=11%, signal=18% |
| 53 | KEGG\_TOLL\_LIKE\_RECEPTOR\_SIGNALING\_PATHWAY |  | 96 | -0.26 | -0.91 | 0.553 | 1.000 | 1.000 | 8343 | tags=26%, list=24%, signal=34% |
| 54 | KEGG\_GRAFT\_VERSUS\_HOST\_DISEASE |  | 39 | -0.36 | -0.91 | 0.586 | 1.000 | 1.000 | 7229 | tags=26%, list=21%, signal=32% |
| 55 | KEGG\_AMINO\_SUGAR\_AND\_NUCLEOTIDE\_SUGAR\_METABOLISM |  | 42 | -0.25 | -0.91 | 0.548 | 1.000 | 1.000 | 11748 | tags=38%, list=34%, signal=58% |
| 56 | KEGG\_ALZHEIMERS\_DISEASE |  | 144 | -0.18 | -0.90 | 0.607 | 1.000 | 1.000 | 8300 | tags=13%, list=24%, signal=16% |
| 57 | KEGG\_AMINOACYL\_TRNA\_BIOSYNTHESIS |  | 22 | -0.29 | -0.90 | 0.554 | 1.000 | 1.000 | 24500 | tags=100%, list=71%, signal=339% |
| 58 | KEGG\_PURINE\_METABOLISM |  | 151 | -0.20 | -0.88 | 0.621 | 1.000 | 1.000 | 11627 | tags=33%, list=33%, signal=50% |
| 59 | KEGG\_MELANOMA |  | 71 | -0.26 | -0.88 | 0.669 | 1.000 | 1.000 | 7236 | tags=21%, list=21%, signal=27% |
| 60 | KEGG\_DRUG\_METABOLISM\_OTHER\_ENZYMES |  | 42 | -0.28 | -0.86 | 0.679 | 1.000 | 1.000 | 3755 | tags=14%, list=11%, signal=16% |
| 61 | KEGG\_RIBOSOME |  | 85 | -0.24 | -0.86 | 0.633 | 1.000 | 1.000 | 16919 | tags=62%, list=49%, signal=121% |
| 62 | KEGG\_CALCIUM\_SIGNALING\_PATHWAY |  | 177 | -0.23 | -0.85 | 0.839 | 1.000 | 1.000 | 8341 | tags=28%, list=24%, signal=36% |
| 63 | KEGG\_FOCAL\_ADHESION |  | 196 | -0.24 | -0.84 | 0.645 | 1.000 | 1.000 | 9027 | tags=28%, list=26%, signal=37% |
| 64 | KEGG\_RENAL\_CELL\_CARCINOMA |  | 66 | -0.27 | -0.84 | 0.702 | 1.000 | 1.000 | 12116 | tags=45%, list=35%, signal=70% |
| 65 | KEGG\_CHEMOKINE\_SIGNALING\_PATHWAY |  | 183 | -0.23 | -0.83 | 0.744 | 1.000 | 1.000 | 9275 | tags=27%, list=27%, signal=36% |
| 66 | KEGG\_FC\_GAMMA\_R\_MEDIATED\_PHAGOCYTOSIS |  | 92 | -0.20 | -0.82 | 0.743 | 1.000 | 1.000 | 9027 | tags=21%, list=26%, signal=28% |
| 67 | KEGG\_MATURITY\_ONSET\_DIABETES\_OF\_THE\_YOUNG |  | 25 | -0.32 | -0.81 | 0.737 | 1.000 | 1.000 | 7446 | tags=32%, list=21%, signal=41% |
| 68 | KEGG\_PROTEASOME |  | 43 | -0.23 | -0.81 | 0.724 | 1.000 | 1.000 | 26726 | tags=100%, list=77%, signal=433% |
| 69 | KEGG\_SYSTEMIC\_LUPUS\_ERYTHEMATOSUS |  | 56 | -0.32 | -0.80 | 0.701 | 1.000 | 1.000 | 4308 | tags=18%, list=12%, signal=20% |
| 70 | KEGG\_REGULATION\_OF\_ACTIN\_CYTOSKELETON |  | 210 | -0.18 | -0.80 | 0.808 | 1.000 | 1.000 | 9148 | tags=25%, list=26%, signal=34% |
| 71 | KEGG\_PATHOGENIC\_ESCHERICHIA\_COLI\_INFECTION |  | 54 | -0.18 | -0.80 | 0.707 | 1.000 | 1.000 | 3934 | tags=7%, list=11%, signal=8% |
| 72 | KEGG\_NATURAL\_KILLER\_CELL\_MEDIATED\_CYTOTOXICITY |  | 125 | -0.21 | -0.80 | 0.852 | 1.000 | 1.000 | 8535 | tags=22%, list=25%, signal=30% |
| 73 | KEGG\_NICOTINATE\_AND\_NICOTINAMIDE\_METABOLISM |  | 24 | -0.30 | -0.80 | 0.671 | 1.000 | 1.000 | 4349 | tags=17%, list=13%, signal=19% |
| 74 | KEGG\_CITRATE\_CYCLE\_TCA\_CYCLE |  | 30 | -0.20 | -0.80 | 0.704 | 1.000 | 1.000 | 12674 | tags=37%, list=36%, signal=58% |
| 75 | KEGG\_VIRAL\_MYOCARDITIS |  | 70 | -0.25 | -0.79 | 0.750 | 1.000 | 1.000 | 3943 | tags=13%, list=11%, signal=14% |
| 76 | KEGG\_WNT\_SIGNALING\_PATHWAY |  | 149 | -0.18 | -0.79 | 0.841 | 1.000 | 1.000 | 11288 | tags=34%, list=33%, signal=50% |
| 77 | KEGG\_PYRIMIDINE\_METABOLISM |  | 95 | -0.18 | -0.79 | 0.696 | 1.000 | 1.000 | 11939 | tags=34%, list=34%, signal=51% |
| 78 | KEGG\_CYTOSOLIC\_DNA\_SENSING\_PATHWAY |  | 47 | -0.23 | -0.78 | 0.781 | 1.000 | 1.000 | 1500 | tags=9%, list=4%, signal=9% |
| 79 | KEGG\_ECM\_RECEPTOR\_INTERACTION |  | 84 | -0.24 | -0.77 | 0.758 | 1.000 | 1.000 | 9024 | tags=38%, list=26%, signal=51% |
| 80 | KEGG\_CHRONIC\_MYELOID\_LEUKEMIA |  | 73 | -0.19 | -0.76 | 0.738 | 1.000 | 1.000 | 10276 | tags=29%, list=30%, signal=41% |
| 81 | KEGG\_TRYPTOPHAN\_METABOLISM |  | 39 | -0.23 | -0.76 | 0.894 | 1.000 | 1.000 | 3580 | tags=10%, list=10%, signal=11% |
| 82 | KEGG\_LONG\_TERM\_POTENTIATION |  | 70 | -0.18 | -0.75 | 0.868 | 1.000 | 1.000 | 12116 | tags=37%, list=35%, signal=57% |
| 83 | KEGG\_PARKINSONS\_DISEASE |  | 98 | -0.18 | -0.75 | 0.708 | 1.000 | 1.000 | 15714 | tags=43%, list=45%, signal=78% |
| 84 | KEGG\_ACUTE\_MYELOID\_LEUKEMIA |  | 57 | -0.18 | -0.75 | 0.770 | 1.000 | 1.000 | 12116 | tags=39%, list=35%, signal=59% |
| 85 | KEGG\_ASTHMA |  | 29 | -0.34 | -0.75 | 0.781 | 0.993 | 1.000 | 3781 | tags=21%, list=11%, signal=23% |
| 86 | KEGG\_SPHINGOLIPID\_METABOLISM |  | 35 | -0.21 | -0.75 | 0.782 | 0.982 | 1.000 | 8982 | tags=26%, list=26%, signal=35% |
| 87 | KEGG\_ADIPOCYTOKINE\_SIGNALING\_PATHWAY |  | 66 | -0.21 | -0.74 | 0.787 | 0.984 | 1.000 | 8739 | tags=27%, list=25%, signal=36% |
| 88 | KEGG\_ENDOCYTOSIS |  | 179 | -0.13 | -0.73 | 0.842 | 0.990 | 1.000 | 3561 | tags=6%, list=10%, signal=6% |
| 89 | KEGG\_GLIOMA |  | 65 | -0.18 | -0.72 | 0.917 | 0.989 | 1.000 | 7437 | tags=17%, list=21%, signal=21% |
| 90 | KEGG\_FATTY\_ACID\_METABOLISM |  | 42 | -0.21 | -0.70 | 0.823 | 1.000 | 1.000 | 11112 | tags=36%, list=32%, signal=52% |
| 91 | KEGG\_CELL\_CYCLE |  | 124 | -0.19 | -0.67 | 0.767 | 1.000 | 1.000 | 11059 | tags=32%, list=32%, signal=47% |
| 92 | KEGG\_AXON\_GUIDANCE |  | 128 | -0.17 | -0.66 | 0.975 | 1.000 | 1.000 | 9398 | tags=26%, list=27%, signal=35% |
| 93 | KEGG\_RNA\_POLYMERASE |  | 27 | -0.21 | -0.66 | 0.962 | 1.000 | 1.000 | 27451 | tags=100%, list=79%, signal=477% |
| 94 | KEGG\_PENTOSE\_PHOSPHATE\_PATHWAY |  | 27 | -0.17 | -0.65 | 0.944 | 1.000 | 1.000 | 4453 | tags=7%, list=13%, signal=8% |
| 95 | KEGG\_GLYCEROPHOSPHOLIPID\_METABOLISM |  | 70 | -0.17 | -0.64 | 0.945 | 1.000 | 1.000 | 7011 | tags=14%, list=20%, signal=18% |
| 96 | KEGG\_DNA\_REPLICATION |  | 36 | -0.19 | -0.64 | 0.866 | 1.000 | 1.000 | 12760 | tags=47%, list=37%, signal=75% |
| 97 | KEGG\_PRION\_DISEASES |  | 35 | -0.22 | -0.63 | 0.967 | 1.000 | 1.000 | 10104 | tags=31%, list=29%, signal=44% |
| 98 | KEGG\_GLYCOSYLPHOSPHATIDYLINOSITOL\_GPI\_ANCHOR\_BIOSYNTHESIS |  | 25 | -0.20 | -0.62 | 0.848 | 1.000 | 1.000 | 10054 | tags=28%, list=29%, signal=39% |
| 99 | KEGG\_ERBB\_SIGNALING\_PATHWAY |  | 86 | -0.14 | -0.62 | 0.973 | 1.000 | 1.000 | 11687 | tags=33%, list=34%, signal=49% |
| 100 | KEGG\_LEISHMANIA\_INFECTION |  | 71 | -0.22 | -0.61 | 0.957 | 0.997 | 1.000 | 8211 | tags=25%, list=24%, signal=33% |
| 101 | KEGG\_PROPANOATE\_METABOLISM |  | 31 | -0.19 | -0.61 | 0.854 | 0.989 | 1.000 | 12423 | tags=39%, list=36%, signal=60% |
| 102 | KEGG\_O\_GLYCAN\_BIOSYNTHESIS |  | 29 | -0.21 | -0.60 | 0.924 | 0.985 | 1.000 | 6017 | tags=17%, list=17%, signal=21% |
| 103 | KEGG\_N\_GLYCAN\_BIOSYNTHESIS |  | 46 | -0.18 | -0.60 | 0.810 | 0.976 | 1.000 | 9980 | tags=22%, list=29%, signal=30% |
| 104 | KEGG\_NOD\_LIKE\_RECEPTOR\_SIGNALING\_PATHWAY |  | 61 | -0.18 | -0.56 | 0.895 | 0.988 | 1.000 | 12116 | tags=36%, list=35%, signal=55% |
| 105 | KEGG\_LYSOSOME |  | 117 | -0.11 | -0.56 | 0.910 | 0.979 | 1.000 | 13723 | tags=32%, list=40%, signal=54% |
| 106 | KEGG\_FRUCTOSE\_AND\_MANNOSE\_METABOLISM |  | 32 | -0.19 | -0.56 | 0.953 | 0.970 | 1.000 | 8108 | tags=22%, list=23%, signal=29% |
Table: Gene sets enriched in phenotype **l (8 samples)**[plain text format]****

  
