# Supplementary figures and images for: Multi‐dataset identification of innovative feature genes and molecular mechanisms in keratoconus (part 1 of 2)
Source: J Cell Mol Med. 2024 Sep 19;28(18):e70079. doi: 10.1111/jcmm.70079 (PMC11412914; doi:10.1111/jcmm.70079)

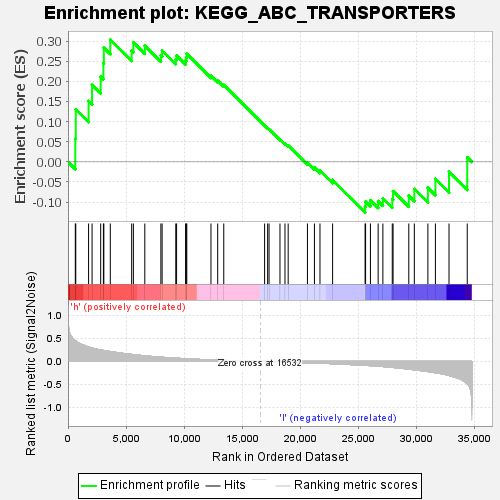

Supplement: Supplementary file 2 — Material S2. [file JCMM-28-e70079-s002.zip › 7.GSEA analysis/1.ARL11/enplot_KEGG_ABC_TRANSPORTERS_90.png]

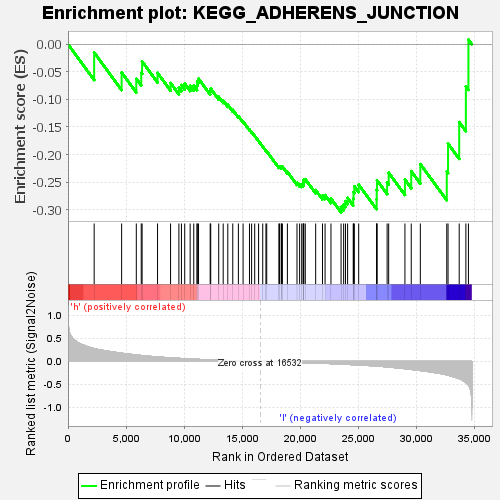

Supplement: Supplementary file 2 — Material S2. [file JCMM-28-e70079-s002.zip › 7.GSEA analysis/1.ARL11/enplot_KEGG_ADHERENS_JUNCTION_177.png]

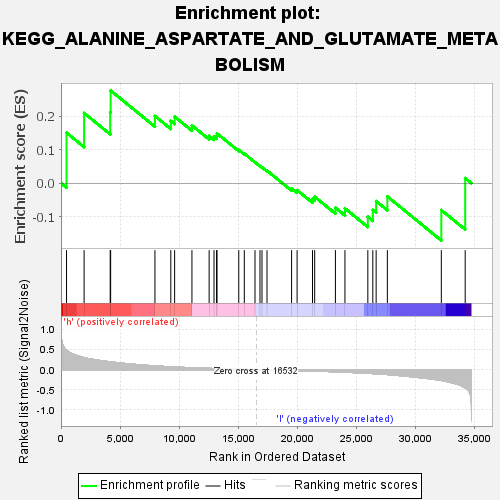

Supplement: Supplementary file 2 — Material S2. [file JCMM-28-e70079-s002.zip › 7.GSEA analysis/1.ARL11/enplot_KEGG_ALANINE_ASPARTATE_AND_GLUTAMATE_METABOLISM_75.png]

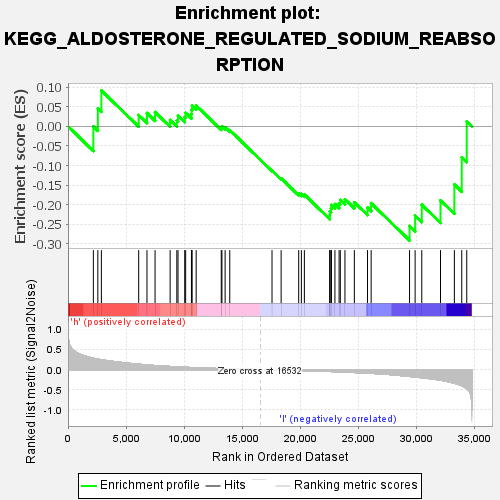

Supplement: Supplementary file 2 — Material S2. [file JCMM-28-e70079-s002.zip › 7.GSEA analysis/1.ARL11/enplot_KEGG_ALDOSTERONE_REGULATED_SODIUM_REABSORPTION_273.png]

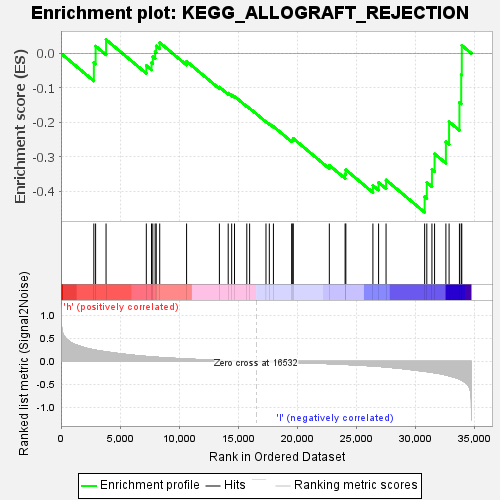

Supplement: Supplementary file 2 — Material S2. [file JCMM-28-e70079-s002.zip › 7.GSEA analysis/1.ARL11/enplot_KEGG_ALLOGRAFT_REJECTION_204.png]

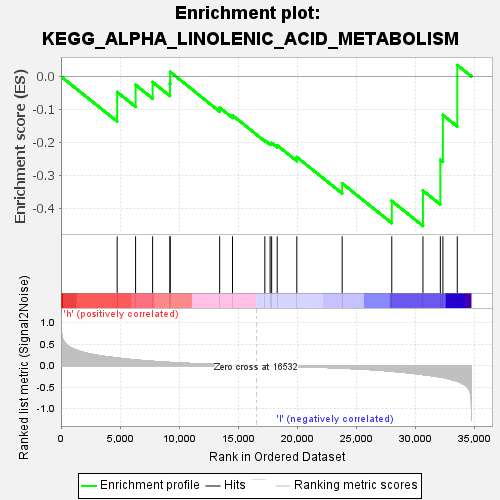

Supplement: Supplementary file 2 — Material S2. [file JCMM-28-e70079-s002.zip › 7.GSEA analysis/1.ARL11/enplot_KEGG_ALPHA_LINOLENIC_ACID_METABOLISM_219.png]

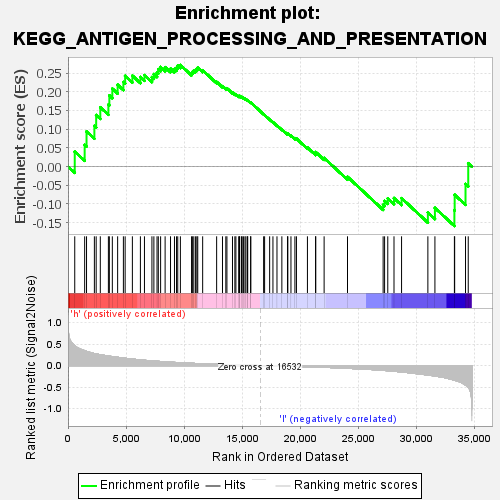

Supplement: Supplementary file 2 — Material S2. [file JCMM-28-e70079-s002.zip › 7.GSEA analysis/1.ARL11/enplot_KEGG_ANTIGEN_PROCESSING_AND_PRESENTATION_93.png]

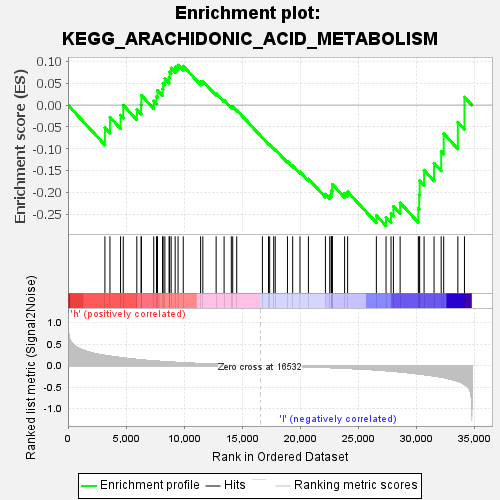

Supplement: Supplementary file 2 — Material S2. [file JCMM-28-e70079-s002.zip › 7.GSEA analysis/1.ARL11/enplot_KEGG_ARACHIDONIC_ACID_METABOLISM_291.png]

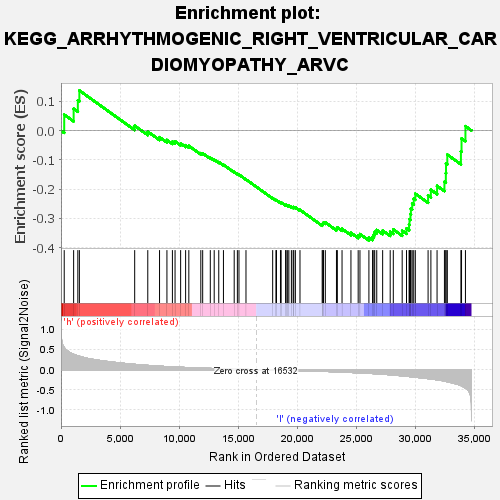

Supplement: Supplementary file 2 — Material S2. [file JCMM-28-e70079-s002.zip › 7.GSEA analysis/1.ARL11/enplot_KEGG_ARRHYTHMOGENIC_RIGHT_VENTRICULAR_CARDIOMYOPATHY_ARVC_183.png]

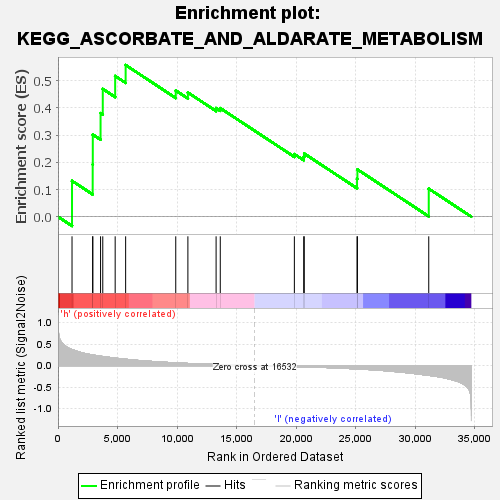

Supplement: Supplementary file 2 — Material S2. [file JCMM-28-e70079-s002.zip › 7.GSEA analysis/1.ARL11/enplot_KEGG_ASCORBATE_AND_ALDARATE_METABOLISM_9.png]

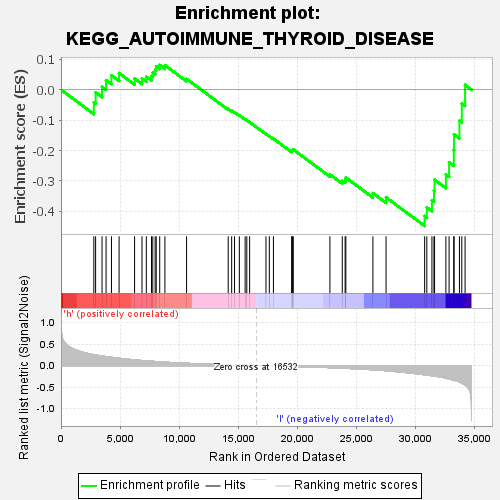

Supplement: Supplementary file 2 — Material S2. [file JCMM-28-e70079-s002.zip › 7.GSEA analysis/1.ARL11/enplot_KEGG_AUTOIMMUNE_THYROID_DISEASE_171.png]

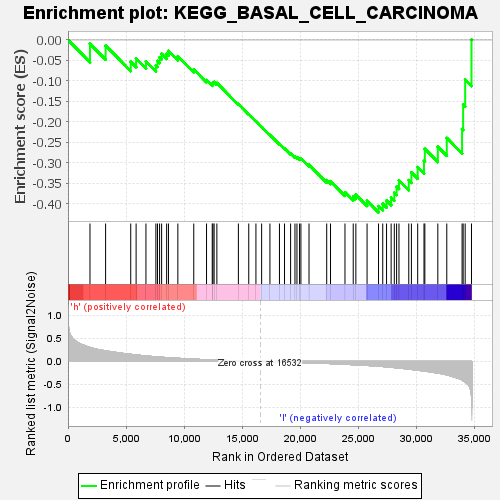

Supplement: Supplementary file 2 — Material S2. [file JCMM-28-e70079-s002.zip › 7.GSEA analysis/1.ARL11/enplot_KEGG_BASAL_CELL_CARCINOMA_168.png]

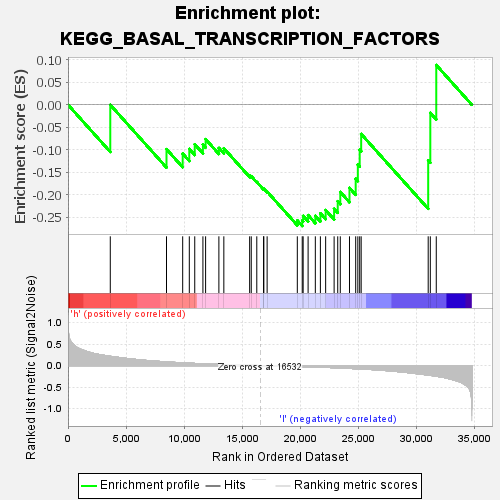

Supplement: Supplementary file 2 — Material S2. [file JCMM-28-e70079-s002.zip › 7.GSEA analysis/1.ARL11/enplot_KEGG_BASAL_TRANSCRIPTION_FACTORS_198.png]

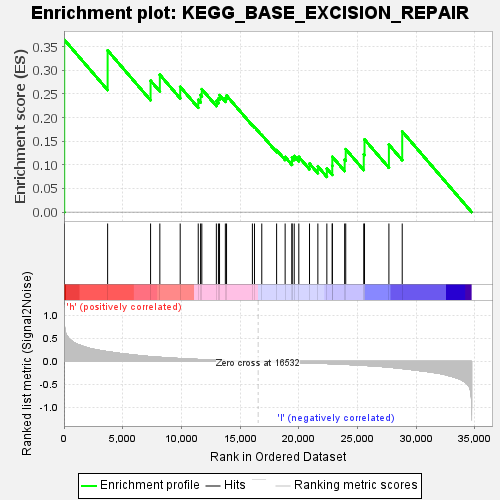

Supplement: Supplementary file 2 — Material S2. [file JCMM-28-e70079-s002.zip › 7.GSEA analysis/1.ARL11/enplot_KEGG_BASE_EXCISION_REPAIR_21.png]

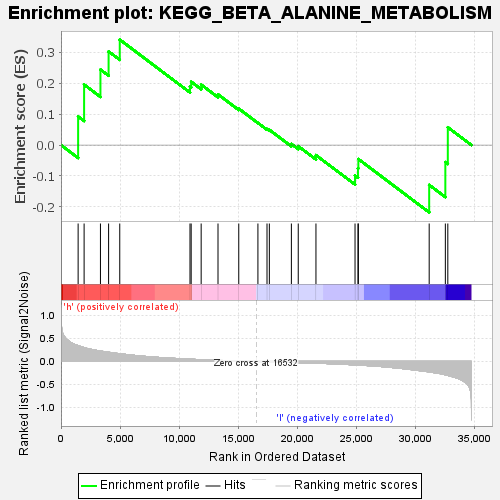

Supplement: Supplementary file 2 — Material S2. [file JCMM-28-e70079-s002.zip › 7.GSEA analysis/1.ARL11/enplot_KEGG_BETA_ALANINE_METABOLISM_63.png]

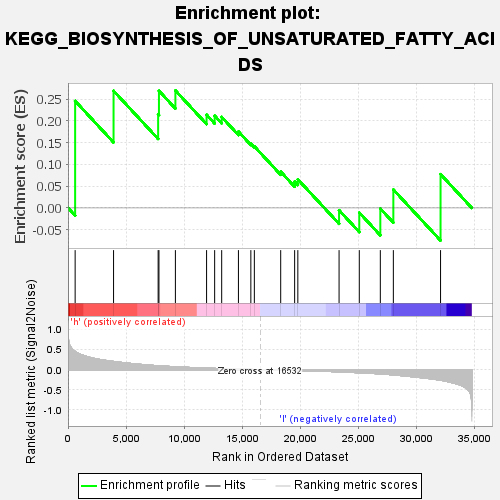

Supplement: Supplementary file 2 — Material S2. [file JCMM-28-e70079-s002.zip › 7.GSEA analysis/1.ARL11/enplot_KEGG_BIOSYNTHESIS_OF_UNSATURATED_FATTY_ACIDS_72.png]

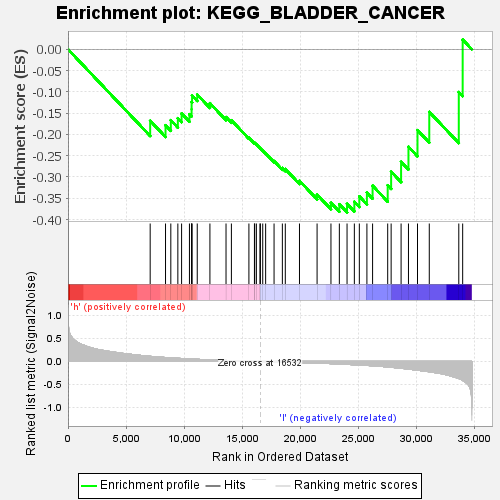

Supplement: Supplementary file 2 — Material S2. [file JCMM-28-e70079-s002.zip › 7.GSEA analysis/1.ARL11/enplot_KEGG_BLADDER_CANCER_216.png]

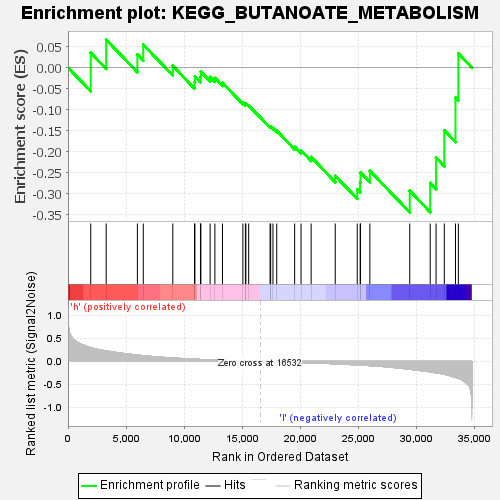

Supplement: Supplementary file 2 — Material S2. [file JCMM-28-e70079-s002.zip › 7.GSEA analysis/1.ARL11/enplot_KEGG_BUTANOATE_METABOLISM_255.png]

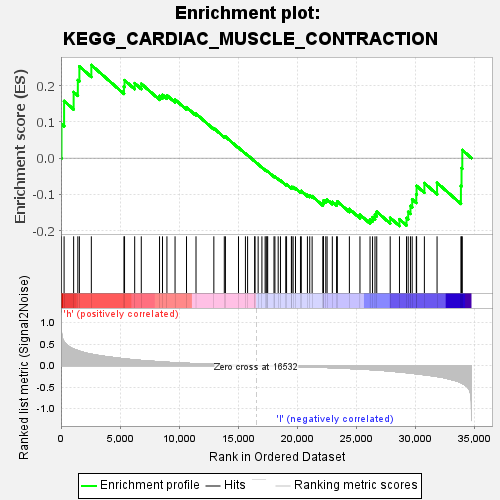

Supplement: Supplementary file 2 — Material S2. [file JCMM-28-e70079-s002.zip › 7.GSEA analysis/1.ARL11/enplot_KEGG_CARDIAC_MUSCLE_CONTRACTION_54.png]

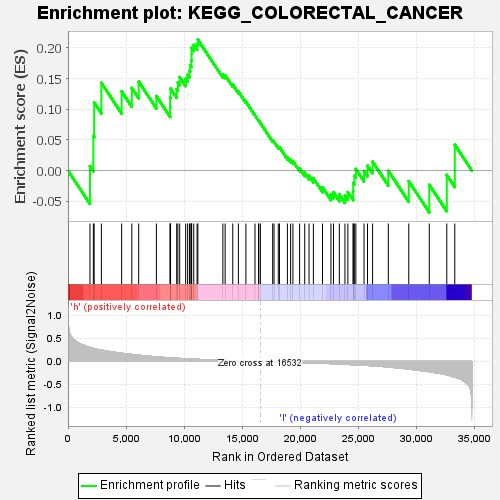

Supplement: Supplementary file 2 — Material S2. [file JCMM-28-e70079-s002.zip › 7.GSEA analysis/1.ARL11/enplot_KEGG_COLORECTAL_CANCER_108.png]

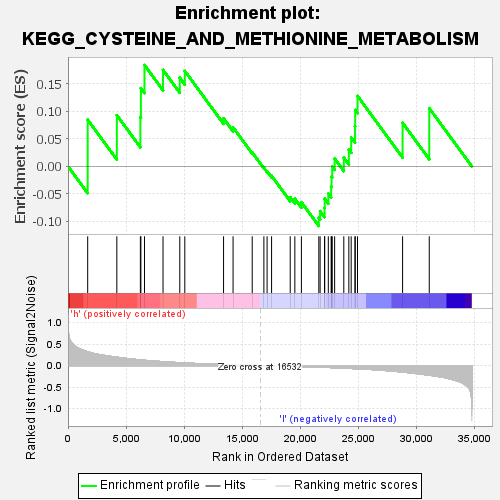

Supplement: Supplementary file 2 — Material S2. [file JCMM-28-e70079-s002.zip › 7.GSEA analysis/1.ARL11/enplot_KEGG_CYSTEINE_AND_METHIONINE_METABOLISM_135.png]

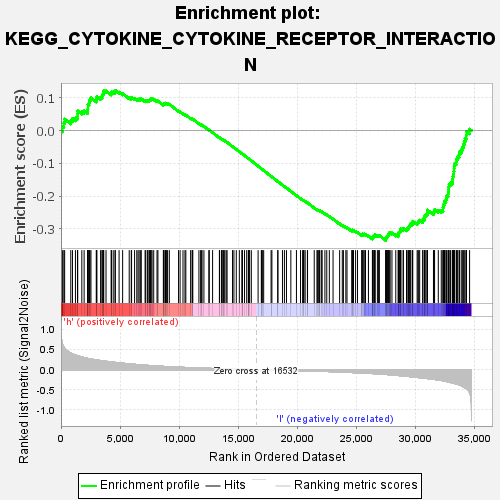

Supplement: Supplementary file 2 — Material S2. [file JCMM-28-e70079-s002.zip › 7.GSEA analysis/1.ARL11/enplot_KEGG_CYTOKINE_CYTOKINE_RECEPTOR_INTERACTION_249.png]

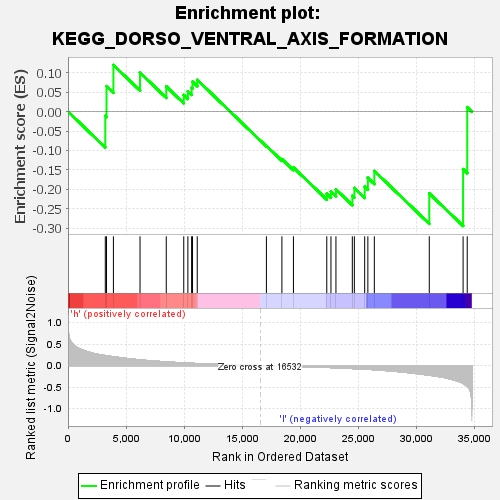

Supplement: Supplementary file 2 — Material S2. [file JCMM-28-e70079-s002.zip › 7.GSEA analysis/1.ARL11/enplot_KEGG_DORSO_VENTRAL_AXIS_FORMATION_282.png]

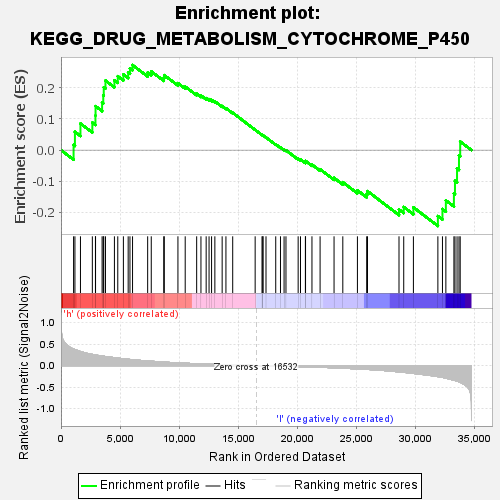

Supplement: Supplementary file 2 — Material S2. [file JCMM-28-e70079-s002.zip › 7.GSEA analysis/1.ARL11/enplot_KEGG_DRUG_METABOLISM_CYTOCHROME_P450_96.png]

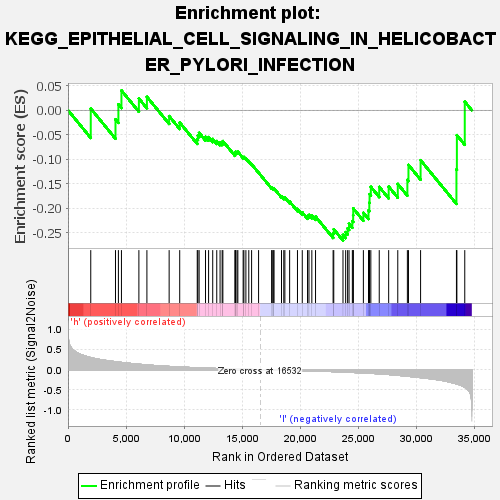

Supplement: Supplementary file 2 — Material S2. [file JCMM-28-e70079-s002.zip › 7.GSEA analysis/1.ARL11/enplot_KEGG_EPITHELIAL_CELL_SIGNALING_IN_HELICOBACTER_PYLORI_INFECTION_294.png]

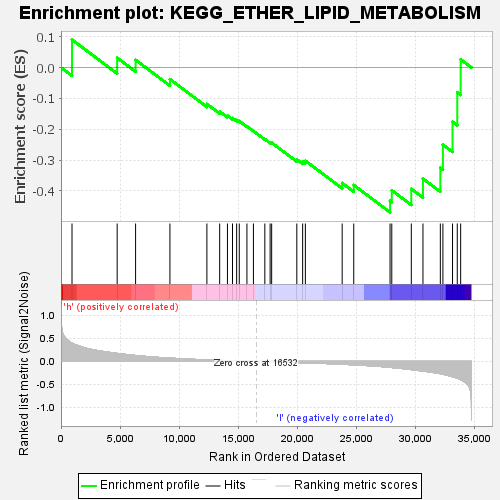

Supplement: Supplementary file 2 — Material S2. [file JCMM-28-e70079-s002.zip › 7.GSEA analysis/1.ARL11/enplot_KEGG_ETHER_LIPID_METABOLISM_174.png]

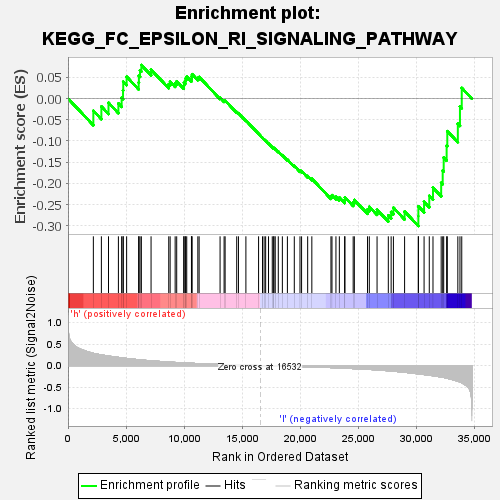

Supplement: Supplementary file 2 — Material S2. [file JCMM-28-e70079-s002.zip › 7.GSEA analysis/1.ARL11/enplot_KEGG_FC_EPSILON_RI_SIGNALING_PATHWAY_222.png]

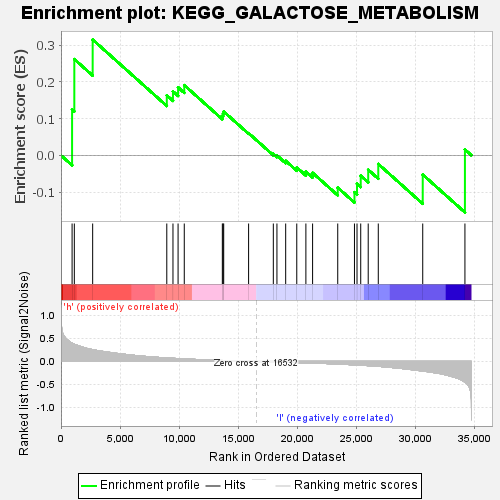

Supplement: Supplementary file 2 — Material S2. [file JCMM-28-e70079-s002.zip › 7.GSEA analysis/1.ARL11/enplot_KEGG_GALACTOSE_METABOLISM_48.png]

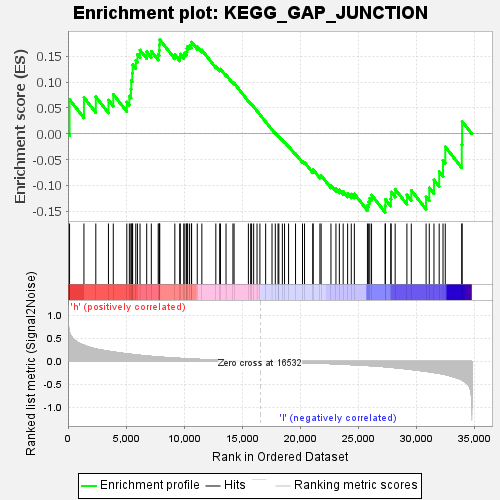

Supplement: Supplementary file 2 — Material S2. [file JCMM-28-e70079-s002.zip › 7.GSEA analysis/1.ARL11/enplot_KEGG_GAP_JUNCTION_123.png]

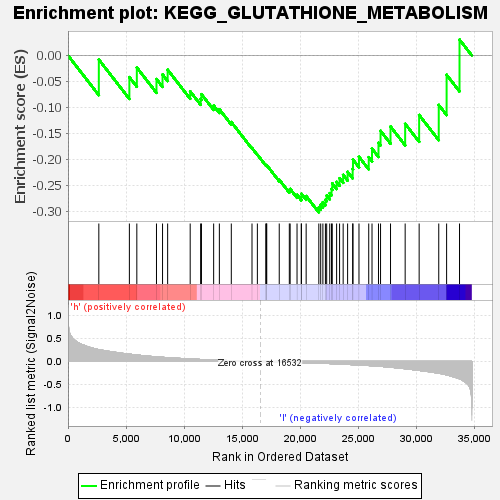

Supplement: Supplementary file 2 — Material S2. [file JCMM-28-e70079-s002.zip › 7.GSEA analysis/1.ARL11/enplot_KEGG_GLUTATHIONE_METABOLISM_252.png]

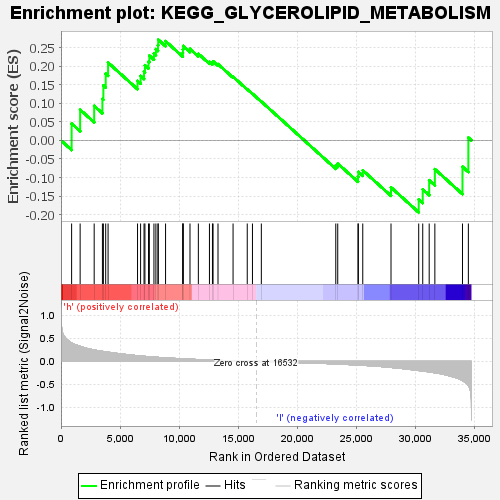

Supplement: Supplementary file 2 — Material S2. [file JCMM-28-e70079-s002.zip › 7.GSEA analysis/1.ARL11/enplot_KEGG_GLYCEROLIPID_METABOLISM_66.png]

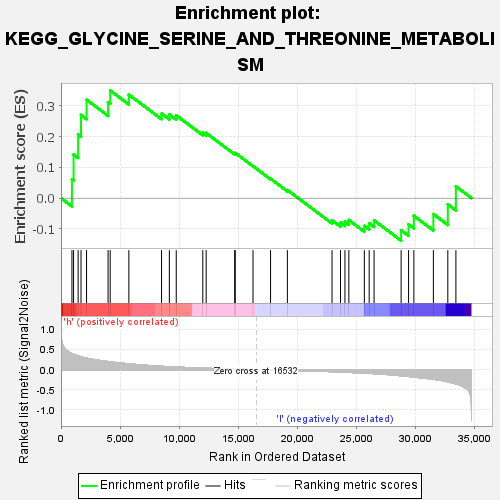

Supplement: Supplementary file 2 — Material S2. [file JCMM-28-e70079-s002.zip › 7.GSEA analysis/1.ARL11/enplot_KEGG_GLYCINE_SERINE_AND_THREONINE_METABOLISM_15.png]

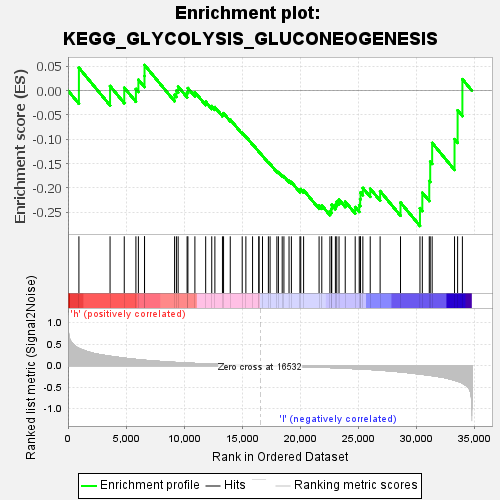

Supplement: Supplementary file 2 — Material S2. [file JCMM-28-e70079-s002.zip › 7.GSEA analysis/1.ARL11/enplot_KEGG_GLYCOLYSIS_GLUCONEOGENESIS_246.png]

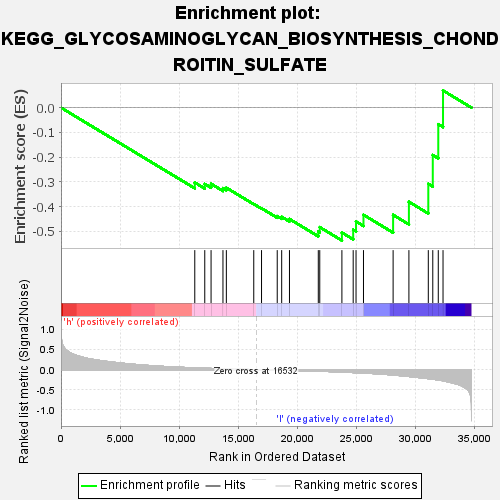

Supplement: Supplementary file 2 — Material S2. [file JCMM-28-e70079-s002.zip › 7.GSEA analysis/1.ARL11/enplot_KEGG_GLYCOSAMINOGLYCAN_BIOSYNTHESIS_CHONDROITIN_SULFATE_156.png]

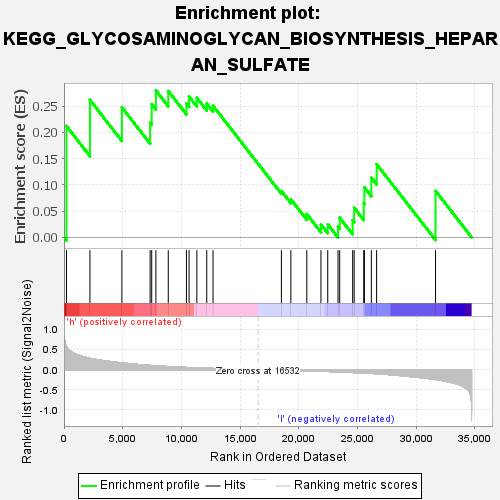

Supplement: Supplementary file 2 — Material S2. [file JCMM-28-e70079-s002.zip › 7.GSEA analysis/1.ARL11/enplot_KEGG_GLYCOSAMINOGLYCAN_BIOSYNTHESIS_HEPARAN_SULFATE_81.png]

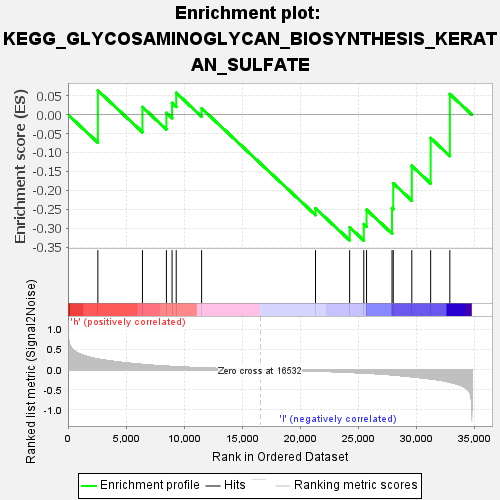

Supplement: Supplementary file 2 — Material S2. [file JCMM-28-e70079-s002.zip › 7.GSEA analysis/1.ARL11/enplot_KEGG_GLYCOSAMINOGLYCAN_BIOSYNTHESIS_KERATAN_SULFATE_276.png]

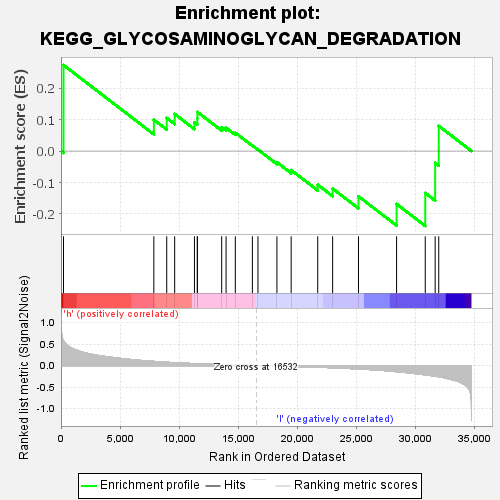

Supplement: Supplementary file 2 — Material S2. [file JCMM-28-e70079-s002.zip › 7.GSEA analysis/1.ARL11/enplot_KEGG_GLYCOSAMINOGLYCAN_DEGRADATION_114.png]

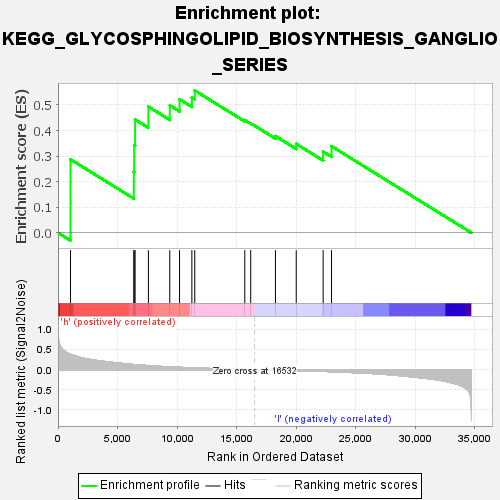

Supplement: Supplementary file 2 — Material S2. [file JCMM-28-e70079-s002.zip › 7.GSEA analysis/1.ARL11/enplot_KEGG_GLYCOSPHINGOLIPID_BIOSYNTHESIS_GANGLIO_SERIES_6.png]

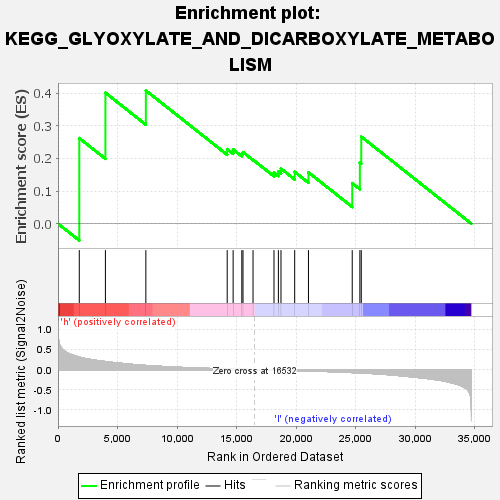

Supplement: Supplementary file 2 — Material S2. [file JCMM-28-e70079-s002.zip › 7.GSEA analysis/1.ARL11/enplot_KEGG_GLYOXYLATE_AND_DICARBOXYLATE_METABOLISM_18.png]

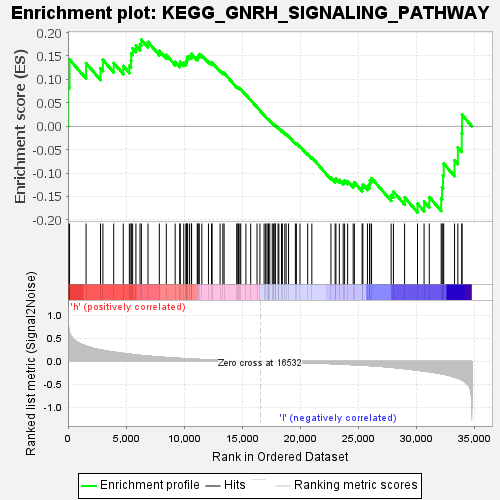

Supplement: Supplementary file 2 — Material S2. [file JCMM-28-e70079-s002.zip › 7.GSEA analysis/1.ARL11/enplot_KEGG_GNRH_SIGNALING_PATHWAY_117.png]

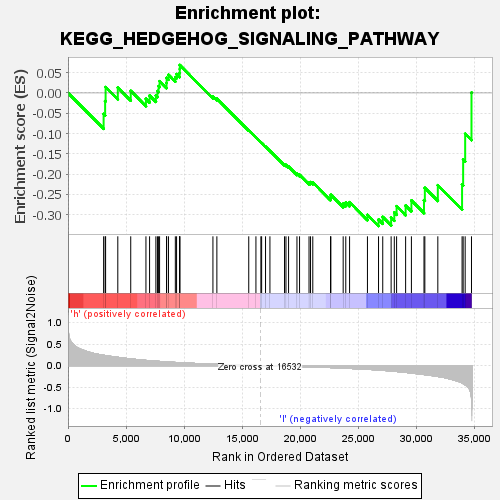

Supplement: Supplementary file 2 — Material S2. [file JCMM-28-e70079-s002.zip › 7.GSEA analysis/1.ARL11/enplot_KEGG_HEDGEHOG_SIGNALING_PATHWAY_237.png]

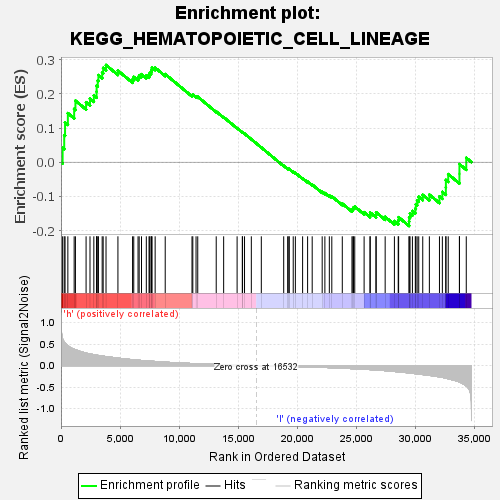

Supplement: Supplementary file 2 — Material S2. [file JCMM-28-e70079-s002.zip › 7.GSEA analysis/1.ARL11/enplot_KEGG_HEMATOPOIETIC_CELL_LINEAGE_105.png]

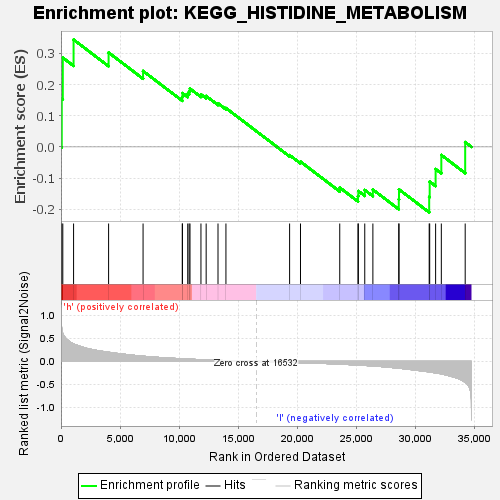

Supplement: Supplementary file 2 — Material S2. [file JCMM-28-e70079-s002.zip › 7.GSEA analysis/1.ARL11/enplot_KEGG_HISTIDINE_METABOLISM_78.png]

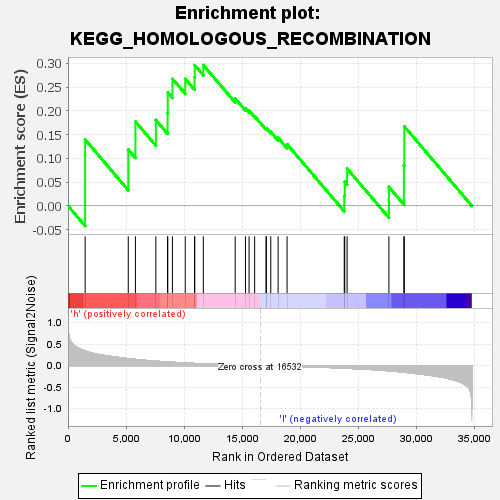

Supplement: Supplementary file 2 — Material S2. [file JCMM-28-e70079-s002.zip › 7.GSEA analysis/1.ARL11/enplot_KEGG_HOMOLOGOUS_RECOMBINATION_45.png]

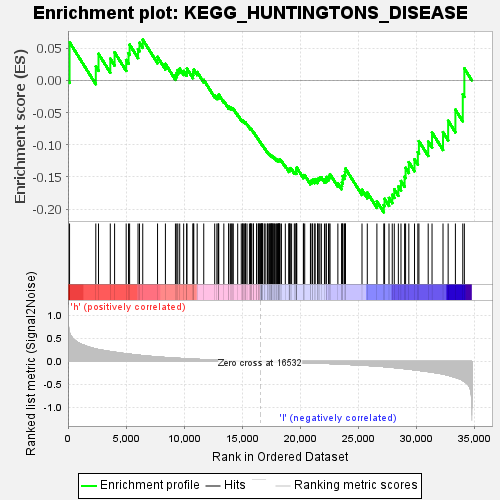

Supplement: Supplementary file 2 — Material S2. [file JCMM-28-e70079-s002.zip › 7.GSEA analysis/1.ARL11/enplot_KEGG_HUNTINGTONS_DISEASE_189.png]

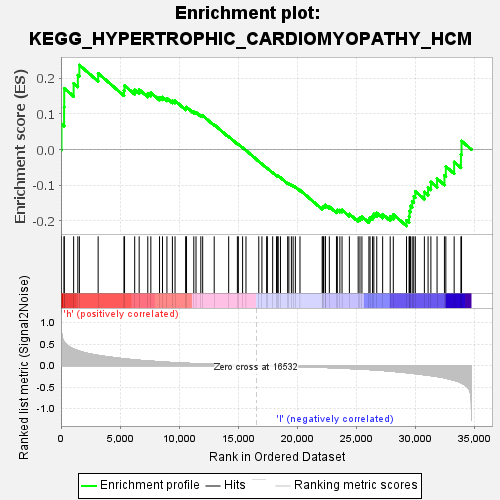

Supplement: Supplementary file 2 — Material S2. [file JCMM-28-e70079-s002.zip › 7.GSEA analysis/1.ARL11/enplot_KEGG_HYPERTROPHIC_CARDIOMYOPATHY_HCM_132.png]

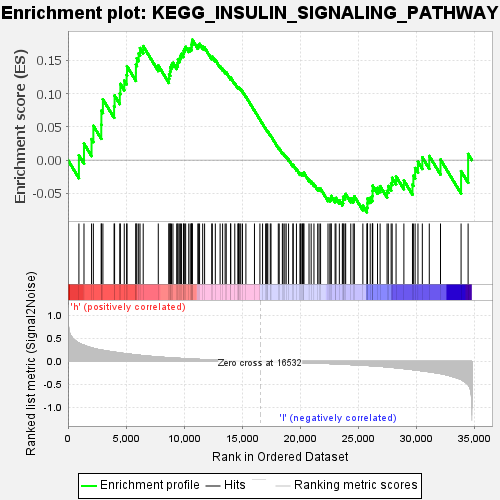

Supplement: Supplementary file 2 — Material S2. [file JCMM-28-e70079-s002.zip › 7.GSEA analysis/1.ARL11/enplot_KEGG_INSULIN_SIGNALING_PATHWAY_138.png]

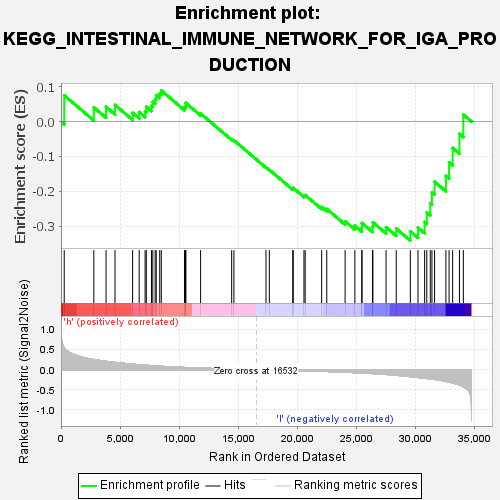

Supplement: Supplementary file 2 — Material S2. [file JCMM-28-e70079-s002.zip › 7.GSEA analysis/1.ARL11/enplot_KEGG_INTESTINAL_IMMUNE_NETWORK_FOR_IGA_PRODUCTION_300.png]

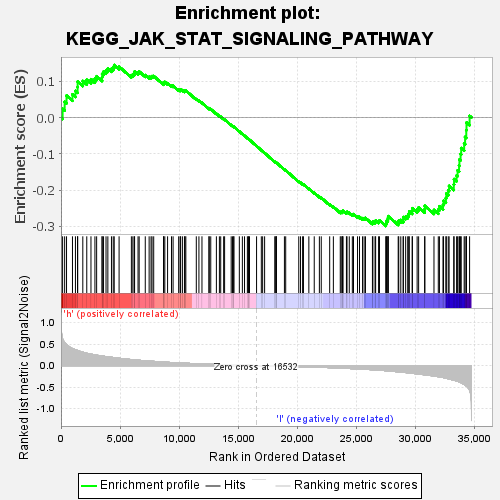

Supplement: Supplementary file 2 — Material S2. [file JCMM-28-e70079-s002.zip › 7.GSEA analysis/1.ARL11/enplot_KEGG_JAK_STAT_SIGNALING_PATHWAY_258.png]

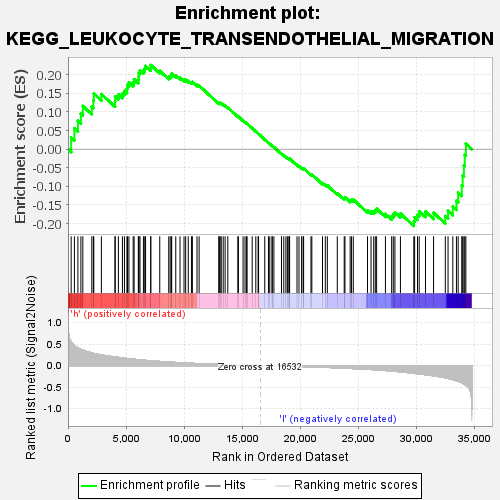

Supplement: Supplementary file 2 — Material S2. [file JCMM-28-e70079-s002.zip › 7.GSEA analysis/1.ARL11/enplot_KEGG_LEUKOCYTE_TRANSENDOTHELIAL_MIGRATION_111.png]

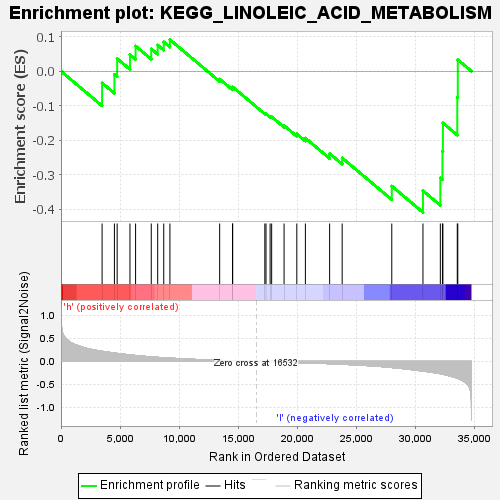

Supplement: Supplementary file 2 — Material S2. [file JCMM-28-e70079-s002.zip › 7.GSEA analysis/1.ARL11/enplot_KEGG_LINOLEIC_ACID_METABOLISM_210.png]

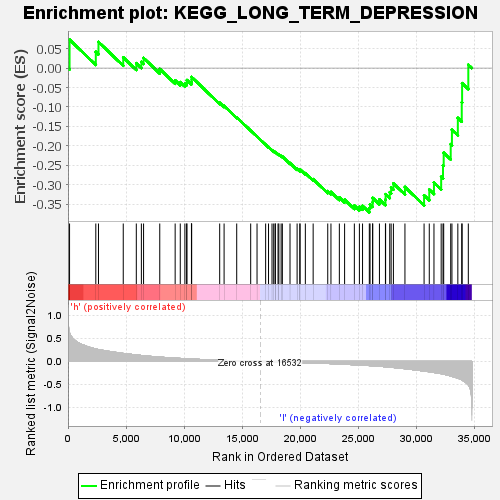

Supplement: Supplementary file 2 — Material S2. [file JCMM-28-e70079-s002.zip › 7.GSEA analysis/1.ARL11/enplot_KEGG_LONG_TERM_DEPRESSION_165.png]

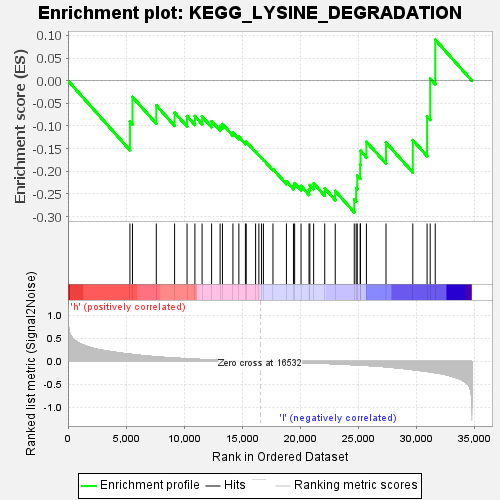

Supplement: Supplementary file 2 — Material S2. [file JCMM-28-e70079-s002.zip › 7.GSEA analysis/1.ARL11/enplot_KEGG_LYSINE_DEGRADATION_270.png]

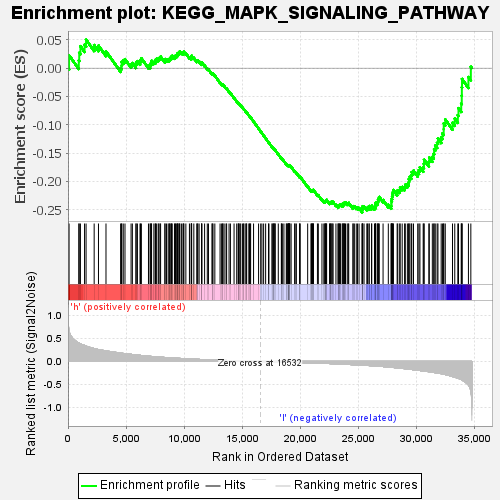

Supplement: Supplementary file 2 — Material S2. [file JCMM-28-e70079-s002.zip › 7.GSEA analysis/1.ARL11/enplot_KEGG_MAPK_SIGNALING_PATHWAY_231.png]

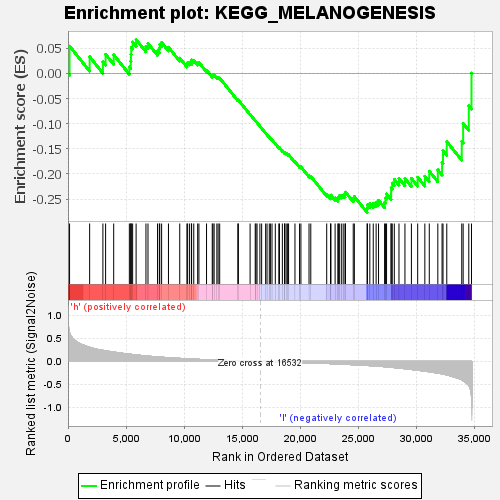

Supplement: Supplementary file 2 — Material S2. [file JCMM-28-e70079-s002.zip › 7.GSEA analysis/1.ARL11/enplot_KEGG_MELANOGENESIS_225.png]

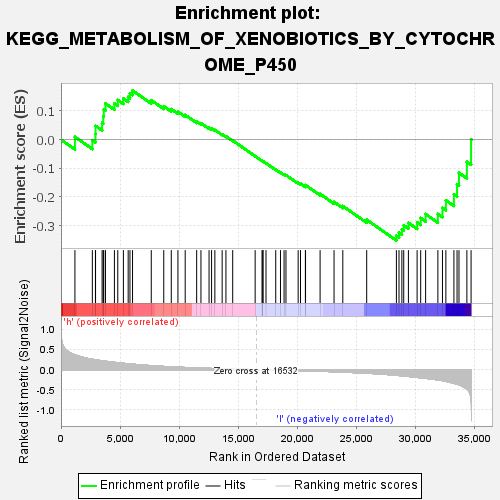

Supplement: Supplementary file 2 — Material S2. [file JCMM-28-e70079-s002.zip › 7.GSEA analysis/1.ARL11/enplot_KEGG_METABOLISM_OF_XENOBIOTICS_BY_CYTOCHROME_P450_201.png]

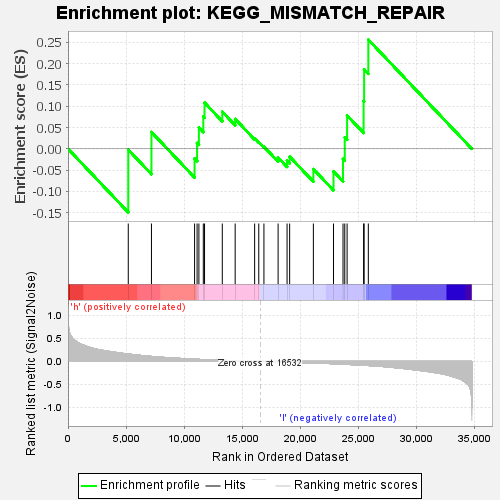

Supplement: Supplementary file 2 — Material S2. [file JCMM-28-e70079-s002.zip › 7.GSEA analysis/1.ARL11/enplot_KEGG_MISMATCH_REPAIR_84.png]

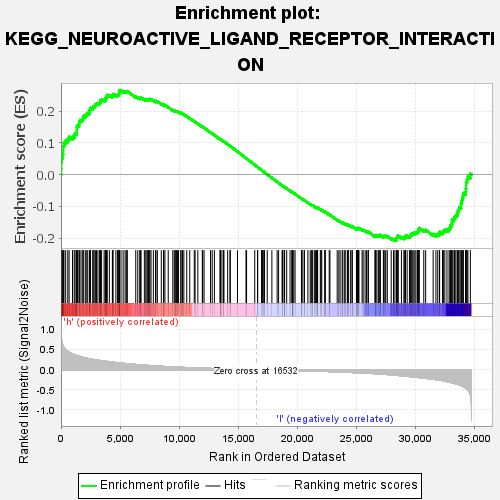

Supplement: Supplementary file 2 — Material S2. [file JCMM-28-e70079-s002.zip › 7.GSEA analysis/1.ARL11/enplot_KEGG_NEUROACTIVE_LIGAND_RECEPTOR_INTERACTION_57.png]

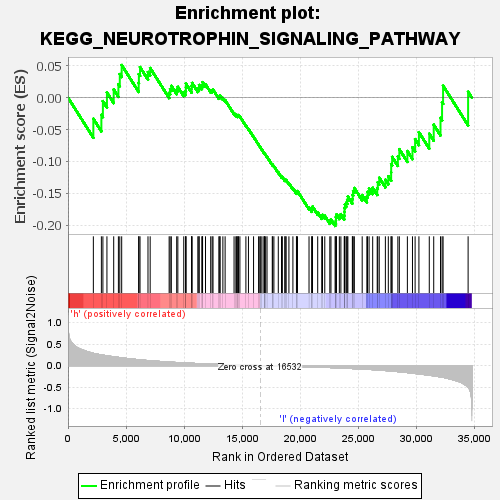

Supplement: Supplementary file 2 — Material S2. [file JCMM-28-e70079-s002.zip › 7.GSEA analysis/1.ARL11/enplot_KEGG_NEUROTROPHIN_SIGNALING_PATHWAY_261.png]

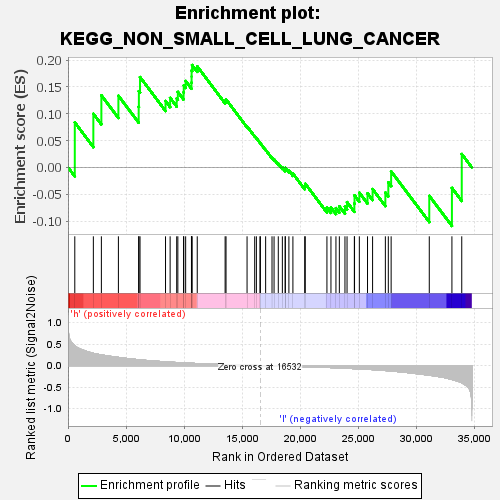

Supplement: Supplementary file 2 — Material S2. [file JCMM-28-e70079-s002.zip › 7.GSEA analysis/1.ARL11/enplot_KEGG_NON_SMALL_CELL_LUNG_CANCER_99.png]

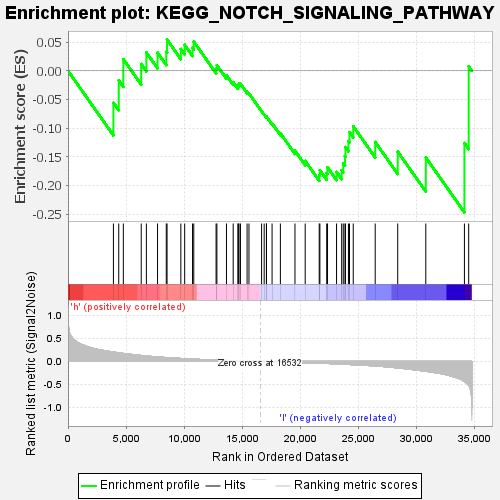

Supplement: Supplementary file 2 — Material S2. [file JCMM-28-e70079-s002.zip › 7.GSEA analysis/1.ARL11/enplot_KEGG_NOTCH_SIGNALING_PATHWAY_288.png]

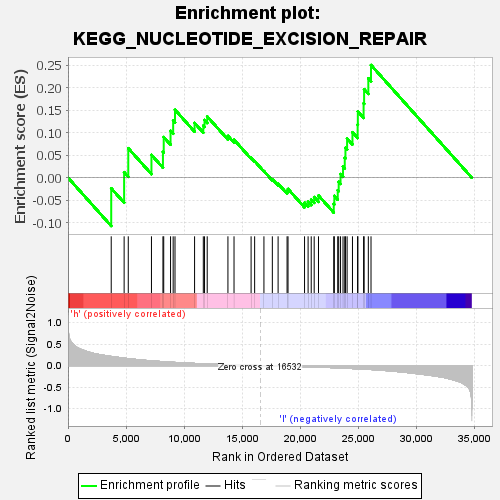

Supplement: Supplementary file 2 — Material S2. [file JCMM-28-e70079-s002.zip › 7.GSEA analysis/1.ARL11/enplot_KEGG_NUCLEOTIDE_EXCISION_REPAIR_30.png]

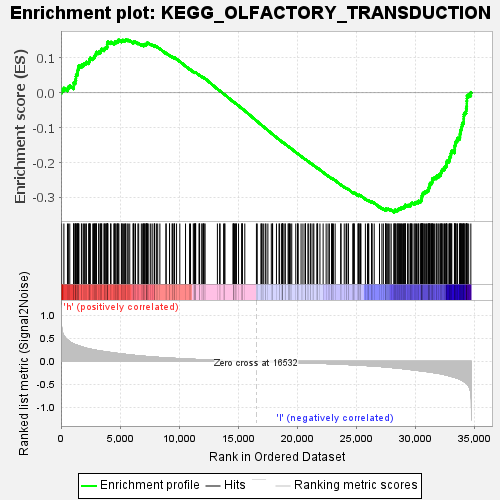

Supplement: Supplementary file 2 — Material S2. [file JCMM-28-e70079-s002.zip › 7.GSEA analysis/1.ARL11/enplot_KEGG_OLFACTORY_TRANSDUCTION_195.png]

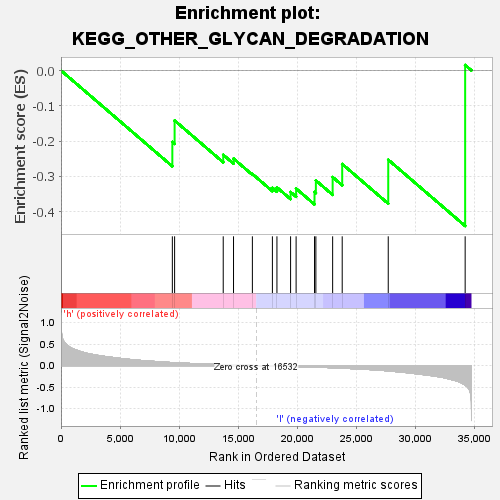

Supplement: Supplementary file 2 — Material S2. [file JCMM-28-e70079-s002.zip › 7.GSEA analysis/1.ARL11/enplot_KEGG_OTHER_GLYCAN_DEGRADATION_159.png]

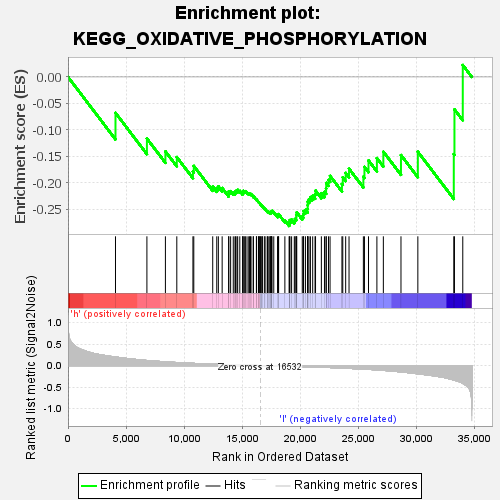

Supplement: Supplementary file 2 — Material S2. [file JCMM-28-e70079-s002.zip › 7.GSEA analysis/1.ARL11/enplot_KEGG_OXIDATIVE_PHOSPHORYLATION_180.png]

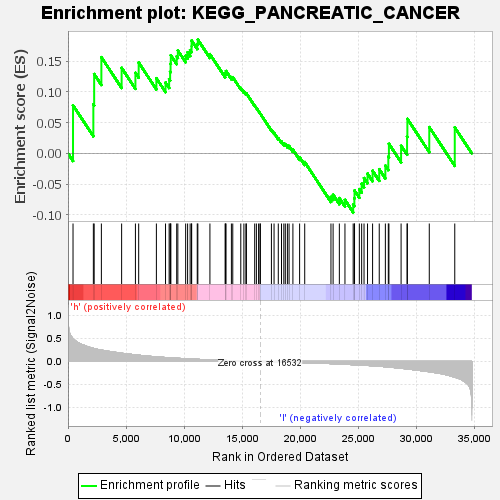

Supplement: Supplementary file 2 — Material S2. [file JCMM-28-e70079-s002.zip › 7.GSEA analysis/1.ARL11/enplot_KEGG_PANCREATIC_CANCER_141.png]

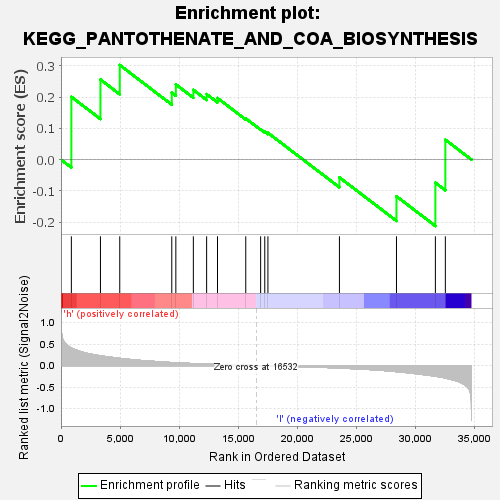

Supplement: Supplementary file 2 — Material S2. [file JCMM-28-e70079-s002.zip › 7.GSEA analysis/1.ARL11/enplot_KEGG_PANTOTHENATE_AND_COA_BIOSYNTHESIS_144.png]

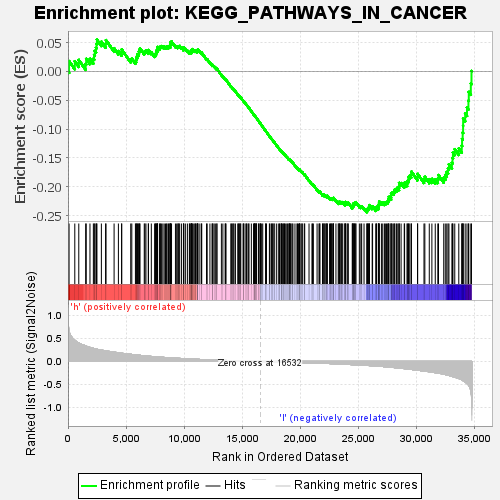

Supplement: Supplementary file 2 — Material S2. [file JCMM-28-e70079-s002.zip › 7.GSEA analysis/1.ARL11/enplot_KEGG_PATHWAYS_IN_CANCER_234.png]

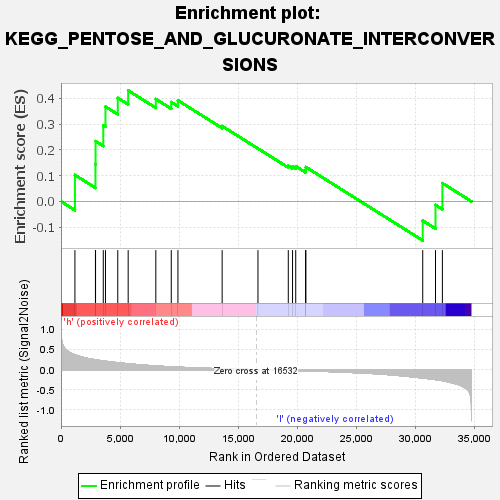

Supplement: Supplementary file 2 — Material S2. [file JCMM-28-e70079-s002.zip › 7.GSEA analysis/1.ARL11/enplot_KEGG_PENTOSE_AND_GLUCURONATE_INTERCONVERSIONS_24.png]

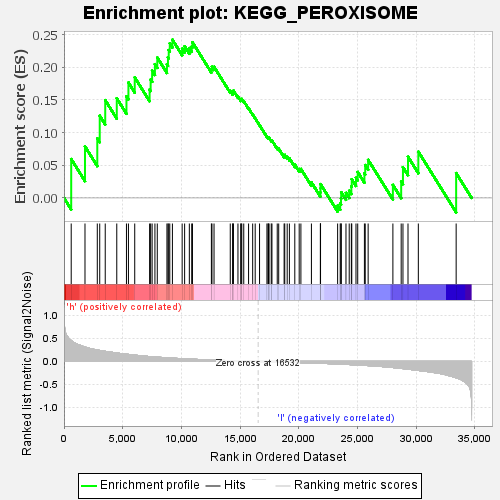

Supplement: Supplementary file 2 — Material S2. [file JCMM-28-e70079-s002.zip › 7.GSEA analysis/1.ARL11/enplot_KEGG_PEROXISOME_69.png]

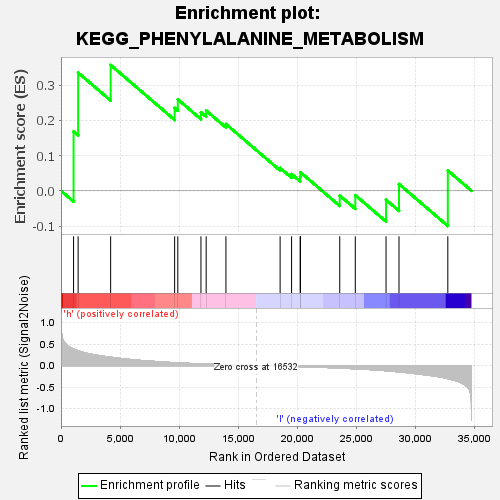

Supplement: Supplementary file 2 — Material S2. [file JCMM-28-e70079-s002.zip › 7.GSEA analysis/1.ARL11/enplot_KEGG_PHENYLALANINE_METABOLISM_42.png]

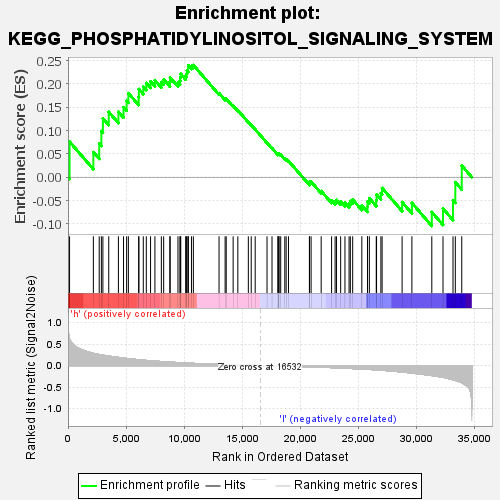

Supplement: Supplementary file 2 — Material S2. [file JCMM-28-e70079-s002.zip › 7.GSEA analysis/1.ARL11/enplot_KEGG_PHOSPHATIDYLINOSITOL_SIGNALING_SYSTEM_33.png]

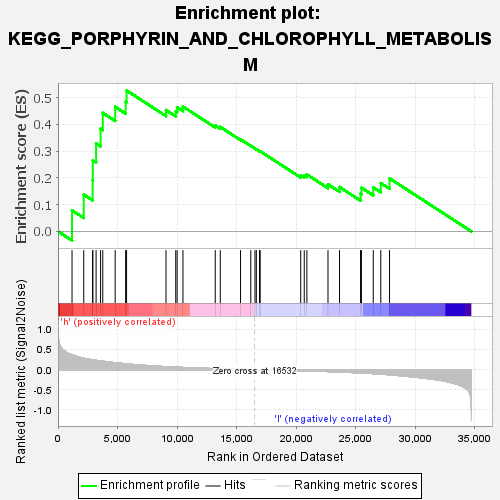

Supplement: Supplementary file 2 — Material S2. [file JCMM-28-e70079-s002.zip › 7.GSEA analysis/1.ARL11/enplot_KEGG_PORPHYRIN_AND_CHLOROPHYLL_METABOLISM_3.png]

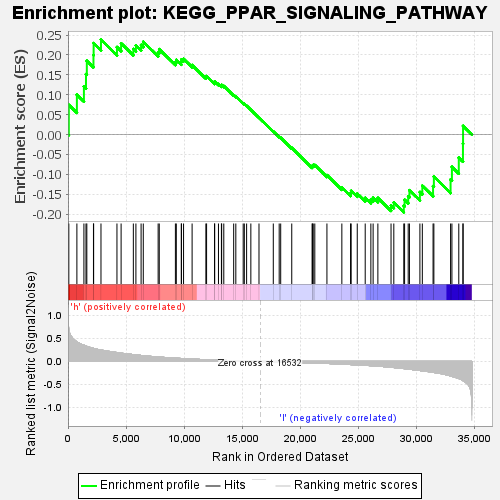

Supplement: Supplementary file 2 — Material S2. [file JCMM-28-e70079-s002.zip › 7.GSEA analysis/1.ARL11/enplot_KEGG_PPAR_SIGNALING_PATHWAY_147.png]

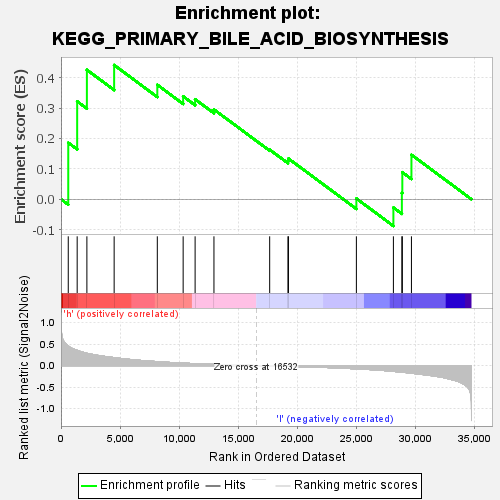

Supplement: Supplementary file 2 — Material S2. [file JCMM-28-e70079-s002.zip › 7.GSEA analysis/1.ARL11/enplot_KEGG_PRIMARY_BILE_ACID_BIOSYNTHESIS_36.png]

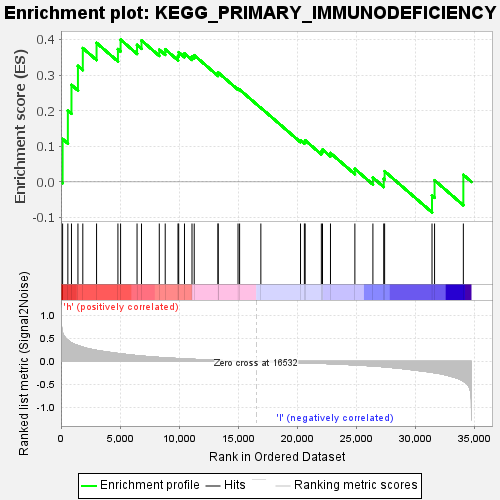

Supplement: Supplementary file 2 — Material S2. [file JCMM-28-e70079-s002.zip › 7.GSEA analysis/1.ARL11/enplot_KEGG_PRIMARY_IMMUNODEFICIENCY_27.png]

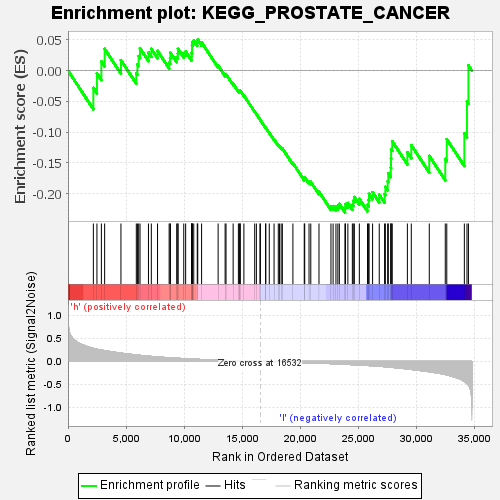

Supplement: Supplementary file 2 — Material S2. [file JCMM-28-e70079-s002.zip › 7.GSEA analysis/1.ARL11/enplot_KEGG_PROSTATE_CANCER_267.png]

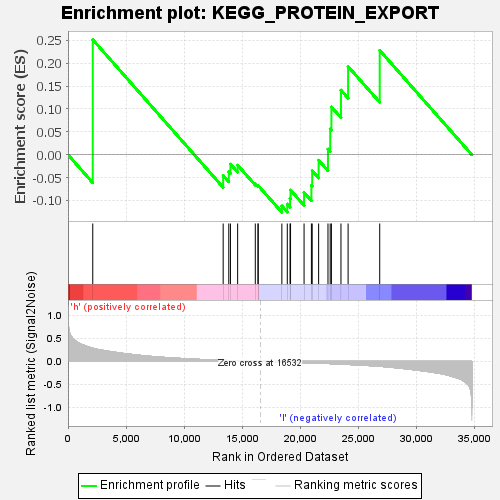

Supplement: Supplementary file 2 — Material S2. [file JCMM-28-e70079-s002.zip › 7.GSEA analysis/1.ARL11/enplot_KEGG_PROTEIN_EXPORT_129.png]

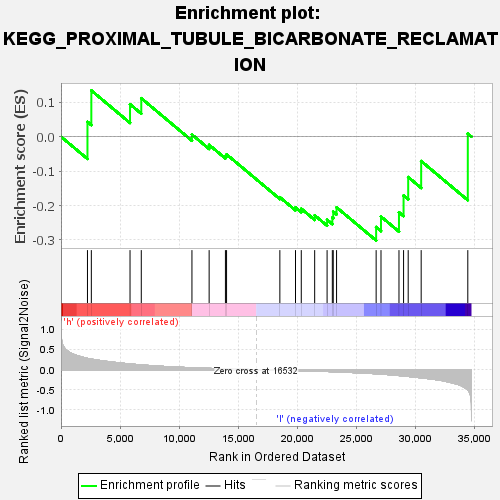

Supplement: Supplementary file 2 — Material S2. [file JCMM-28-e70079-s002.zip › 7.GSEA analysis/1.ARL11/enplot_KEGG_PROXIMAL_TUBULE_BICARBONATE_RECLAMATION_297.png]

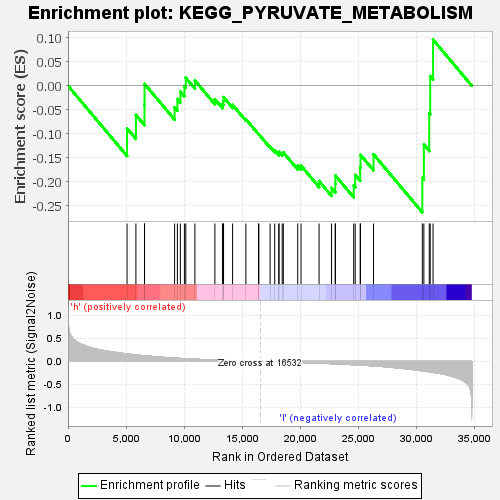

Supplement: Supplementary file 2 — Material S2. [file JCMM-28-e70079-s002.zip › 7.GSEA analysis/1.ARL11/enplot_KEGG_PYRUVATE_METABOLISM_243.png]

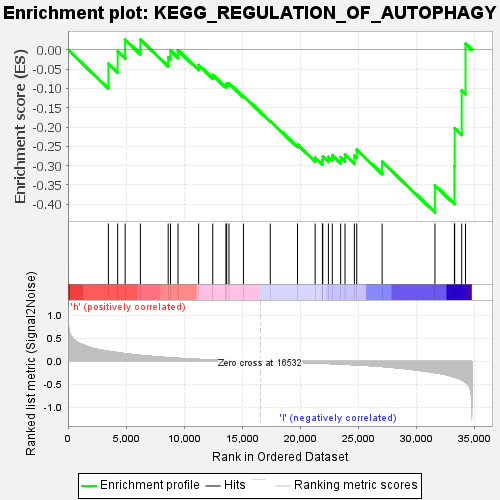

Supplement: Supplementary file 2 — Material S2. [file JCMM-28-e70079-s002.zip › 7.GSEA analysis/1.ARL11/enplot_KEGG_REGULATION_OF_AUTOPHAGY_162.png]

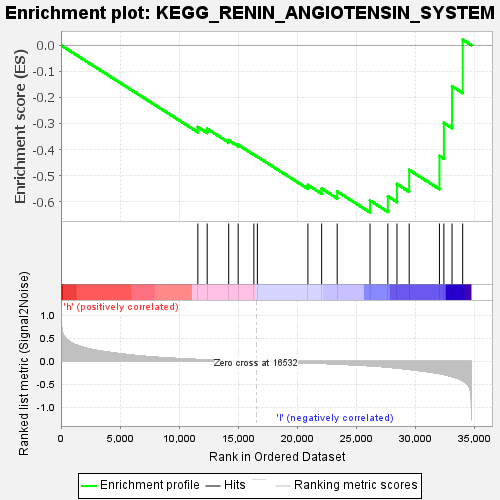

Supplement: Supplementary file 2 — Material S2. [file JCMM-28-e70079-s002.zip › 7.GSEA analysis/1.ARL11/enplot_KEGG_RENIN_ANGIOTENSIN_SYSTEM_153.png]

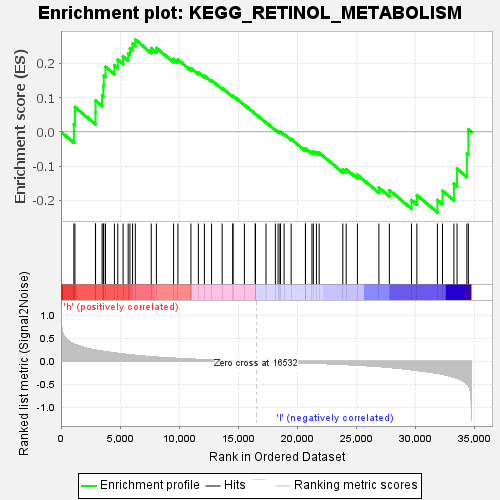

Supplement: Supplementary file 2 — Material S2. [file JCMM-28-e70079-s002.zip › 7.GSEA analysis/1.ARL11/enplot_KEGG_RETINOL_METABOLISM_102.png]

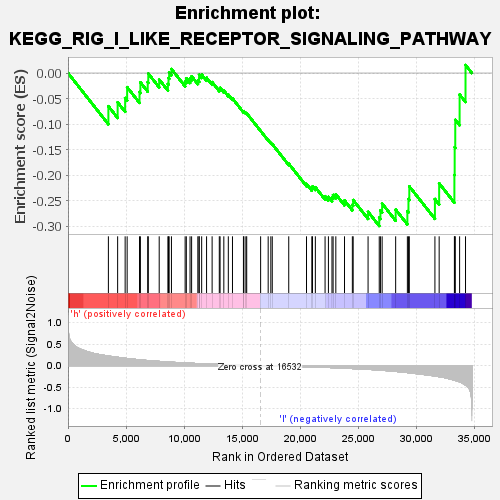

Supplement: Supplementary file 2 — Material S2. [file JCMM-28-e70079-s002.zip › 7.GSEA analysis/1.ARL11/enplot_KEGG_RIG_I_LIKE_RECEPTOR_SIGNALING_PATHWAY_207.png]

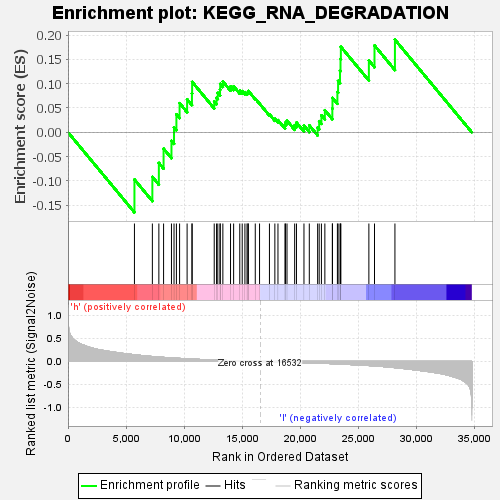

Supplement: Supplementary file 2 — Material S2. [file JCMM-28-e70079-s002.zip › 7.GSEA analysis/1.ARL11/enplot_KEGG_RNA_DEGRADATION_39.png]

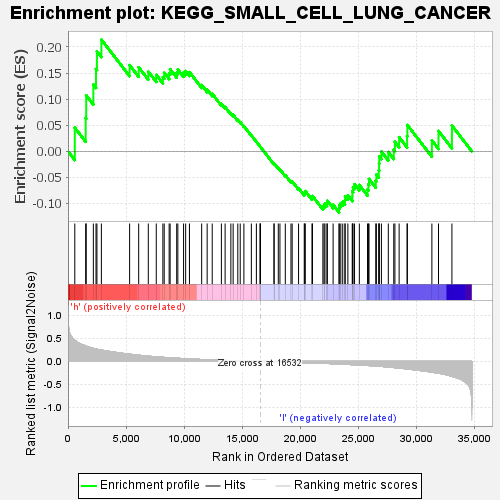

Supplement: Supplementary file 2 — Material S2. [file JCMM-28-e70079-s002.zip › 7.GSEA analysis/1.ARL11/enplot_KEGG_SMALL_CELL_LUNG_CANCER_120.png]

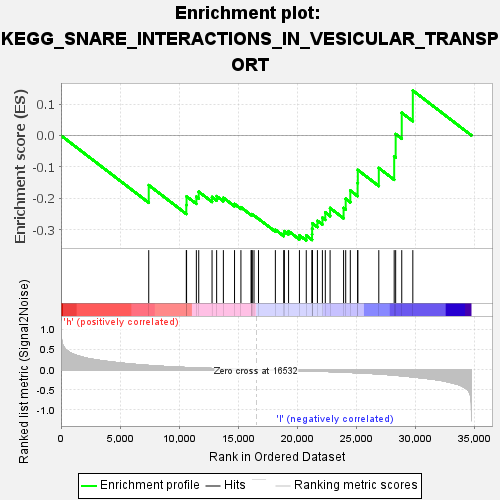

Supplement: Supplementary file 2 — Material S2. [file JCMM-28-e70079-s002.zip › 7.GSEA analysis/1.ARL11/enplot_KEGG_SNARE_INTERACTIONS_IN_VESICULAR_TRANSPORT_192.png]

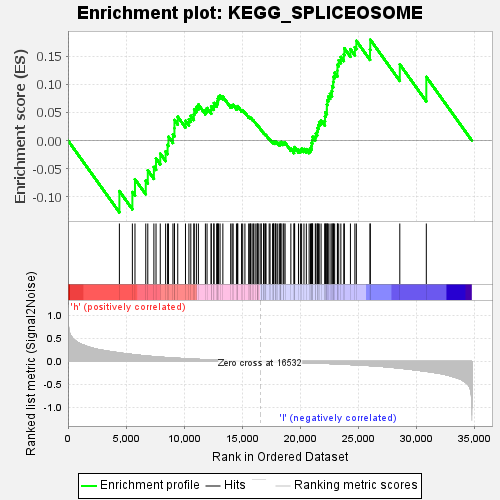

Supplement: Supplementary file 2 — Material S2. [file JCMM-28-e70079-s002.zip › 7.GSEA analysis/1.ARL11/enplot_KEGG_SPLICEOSOME_150.png]

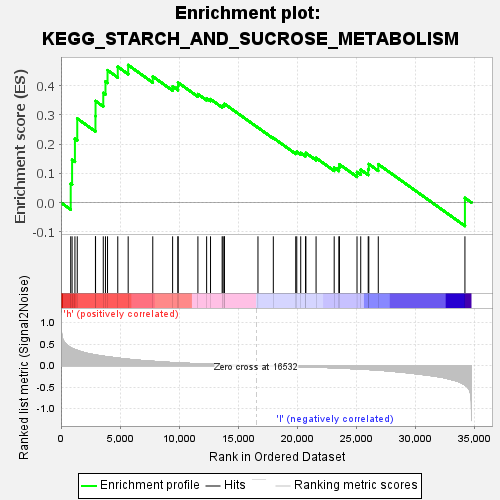

Supplement: Supplementary file 2 — Material S2. [file JCMM-28-e70079-s002.zip › 7.GSEA analysis/1.ARL11/enplot_KEGG_STARCH_AND_SUCROSE_METABOLISM_12.png]

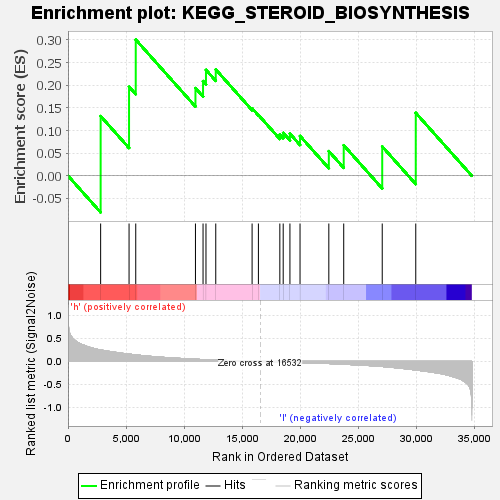

Supplement: Supplementary file 2 — Material S2. [file JCMM-28-e70079-s002.zip › 7.GSEA analysis/1.ARL11/enplot_KEGG_STEROID_BIOSYNTHESIS_87.png]

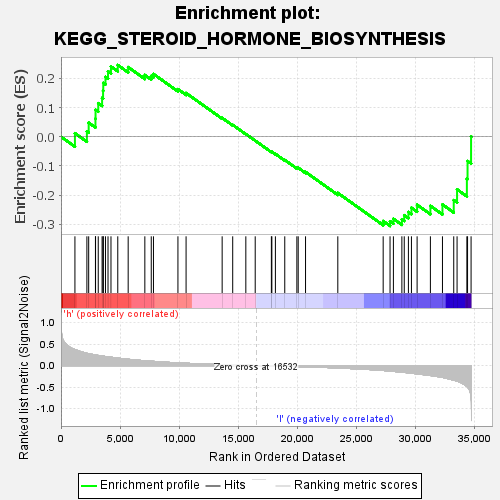

Supplement: Supplementary file 2 — Material S2. [file JCMM-28-e70079-s002.zip › 7.GSEA analysis/1.ARL11/enplot_KEGG_STEROID_HORMONE_BIOSYNTHESIS_279.png]

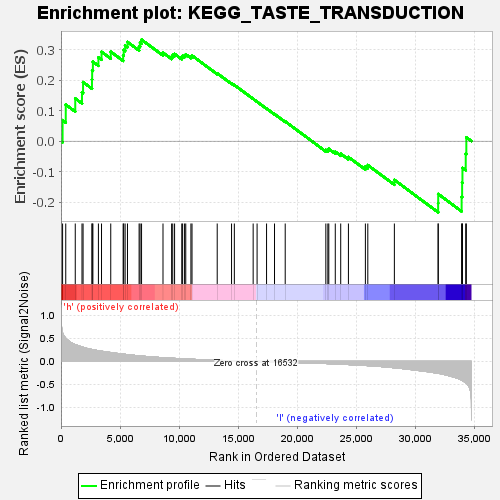

Supplement: Supplementary file 2 — Material S2. [file JCMM-28-e70079-s002.zip › 7.GSEA analysis/1.ARL11/enplot_KEGG_TASTE_TRANSDUCTION_51.png]

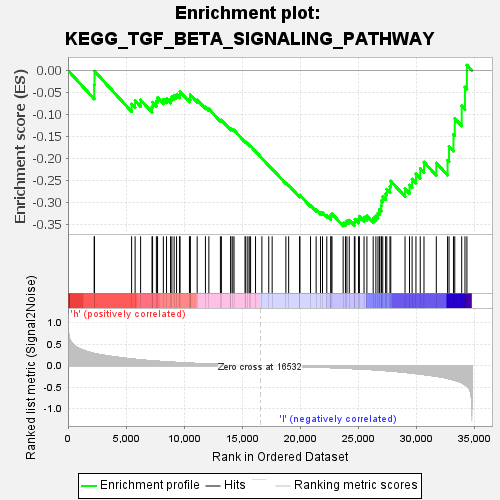

Supplement: Supplementary file 2 — Material S2. [file JCMM-28-e70079-s002.zip › 7.GSEA analysis/1.ARL11/enplot_KEGG_TGF_BETA_SIGNALING_PATHWAY_186.png]

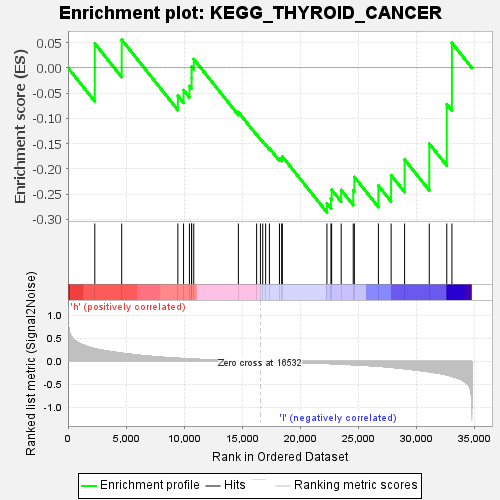

Supplement: Supplementary file 2 — Material S2. [file JCMM-28-e70079-s002.zip › 7.GSEA analysis/1.ARL11/enplot_KEGG_THYROID_CANCER_264.png]

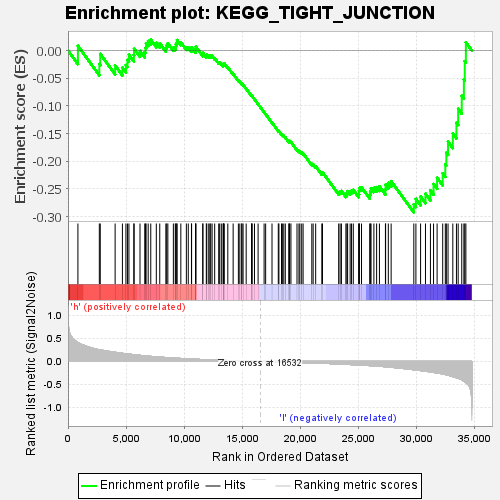

Supplement: Supplementary file 2 — Material S2. [file JCMM-28-e70079-s002.zip › 7.GSEA analysis/1.ARL11/enplot_KEGG_TIGHT_JUNCTION_213.png]

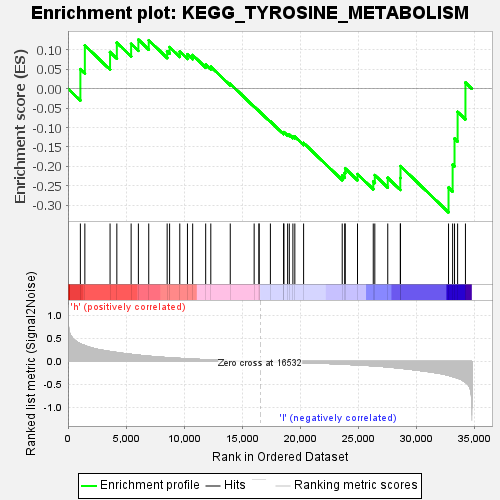

Supplement: Supplementary file 2 — Material S2. [file JCMM-28-e70079-s002.zip › 7.GSEA analysis/1.ARL11/enplot_KEGG_TYROSINE_METABOLISM_240.png]

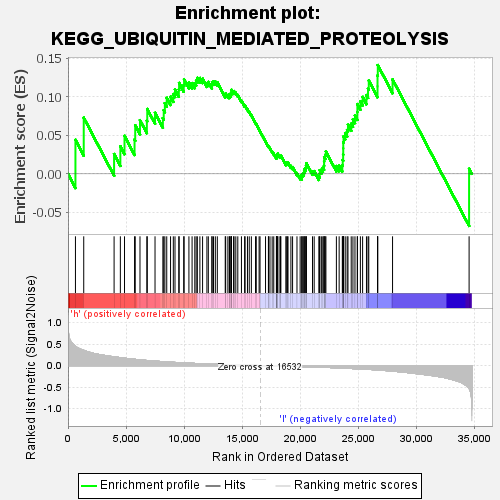

Supplement: Supplementary file 2 — Material S2. [file JCMM-28-e70079-s002.zip › 7.GSEA analysis/1.ARL11/enplot_KEGG_UBIQUITIN_MEDIATED_PROTEOLYSIS_126.png]

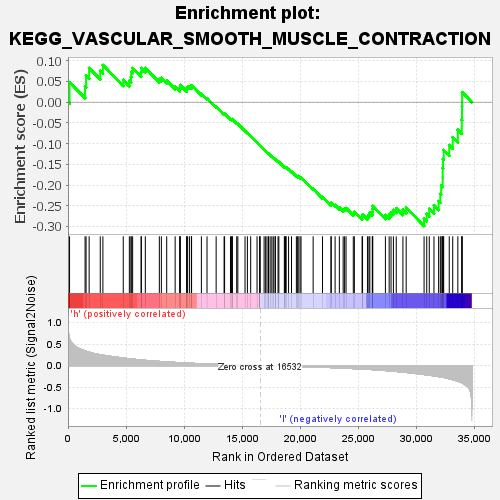

Supplement: Supplementary file 2 — Material S2. [file JCMM-28-e70079-s002.zip › 7.GSEA analysis/1.ARL11/enplot_KEGG_VASCULAR_SMOOTH_MUSCLE_CONTRACTION_228.png]

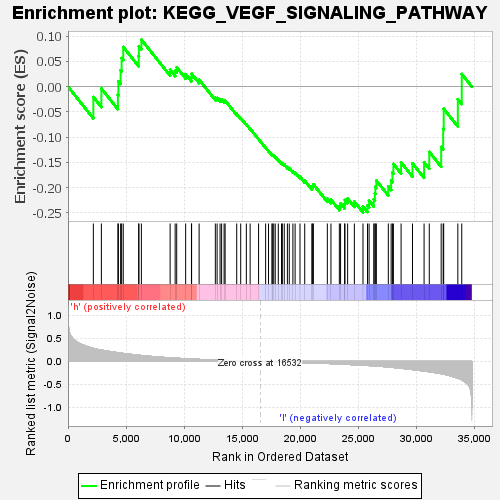

Supplement: Supplementary file 2 — Material S2. [file JCMM-28-e70079-s002.zip › 7.GSEA analysis/1.ARL11/enplot_KEGG_VEGF_SIGNALING_PATHWAY_285.png]

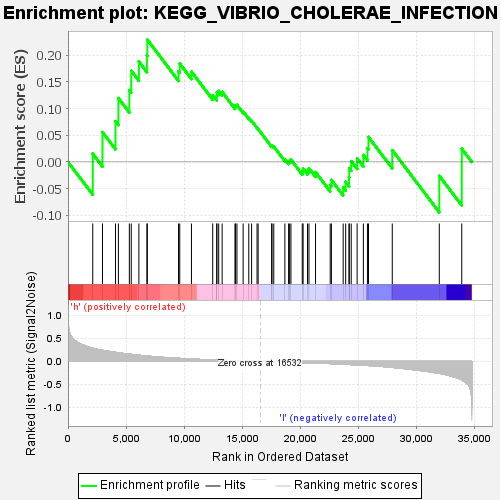

Supplement: Supplementary file 2 — Material S2. [file JCMM-28-e70079-s002.zip › 7.GSEA analysis/1.ARL11/enplot_KEGG_VIBRIO_CHOLERAE_INFECTION_60.png]
